# Modeling early phenotypes of Parkinson’s disease by age-induced midbrain-striatum assembloids

**DOI:** 10.1038/s42003-024-07273-4

**Published:** 2024-11-23

**Authors:** Kyriaki Barmpa, Claudia Saraiva, Diego Lopez-Pigozzi, Gemma Gomez-Giro, Elisa Gabassi, Sarah Spitz, Konstanze Brandauer, Juan E. Rodriguez Gatica, Paul Antony, Graham Robertson, Rahman Sabahi-Kaviani, Alessandro Bellapianta, Florentia Papastefanaki, Regina Luttge, Ulrich Kubitscheck, Ahmad Salti, Peter Ertl, Mario Bortolozzi, Rebecca Matsas, Frank Edenhofer, Jens C. Schwamborn

**Affiliations:** 1 Developmental and Cellular Biology, Luxembourg Centre for Systems Biomedicine, University of Luxembourg, Esch-sur-Alzette, Luxembourg; 2 Department of Physics and Astronomy “G. Galilei”, University of Padua, Padua, Italy; 3 Veneto Institute of Molecular Medicine (VIMM), Padua, Italy; 4 Genomics, Stem Cell & Regenerative Medicine Group and CMBI, Institute of Molecular Biology, University of Innsbruck, Innsbruck, Austria; 5 Institute of Applied Synthetic Chemistry, Vienna University of Technology, Vienna, Austria; 6 Clausius Institute of Physical and Theoretical Chemistry, University of Bonn, Bonn, Germany; 7 Eindhoven University of Technology, Microsystems, Eindhoven, Netherlands; 8 Johannes Kepler University Linz, Kepler University Hospital, University Clinic for Ophthalmology and Optometry, Linz, Austria; 9 Laboratory of Cellular and Molecular Neurobiology-Stem Cells, Hellenic Pasteur Institute, Athens, Greece; 10 Human Embryonic and Induced Pluripotent Stem Cell Unit, Hellenic Pasteur Institute, Athens, Greece

**Keywords:** Cellular neuroscience, Neural ageing, Parkinson's disease, Ageing, Stem-cell differentiation

## Abstract

Parkinson’s disease, an aging-associated neurodegenerative disorder, is characterised by nigrostriatal pathway dysfunction caused by the gradual loss of dopaminergic neurons in the substantia nigra pars compacta of the midbrain. Human in vitro models are enabling the study of the dopaminergic neurons’ loss, but not the dysregulation within the dopaminergic network in the nigrostriatal pathway. Additionally, these models do not incorporate aging characteristics which potentially contribute to the development of Parkinson’s disease. Here we present a nigrostriatal pathway model based on midbrain-striatum assembloids with inducible aging. We show that these assembloids can develop characteristics of the nigrostriatal connectivity, with catecholamine release from the midbrain to the striatum and synapse formation between midbrain and striatal neurons. Moreover, Progerin-overexpressing assembloids acquire aging traits that lead to early neurodegenerative phenotypes. This model shall help to reveal the contribution of aging as well as nigrostriatal connectivity to the onset and progression of Parkinson’s disease.

## Abstract (web summary)

Generation of a midbrain-striatum assembloid with progerin overexpression as an in vitro model of possessing aging and senescence characteristics for studying Parkinson’s disease.

## Introduction

The nigrostriatal pathway connectivity is established through projections of the midbrain substantia nigra pars compacta (SNpc) dopaminergic neurons (DANs) to the dorsal striatum. Disruption of the nigrostriatal connectivity contributes to the development of Parkinson’s disease (PD) symptoms, such as motor dysfunction, tremor, muscle stiffness, and bradykinesia^1^. The gradual loss of DANs leads to dopamine depletion in the striatum, with consequences in the synaptic network organisation and cellular complexity^2^. Before the onset of neuronal loss there is a significant decrease of dopaminergic axons in the striatum. This reduction in dopaminergic axons is twice as pronounced in the striatum compared to the loss in dopaminergic neuronal cells in the SNpc^3,4^. However, due to the research focus on the loss of neurons, which is the ultimate endpoint of the disease, the mechanisms of the retrograde dopaminergic axons’ degeneration from the striatum are still not understood^5^.

Animal models of PD have been used to elucidate cellular and molecular dysfunctions, but they cannot fully recapitulate the impairments that occur in the human nigrostriatal connectivity^6^. More recently, human based cellular models such as induced pluripotent stem cells (iPSCs) derived 2D neuronal cell cultures and the more advanced 3D midbrain organoids (MOs) have been extensively used for PD modelling. MOs have the advantage of better recapitulating the neuronal connectivity in a 3D environment with active neuronal electrophysiology and self-organisation properties^7,8^. MOs derived from PD patient cell lines exhibit phenotypes relevant to the disease and they present a great tool for further understanding the molecular and cellular mechanisms of neurodegeneration^9–12^. Although these models are useful for studying the dopaminergic neurons’ vulnerability in PD, they do not recapitulate the nigrostriatal pathway connectivity dysfunction which is essential for unveiling the sources of PD onset and progression.

More advanced 3D models with the combination of two or more region-specific brain organoids have started to pave the way towards cellular models with higher complexity that can effectively reflect the neuronal connectivity between different brain regions^13^. For instance, recent studies have shown the generation of pallium-subpallium, cortico-motor and cortico-striatal assembloids from human iPSCs^14–16^, and their capability to develop physiological functional networks that could provide essential insights in diseases’ development and progression.

Here we present the development of a midbrain-striatum assembloid model that can recapitulate the nigrostriatal pathway connectivity, with catecholamine release from the midbrain to striatum and the formation of active synapses between the midbrain DANs and the striatal neurons. Although the midbrain-striatum assembloid model allows us to study aspects of human nigrostriatal connectivity in vitro, it still lacks one major risk factor of PD, which is aging^17,18^. To address whether aging induction could lead to neurodegenerative phenotypes in assembloids, we used a genetically engineered iPSC line that carries a Progerin-GFP transgene under the control of the Tet-On system for the controllable overexpression of Progerin in the presence of doxycycline. Our results show that Progerin-overexpression can induce aging phenotypes in the midbrain-striatum assembloid model which subsequently leads to the development of PD-associated early neurodegeneration phenotypes.

## Results

### Development of the Midbrain-Striatum assembloid model

The nigrostriatal pathway is characterised by DANs’ projections from the SNpc to the putamen and caudate of the dorsal striatum. To recapitulate this pathway in vitro, we generated a 3D in vitro model of MOs and striatum organoids (StrOs). MOs were generated based on our previously published protocols^7,19^. The iPSCs and neuroepithelial stem cells (NESCs) used in this study were characterised for the expression of pluripotent and multipotent markers (Supplementary Fig. 1a–g). For the generation of StrO different conditions were tested (RA, SR and C4) and compared to the recently published protocol (named C3)^15^ with minor changes (Supplementary Fig. 2a–d). The protocols for the conditions RA, SR and C4 were generated in our lab for optimising the efficiency of StrOs generation. For the conditions RA, SR and C4 organoids were cultured for day (D)35 and D50, while C3 organoids were cultured until D50 (the earliest time point described in ref. ^15^). In C4, the differentiation conditions are similar to C3^15^, but in the maturation phase (D17 to D35), DHA was excluded as it is susceptible to oxidation and to avoid the use of ethanol (the solvent for DHA) in the medium. The same medium was used until D50 without the addition of DAPT at D42 (Supplementary Fig. 2a). DHA was also excluded from the C3 (Supplementary Fig. 2b). For conditions RA and SR (Supplementary Fig. 2c, d), we adapted a previously published protocol^20^ to shift the differentiation towards the lateral ganglionic eminence (LGE) region and eventually to the dorsal striatum. As was demonstrated by Miura and colleagues^15^, SR11237 was used to stimulate the RXRG receptor (SR condition), which is highly expressed in the developing striatum as shown in the human brain transcriptome (HBT) database. Similarly, we also tested the effects of RA supplementation (RA condition) as data in the HBT database show that RARB is also highly expressed early in striatum development.

qPCR analysis confirmed organoids’ differentiation towards the dorsal striatum (Supplementary Fig. 3a). While the forebrain marker Forkhead box protein G1 (*FOXG1*) was consistently expressed, the higher expression of Achaete-Scute Family BHLH Transcription Factor 1 (*ASCL1*) in the RA and SR conditions indicates a slower differentiation of cells with a higher number of progenitors at D50 of culture. Contrary, *ASCL1* expression was lower in D50 C4 and C3. Genetic-Screened Homeobox 2 (*GSX2*), an essential transcription factor (TF) in LGE progenitors^21^, was mainly expressed in D50 C4 and C3. The expression of Forkhead Box P1 (*FOXP1)* was similar in all conditions, but Forkhead Box P2 (*FOXP2*) was higher in D50 C4 and C3, indicating a better differentiation towards medium spiny neurons’ (MSNs) fate as both genes encode important TFs for the development of MSNs^22^. Additionally, COUP-TF-interacting protein 2 (*CTIP2*), another crucial TF for MSNs’ differentiation^23^, showed higher expression in C4 and C3. Expression of NK2 Homeobox 1 (*NKX2.1)* and Orthodenticle Homeobox 2 (*OTX2)* is essential for the development of the medial ganglionic eminence^24^. Their low expression in C3 and C4 suggests a LGE and dorsal striatum identity (Supplementary Fig. 3b). The presence of mature MSNs was evaluated by the expression of Dopamine and cAMP-Regulated Neuronal Phosphoprotein 32 (*DARPP32*) and the dopaminergic receptors 1 and 2 (*DRD1* and *DRD2*). C4 had higher expression of *DARPP32*, while *DRD1* and *DRD2* were similarly expressed in C3 and C4 (Supplementary Fig. 3c). Likewise, DARPP32 protein abundance was similar at D50 C3 and C4, DRD1 was higher at D50 C3 and C4 and there were no differences in the levels of DRD2. Glutamic acid decarboxylase 65-kilodalton isoform (GAD65), an enzyme important for the synthesis of GABA^25^, was higher in C3. Overall, these results indicate that C4 and C3 have similar differentiation capacity towards dorsal striatum identity (Supplementary Fig. 3d–g). C4 seems to result in progenitor-rich striatum organoids at D35 with differentiated characteristics at D50.

**Fig. 1:**
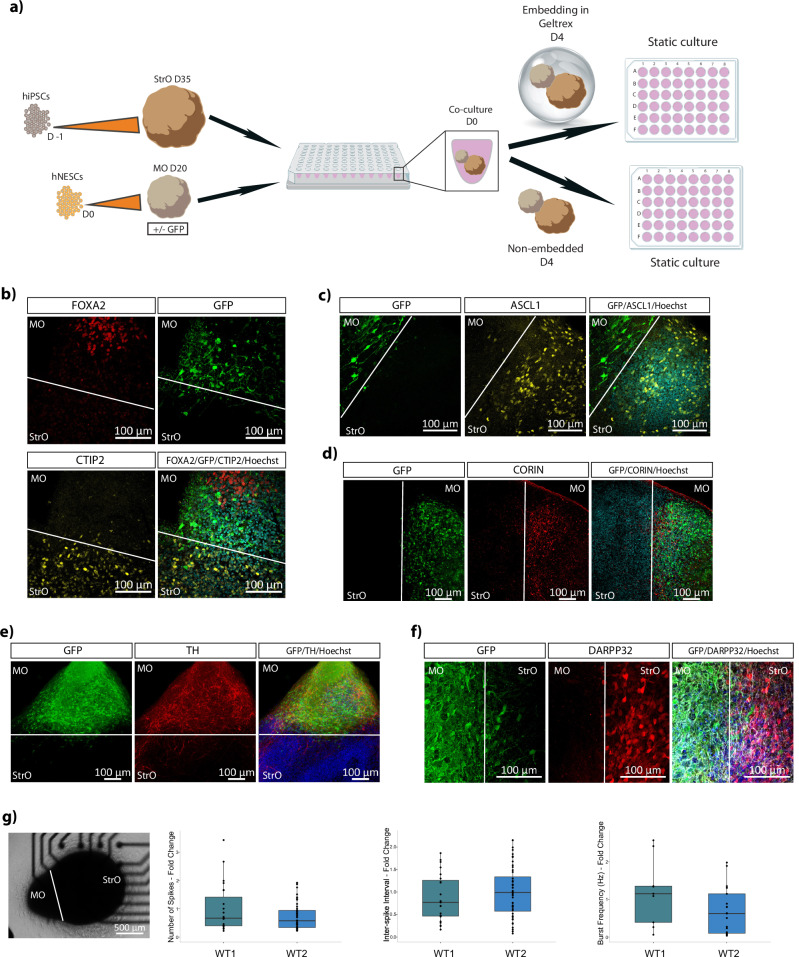
Generation of midbrain-striatum assembloid model with identity specificity. **a** Schematic representation of the assembloid model generation. **b** Representative confocal image of a 70 μm assembloid section immunostained with Hoechst, FOXA2 visible in the MO side and CTIP2 visible in the StrO side of the assembloid. GFP fluorescence is intrinsic in the midbrain part of the assembloids. **c** Representative confocal image of a 70 μm assembloid section immunostained with Hoechst and ASCL1 visible in the StrO side of the assembloid. GFP fluorescence is intrinsic in the MO part of the assembloids. **d** Representative confocal image of a 70 μm assembloid section immunostained with Hoechst and CORIN visible in the MO side of the assembloid. GFP fluorescence is intrinsic in the midbrain part of the assembloids. **e** Representative confocal image of a 70 μm assembloid section immunostained with Hoechst and TH visible in the MO side of the assembloid. GFP fluorescence is intrinsic in the midbrain part of the assembloids. **f** Representative confocal image of a 70 μm assembloid section immunostained with Hoechst and DARPP32 visible in the StrO side of the assembloid. GFP fluorescence is intrinsic in the midbrain part of the assembloids. **g** Representative brightfield image of an assembloid in a well of the 48-well MEA plate. Plots showing the quantification of the Number of Spikes, Inter-spike Interval in seconds and the Burst Frequency in Hz between assembloids generated from two independent human WT cell lines. The data in the plots represent recordings of individual assembloids for the culture periods D32 to D43, from 3 to 4 batches. Batch correction was applied by normalising each value to the mean of the values for each batch. Outliers were calculated in GraphPad Prism using the ROUT method Q 1%. Two-sided Wilcoxon test was performed in R 4.2.2.

Due to MOs and StrOs independent differentiation, the generation of the assembloid model required an optimised co-culture medium. Assembloids were generated between D20 GFP-expressing MOs and D35 or D50 StrOs (Supplementary Fig. 4a) from the four StrO conditions described before. Four different media were tested to ensure the optimal development and identity specificity of both organoids (Supplementary Fig. 4b). TH and MAP2 immunofluorescence staining of MOs cultured in NM and NMpl media showed significant depletion of DANs, while MOs in NM++ and N2B27++ had no differences from the control condition (Supplementary Fig. 4c). The four different media were also tested in D20 and D35 assembloids to assess the impact on both DANs (TH-positive) and MSNs (DARPP32-positive). Assembloids in NM++ and N2B27++ media showed higher TH-positive neurons, specifically in C4 and C3 (Supplementary Fig. 5). N2B27++ medium was considered optimal for assembloid culture, due to favourable development of TH and DARPP32-positive neurons in C4 (Supplementary Fig. 6) (Fig. 1a). Based on these data, for reducing the culture time and achieving the desired differentiation capacity in assembloids, StrOs generated with the strategy C4 at D35 of differentiation were used for assembloids generation in the subsequent experiments.

### Midbrain and striatum specific identity in the assembloid model

Characterisation was performed on D30 assembloids. Midbrain progenitors positive for FOXA2 and CORIN were identified in the midbrain side of assembloids, while striatal progenitors positive for ASCL1 and CTIP2 appear on the striatum side (Fig. 1b–d). Mature TH and DARPP32-positive neurons of midbrain and striatal identity were also observed (Fig. 1e, f). Neuronal activity in assembloids generated from the two independent WT cell lines was validated by MEA electrophysiological measurements. Assembloids from both lines displayed similar electrophysiological activity with no differences in the number of spikes, the interspike interval and the burst frequency (Fig. 1g).

**Fig. 2:**
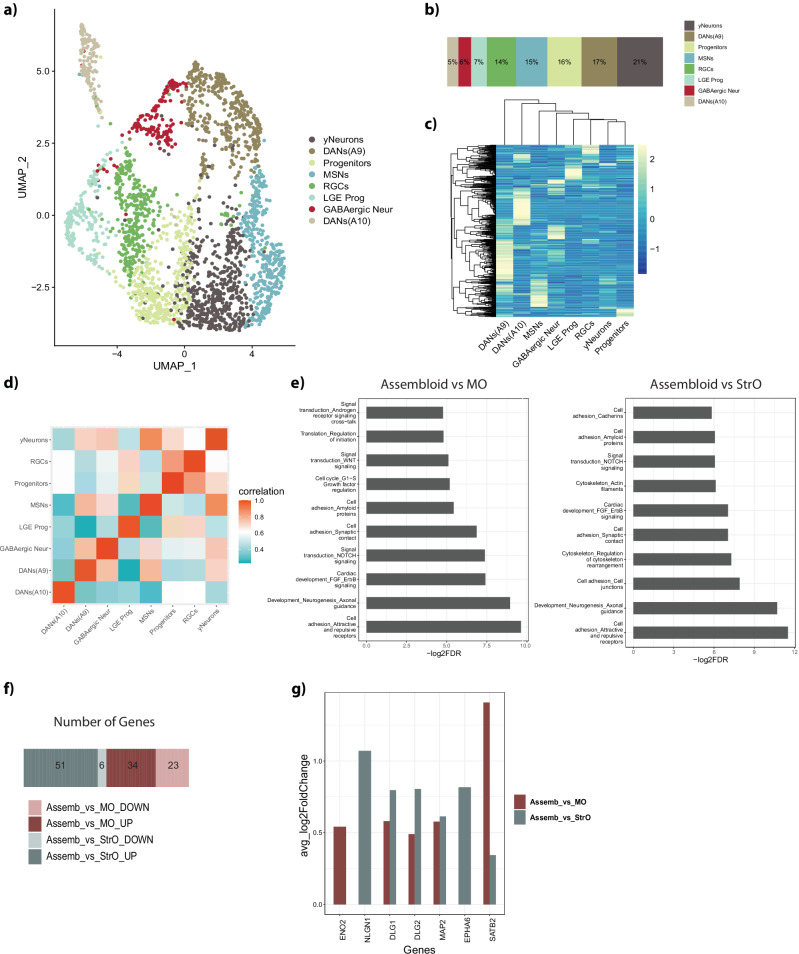
Single nuclei RNA sequencing analysis in the assembloid model. **a** UMAP embedding of the different cellular clusters in assembloids. **b** Percentage of the cellular composition in the assembloid model. **c** Unsupervised hierarchical clustering of cell clusters, using the average expression with *Z*-score normalisation of the top 500 most variable genes. **d** Spearman’s correlation between the different cell types in assembloids. **e** Enriched pathways identified by Metacore using the DEGs between assembloid and MO, and between assembloid and StrO, after integration of the three datasets with the Seurat workflow. **f** Plot showing the number of genes that were identified in the enrichment of the “Developmental Neurogenesis and Axonal guidance” pathway in both comparisons Assembloid vs MO and Assembloid vs StrO. **g** Barplot showing the upregulation of neuronal maturity related genes in both DEG lists, of Assembloid vs MO and Assembloid vs StrO.

To confirm the qualitative observations, we performed single nuclei RNA sequencing on D30 assembloids. We also sequenced MOs and StrOs that after D20 and D35 respectively were cultured independently for 30 more days in the assembloid co-culture medium. Datasets from the three models were analysed separately following the standard Seurat workflow. Eight different clusters were identified in assembloids and visualised with UMAP (Fig. 2a). The clusters’ identity was defined based on the expression of specific markers from gene lists identified in the literature^26–28^ and the PanglaoDB database (Supplementary Fig. 7). Calculation of the percentage of each cellular identity revealed the presence of 5% DANs(A10), 6% GABAergic neurons, 7% progenitors of the LGE (LGE Prog), 14% radial glia cells (RGCs), 15% MSNs, 16% non-defined progenitors (Progenitors), 17% DANs(A9) and 21% young neurons (yNeurons) (Fig. 2b). Hierarchical clustering using variable genes demonstrated the similar transcriptomic identity of the DANs clusters, followed by mature neuronal populations of MSNs and GABAergic neurons, while young neurons and progenitors cluster together (Fig. 2c). Spearman’s correlation matrix highlighted close association between progenitors and their weaker correlation with mature populations. Additionally, A9 and A10 DANs exhibited distinct genetic signatures, with A10 DANs showing a less mature identity, and A9 DANs correlating with the more mature clusters (Fig. 2d).

The same analysis was performed for MOs and StrOs. MOs are comprised of DANs clusters (DANs and DANs2, 28% and 3% respectively) but also GABAergic neurons (33%), general neurons with no specific identity (Neurons, 14%), 20% RGCs and a small proportion of oligodendrocyte precursor cells (OPCs, 1%) (Supplementary Fig. 8a, b). Heatmap of the variable genes shows the clustering of RGCs in the middle, giving rise to the DANs, GABAergic neurons and Neurons clusters, while DANs2 and OPCs cluster separately, showing a more specific identity (Supplementary Fig. 8c). In Spearman’s correlation, DANs2 cluster shows a more mature identity with a weak correlation to RGCs and a stronger correlation with the GABAergic Neur and Neurons clusters. Contrary, DANs cluster shows a less mature identity (Supplementary Fig. 8d). The clustering was confirmed by the expression of different markers (Supplementary Fig. 8e–j).

StrOs show a striatum specific cellular composition with 42% MSNs, 39% GABAergic interneurons, 10% LGE progenitors, 8% Neural progenitors and 1% Telencephalic progenitors (Supplementary Fig. 9a, b). Hierarchical clustering of the variable genes shows the close association of the progenitor clusters, followed by the more mature populations of MSNs and GABAergic interneurons (Supplementary Fig. 9c). Spearman’s correlation matrix further confirms the maturity of the MSNs, with their weak correlation to LGE and Telencephalic progenitors, and their strong correlation to GABAergic interneurons (Supplementary Fig. 9d). The identity of each cellular cluster was also here validated by the expression of specific markers (Supplementary Fig. 9e–k).

To compare the three datasets and evaluate the differentially expressed genes (DEGs) between assembloids and the individual organoids, we performed integration analysis using the Seurat workflow^29^. A clear shift of cellular populations from the MOs and StrOs into the assembloid model is visible in the UMAP plot of the integrated object based on the clusters identified separately in each model (Supplementary Fig. 10a). The progenitor clusters in all models appear in the upper part of the UMAP. DANs(A10) population clusters closer to the RGCs, illustrating once more their immature identity. Between the progenitor and mature clusters appears an intermediate more general cluster of progenitors in the assembloids model (Progenitors). This progenitor cluster seems to evolve into neuronal identity cells, given its close association with the yNeurons cluster. DANs cluster, that has a more immature identity in MOs, was not present in the assembloid model, while the general neuronal cluster in MOs, seems to have been shifted towards the DANs(A9) identity in assembloids. The MSNs cluster from StrOs, was preserved in assembloids. The strong correlation between GABAergic interneurons and MSNs is likely the reason why this cluster is not observable in the assembloid model (Supplementary Fig. 10b). A small number of OPCs is only present in MOs. Given its correlation with neuronal clusters, this cluster probably consists of cells with mixed identities.

Enrichment analysis using the DEGs of assembloids-MOs and assembloids-StrOs, revealed several processes related to synaptic contact, cell adhesion but also neurogenesis and axonal guidance (Fig. 2e). Concerning the developmental neurogenesis and axonal guidance pathway, the second most enriched pathway in both comparisons, we observed that most responsible genes for enriching this pathway are upregulated in the assembloid model (Fig. 2f). Additionally, we evaluated the expression of genes related to neuronal maturity and identified six genes, all showing upregulation in assembloids (Fig. 2g). Finally, genes related to cellular and oxidative stress were downregulated in assembloids (Supplementary Fig. 10c, Supplementary Table 5).

### Midbrain-striatum assembloid model resembles the nigrostriatal pathway connectivity

**Fig. 3:**
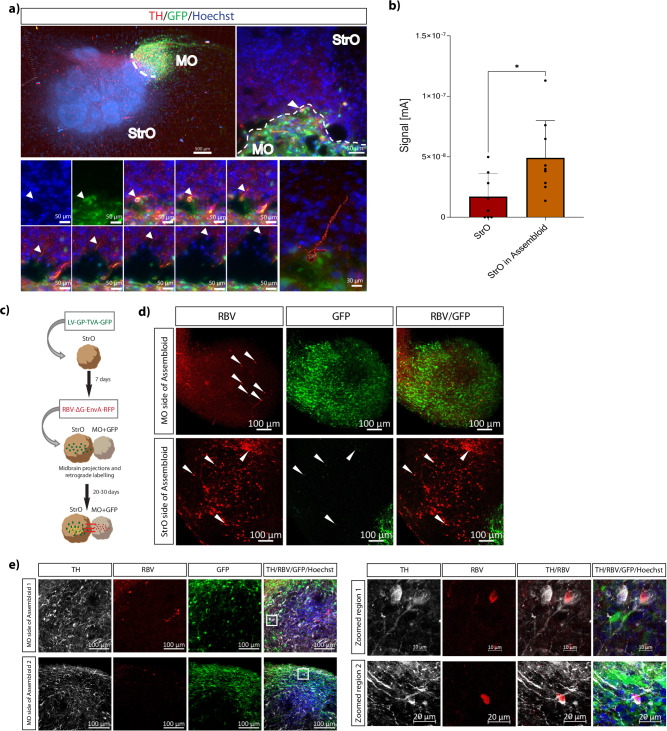
Midbrain-Striatum assembloids develop nigrostriatal pathway connectivity. **a** Representative microscopic images of a whole assembloid (4× objective) and of ROI, observed with fluorescence microscopy (25× objective). The assembloid was immunostained with Hoechst and TH. The ROI (right panel) shows the GFP+/TH+ neuron’s soma in the MO-GFP side of the assembloid with TH+ projection towards the striatum side in the different planes along the *Z* stack (*Z*.190–280). White arrowheads show the progression of the TH+ projection in images from the different planes. 3D reconstruction of the neuron across the planes shows the complete TH+ neuronal projection. **b** Bar pot showing the electrochemical measurements in tissue in StrOs and in the StrO side of the assembloid model at D30. Welch’s *t*-test was performed, StOs *n* = 8, StrOs in assembloid *n* = 9, where *n* is the average measurements in one organoid or assembloid, for three batches, generated from the same line (390, see Supplementary Table 1). Error bars represent mean ± SD. Data were plotted in GraphPad Prism 9.0.0. *p < 0.05, **p < 0.01, ***p < 0.001. **c** Schematic representation of the Rabies monosynaptic tracing experiments in midbrain-striatum assembloids. **d** Representative confocal image of 70 μm assembloid section showing the GFP and RFP positive cells from the LV-GP-TVA-GFP and RBV-ΔG-EnvA-RFP infections respectively. The GFP signal in the StrO side of the assembloid is coming from the LV-GP-TVA-GFP infection, while GFP fluorescence in the MO side of the assembloid is cell intrinsic. White arrowheads indicate the RFP positive signal in the MO side, and the RFP/GFP positive signal in the StrO side of the assembloid. **e** Representative confocal images of 70 μm assembloids sections showing the MO sides of the assembloids with GFP and RFP positive cells from the cell intrinsic GFP expression and RBV-ΔG-EnvA-RFP infections respectively and immunostained with Hoechst and TH. Zoomed in regions (indicated by the white squares) show the TH+/RFP+ colocalization in the midbrain side of the assembloids.

To validate the nigrostriatal pathway connectivity, we examined the presence of dopaminergic projections from the midbrain into the striatum in assembloids. First, to confirm that TH+ signal in the striatum of assembloids originates from the MOs TH+ neurons innervation, we examined the presence of TH+ neurons in GFP-expressing MOs and StrOs cultured separately in the assembloid co-culture media for the same culture period as the assembloids. We observed that MOs develop a high number of TH+ neurons, while the StrOs have almost no TH+ signal (Supplementary Fig. 11a). Next, for visualising the TH+ neurite projections in assembloids, we performed whole mount imaging of D30 assembloids containing GFP-expressing MOs. We were able to identify neurons with TH+/GFP+ soma indicating their midbrain origin, with TH+ axons projecting towards the striatum side of the assembloid (Fig. 3a, Supplementary Fig. 11b, c).

Nigrostriatal pathway functionality in the assembloid model was further investigated with electrochemical measurements. Catecholamine levels were assessed by inserting a Nafion-coated carbon electrode into the neuronal tissues (Supplementary Fig. 11d). We have previously shown that electrochemical monitoring of catecholamine levels in the supernatant of MOs can provide valuable insights into the presence of dopamine (DA), due to neglectable levels of the interfering cationic catecholamines norepinephrine and epinephrine within the system^30^. As expected, we observed higher levels of catecholamines in the MO side of the assembloid compared to the StrO side (Supplementary Fig. 11e). No differences in StrOs and StrOs cultured in MO-conditioned media were detected, indicating no significant catecholamine uptake from the medium (Supplementary Fig. 11f). Finally, we measured catecholamine levels in StrOs alone and StrOs in the assembloid model. The results showed that StrOs in the assembloid model displayed higher signals compared to StrOs cultured alone (Fig. 3b), suggesting the active secretion of the catecholamine DA from midbrain DANs that are projecting to the striatum in the assembloid model.

To further determine whether neurons of MO and StrO are connected through active synapses in assembloids, we established a rabies virus-based retrograde monosynaptic tracing system. StrOs at D35 of culture were transduced with LV-GP-TVA-GFP expressing the H2B-GFP fusion protein under the human Synapsin promoter. One week later, transduced StrOs were merged with MOs expressing GFP to generate assembloids, and together they were infected with RBV-ΔG-EnvA-RFP. The targeted starter neurons in the StrO were RFP+/GFP+ (Fig. 3c). Starter neurons (RFP+/GFP+, arrowheads Fig. 3d StrO side) as well as target neurons (RFP+/GFP−, arrowheads Fig. 3d StrO side) were detected in the striatum side of assembloids, demonstrating high connectivity of the nearby neurons with the starter neurons in the striatum organoid. Notably, RFP+ (arrowheads Fig. 3d MO side) target neurons were additionally present more distantly in the MO compartment, indicating active synaptic connectivity between the two organoids on the assembloid level. TH-immunostaining revealed TH+/RFP+ target neurons in the MO side of assembloids, suggesting the presence of synapse connectivity between DANs in the MO and starter neurons in the StrO (Fig. 3e). Target neurons (RFP+/GFP−) presence was also confirmed in assembloids with non-GFP-expressing MOs (Supplementary Fig. 11g).

**Fig. 4:**
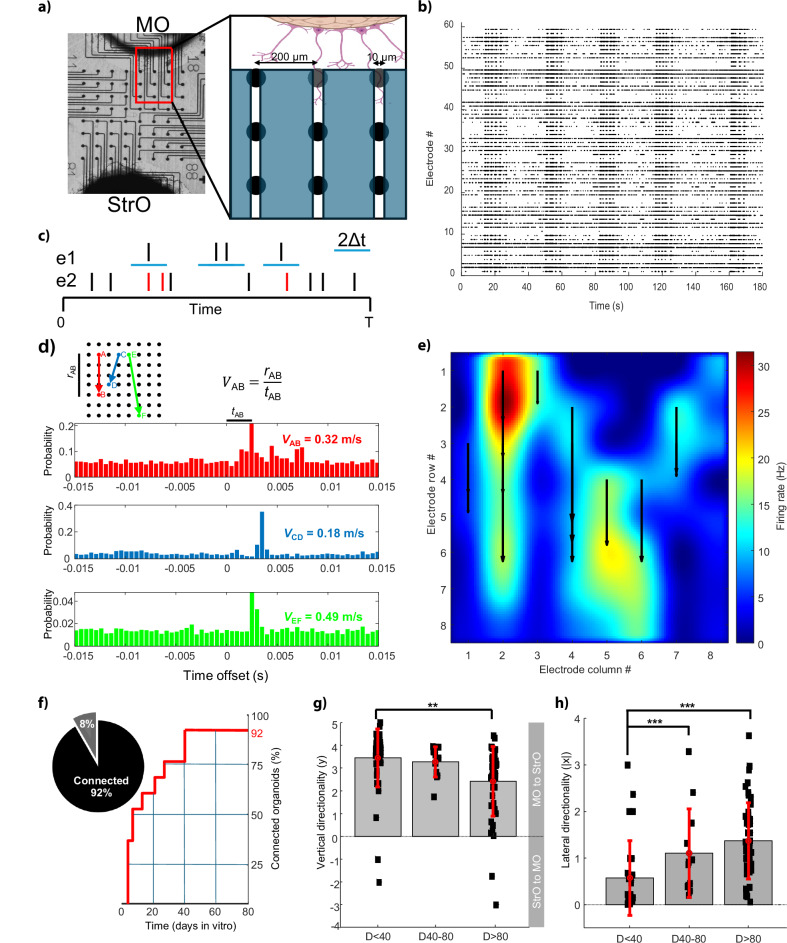
Midbrain-Striatum assembloids develop electrical connectivity and directionality. **a** Bright field image of MO and StrO cultured onto an 8 × 8 electrode MEA chip. Individual electrode location is identified by line and column numbered between 1 and 8. The microfluidic system laying on top of the MEA is schematized in the lateral inset. **b** Raster plot of individual spike events detected during a representative 3 min recording for each active electrode. **c** Given two electrodes (e1 and e2), the Spike Time Tiling Coefficient (STTC) is calculated as the probability to detect at least one spike (*red* coloured) in e2 within a temporal window (Δt) centered around a given spike in e1. **d** Cross-correlograms of three pairs of electrodes (A-B, C-D, E-F) of a selected assembloid. When significant, the cross-correlogram maximum can be interpreted as the (most probable) time delay *t* required by the signal to propagate between the two electrodes. The signal speed *V* can be estimated as the ratio of the distance *r* between the two electrodes and the time delay *t*. **e** The mean signal directionality for a given electrode can be expressed as a vector. In this representative experiment, only electrode vectors (*black* arrows) having STCC higher than 0.8 are shown, together with the mean firing rate (in Hz) for the whole MEA. **f** Eleven assembloids (92%) were found to form a stable connection between MO and StrO within 40 days in vitro (D40). Four assembloids (33%) formed connections already by D4. **g** The mean electrical signal directionality of the assembloid can be computed as a vector with vertical (y) and lateral (x) components. In the 12 assembloids analyzed at different maturation stages, the positive sign of the mean vertical component (± standard deviation, STD) indicates that the electrical signal travels more from the MO to the StrO than the opposite direction. Statistical analysis was performed by Kruskal–Wallis post hoc analysis between all groups ** *p* < 0.01. **h** The mean lateral directionality (calculated as the absolute value of x ± STD) yielded a significant increase during the assembloid maturation (*** *p* < 0.001).

To investigate the functionality and directionality of this two-way connectivity, 12 assembloids were simultaneously recorded by a 768-channel microelectrode array (MEA) device. A microfluidic system laying on top of the MEA was designed to allow physical connection between the MO and the StrO through microtunnels (10 µm width) where neurites can grow in both directions and their activity is recorded by an 8 × 8 array of electrodes (Fig. 4a). The activity of the assembloids was monitored between D4 and D128 (Fig. 4b, an example of 3 min recording). The degree of electrical connectivity and signal directionality between different regions of the assembloid was assessed between each pair of electrodes. The Spike Time Tiling Coefficient (STTC, Fig. 4c) provided an estimate of the level of synchronisation, whereas the cross-correlogram was useful to infer the signal directionality and speed (Fig. 4d and Materials and Methods). The speed computed for the signal travelling between three representative electrode pairs was around 0.2–0.5 m/s, in agreement with a similar measurement performed in cultured rat cortical neurons^31^. The mean signal directionality for a given electrode A was represented as a vector computed from the MEA electrodes having significant STTC and cross-correlation with A (Fig. 4e). The mean signal directionality of the assembloid electrical activity was computed as the vectorial sum of these vectors. As a general criterion, if the vertical component (y) of this resulting vector reached a length bigger than the distance between 2 electrodes, the connection between the MO and the StrO was considered as established. This condition was satisfied by the 92% of the tested assembloids (Fig. 4f). The positive sign of the mean vertical component indicates that the electrical signal travels more from the MO to the StrO than the opposite direction (Fig. 4g). While the vertical component is already present at early stages of the assembloid, the lateral component x (Fig. 4h) progresses through the maturation stages, suggesting an increase in connectivity and synchrony between different regions of the MEA.

### Doxycycline inducible Progerin overexpression in the assembloid system

For the induction of aging phenotypes in the assembloid model, we used an iPSC line that was genetically engineered with a transgene containing the modified *LMNA* gene for the transcription of Progerin under the control of the Tet-On system, where Progerin is co-expressed with the GFP fluorescent protein in the presence of doxycycline. This line was used to generate assembloids (Supplementary Fig. 12a), and optimal concentration of doxycycline supplementation was determined by testing three different concentrations (1, 2 and 4 ng/μl). With flow cytometry we measured approximately 50% GFP-positive cells in D30 assembloids treated with the highest concentration of doxycycline, which was significantly higher than the other conditions (Supplementary Figs. 12b, c and 19 for the gating strategy). Similarly, Progerin levels validated by Western blot using the LMNA antibody (Supplementary Table 2), were significantly higher in the highest concentration (Supplementary Fig. 12d). The viability of assembloids was assessed by ATP and LDH assays (Supplementary Fig. 13), using control assembloids generated from the isogenic line (without the Progerin transgene, referred to as WT). We show significantly higher levels of LDH in the 4 ng/μl doxycycline treated WT assembloids which indicates that doxycycline could cause cytotoxicity in the model. However, no significant differences were found between the untreated and the 4 ng/μl doxycycline treated Progerin assembloids (Supplementary Fig. 13a). In the ATP assay, no differences were detected indicating no changes in the viability and metabolic activity of the doxycycline treated model (Supplementary Fig. 13b).

### Progerin-overexpressing assembloids show aging characteristics

**Fig. 5:**
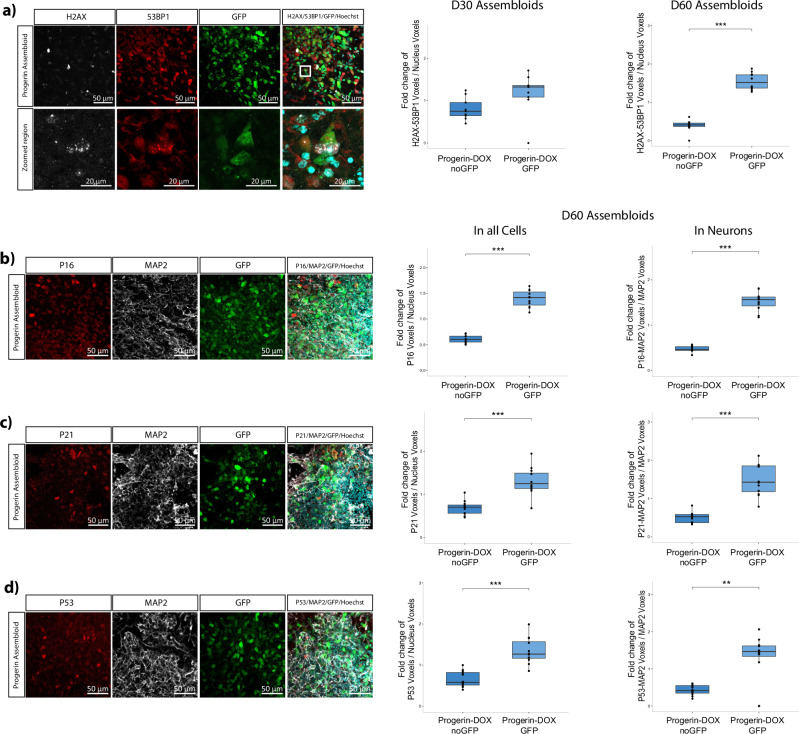
Progerin-overexpressing cells with aging characteristics in the assembloid model. **a** Immunofluorescence staining quantification of the H2AX and 53BP1 positive nuclear foci voxels normalised to the total nucleus voxels in 70 μm Progerin-overexpressing assembloid sections from D30 and D60 cultures. For D30 data, Welch’s *t*-test was performed with *n* = 9 for both conditions where each point represents one section per assembloid per batch for 3 batches. For D60 data two-sided Wilcoxon test was performed with *n* = 12 for both conditions where each point represents one section per assembloid per batch for 4 batches. *p < 0.05, **p < 0.01, ***p < 0.001. **b** Immunofluorescence staining quantification of the P16 voxels normalised to the total nucleus voxels, and P16 and MAP2 double positive voxels normalised to the total MAP2 voxels in 70 μm Progerin-overexpressing assembloid sections from D60 cultures. Two-sided Wilcoxon test for both plots was performed, with *n* = 11 where each point represents one section per assembloid per batch for 4 batches. *p < 0.05, **p < 0.01, ***p < 0.001. **c** Immunofluorescence staining quantification of the P21 voxels normalised to the total nucleus voxels, and P21 and MAP2 double positive voxels normalised to the total MAP2 voxels in 70 μm Progerin-overexpressing assembloid sections from D60 cultures. Welch’s *t*-test and two-sided Wilcoxon test was performed respectively, with *n* = 12 for both conditions where each point represents one section per assembloid per batch for 4 batches. *p < 0.05, **p < 0.01, ***p < 0.001. **d** Immunofluorescence staining quantification of the P53 voxels normalised to the total nucleus voxels, and P53 and MAP2 double positive voxels normalised to the total MAP2 voxels in 70 μm Progerin-overexpressing assembloid sections from D60 cultures. Welch’s *t*-test and two-sided Wilcoxon test was performed respectively, with *n* = 12 for both conditions where each point represents one section per assembloid per batch for 4 batches. *p < 0.05, **p < 0.01, ***p < 0.001. In all plots batch correction was applied by normalising each value to the mean of the values for each batch. Outlier removal was performed based on the Inter-Quartile Range (IQR) proximity rule. Data were plotted in R 4.2.2.

To determine whether Progerin-expressing cells in assembloids acquire aging characteristics, we stained D30 and D60 assembloid treated with 4 ng/μl doxycycline for aging-associated markers (Fig. 5). We were able to see that D60 assembloids had significantly higher levels of H2AX/53BP1 positive foci in the Progerin-expressing cells (marked as GFP+) compared to the non-Progerin-expressing cells (Fig. 5a). Similarly, p21, p16 and p53 aging-associated markers were all significantly elevated in the Progerin-expressing cells and neurons (MAP2/GFP-double positive) in D60 assembloids (Fig. 5b–d).

**Fig. 6:**
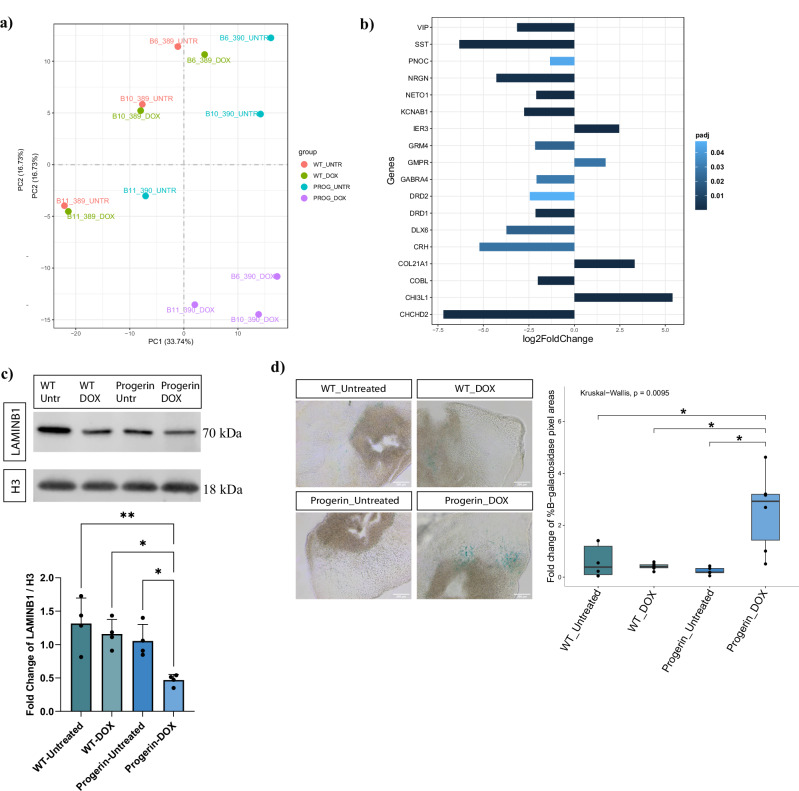
Evident aging phenotype in the Progerin-overexpressing assembloid model. **a** PCA plot of the two first principal components on the gene expression value (FPKM) of all samples. Each sample represents data from 4 pooled assembloids from one batch. **b** Plot showing the log2 fold change of significant differentially expressed genes between Progerin-overexpressing assembloids (PROG_DOX) and control (WT_UNTR, WT_DOX, PROG_UNTR) samples. This list of genes was extracted after the comparison of the assembloid data with post mortem human brain data^32,33^. **c** Western blot showing the protein levels of LAMIN B1 normalised to H3 housekeeping protein and batch corrected by normalising to the mean of the values for each batch. Outliers were calculated in GraphPad Prism using the ROUT method Q 1%. One-way ANOVA, with Tukey’s multiple comparison test was performed. For all conditions *n* = 4 with each point representing 3–4 pooled assembloids per batch, for 4 batches. Error bars represent mean ± SD. Data were plotted in GraphPad Prism 9.0.0. *p < 0.05, **p < 0.01, ***p < 0.001. **d** β-galactosidase staining for all the different assembloid conditions. Positive β-galactosidase areas were measured with ImageJ and normalised to the area of the section in each image and batch corrected by normalising to the mean of the values for each batch. Kruskal–Wallis test with Benjamini–Hochberg correction and Dunn’s multiple comparison test was performed. For all conditions *n* = 6 with each point representing one section per assembloid, per batch, for 3 batches. *p < 0.05, **p < 0.01, ***p < 0.001. Batch correction was applied by normalising each value to the mean of the values for each batch. Outlier removal was performed based on the Inter-Quartile Range (IQR) proximity rule. Data were plotted in R 4.2.2.

Next, we investigated the impact of Progerin-expressing cells on assembloids through bulk RNA sequencing transcriptomic analysis. Assembloids were generated from the Progerin-expressing cell line and its isogenic control under doxycycline treated (WT_DOX, PROG_DOX) and untreated conditions (WT_UNTR, PROG_UNTR). Principal component analysis (PCA) revealed distinct clustering of Progerin-expressing samples (Fig. 6a) and the heatmap of DEGs further highlighted a unique expression pattern (Supplementary Fig. 14a). Notably, the number of DEGs of Progerin-overexpressing assembloids were two or threefold higher than in control groups (Supplementary Fig. 14b). Transcription alterations in Progerin-overexpressing assembloids overlap with the observed DEGs in aged human post-mortem data from two studies^32,33^. This also included the expression of *PNOC* and *GMPR* genes (Fig. 6b), whose expression pattern is best associated with the aging brain, according to González–Velasco and colleagues^33^. These observations were made by comparing the significant DEGs (adj. *p* value < 0.05) that are commonly up or downregulated in the post-mortem and assembloid datasets (Supplementary Data 1–4). From these genes we focused on those with a log2FoldChange higher than 2, including *PNOC* (foldchange = −1.34) and *GMPR* (foldchange = 1.72) for the reason mentioned above. Furthermore, we were also able to observe significant reduction of the LAMIN B1 protein levels, an aging-related phenotype, in the Progerin-overexpressing assembloids (Fig. 6c). Finally, the β-galactosidase staining on assembloid sections revealed significantly higher levels of senescent cells in the Progerin-overexpressing assembloids (Fig. 6d). Overall, we consider that these results reveal a successful induction of aging in the Progerin-overexpressing assembloid model.

Finally, to assess whether pre-existing senescent cell populations exist in the assembloid model, we analyzed the expression of aging-related genes across various clusters, focusing on those identified in the transcriptome of Progerin-overexpressing assembloids. Most of these genes exhibit low expression across different cell types, suggesting that D30 assembloids used in the single nuclei RNA sequencing analysis are still in an early embryonic stage (Supplementary Fig. 15). It is also important to note that the data from the Progerin-overexpressing model were obtained from D60 assembloids. Therefore, the combination of a more advanced stage of differentiation along with progerin overexpression are likely responsible for the observed senescent and aging-related phenotypes in this model.

### Neurodegeneration phenotypes in the Progerin-inducible aged assembloids

**Fig. 7:**
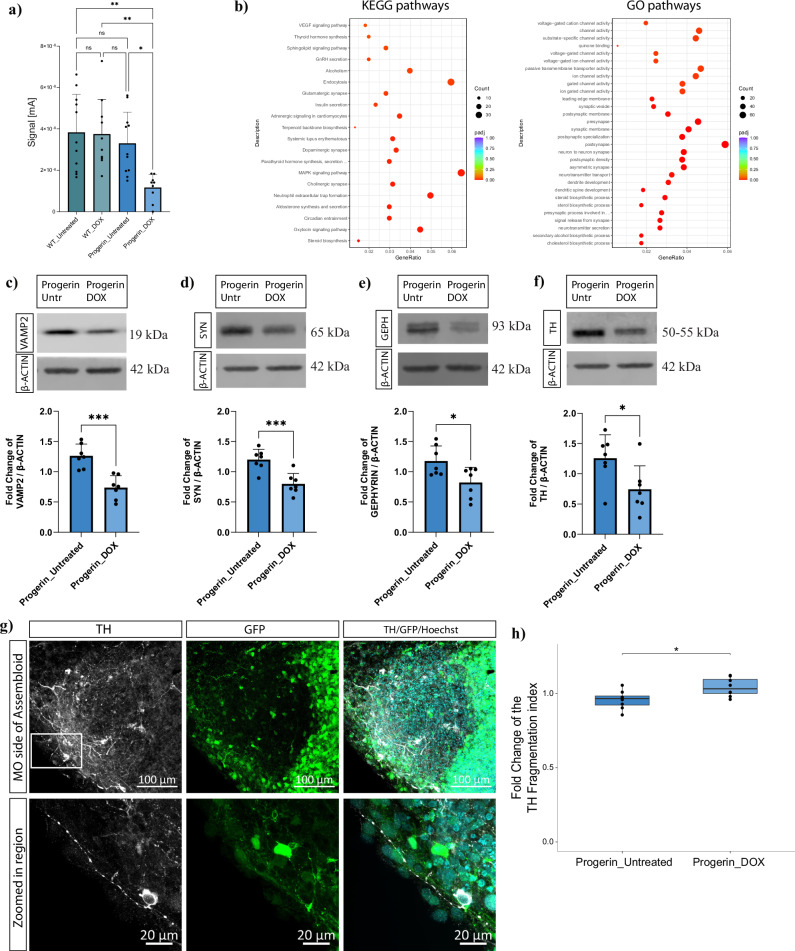
Early neurodegeneration phenotypes in Progerin-overexpressing assembloids. **a** Bar blot showing the electrochemical measurements in the StrO side of assembloids from the different conditions at D60. One-way ANOVA with Tukey’s multiple comparison test was performed. For all conditions *n* = the mean of measurements from 5 different positions in 3–4 assembloids per batch for 3 batches (WT_Untreated *n* = 11, WT_DOX *n* = 10, Progerin_Untreated *n* = 11, Progerin_DOX *n* = 9). Error bars represent mean ± SD. **b** KEGG and GO pathway enrichment analysis of the DEGs between PROG-DOX and PROG_UNTR samples. **c** Western blot for the protein levels of VAMP2 normalised to β-Actin. **d** Western blot for the protein levels of Synaptotagmin1 (SYN) normalised to β-Actin. **e** Western blot for the protein levels of Gephyrin normalised to β-Actin. **f** Western blot for the protein levels of TH normalised to β-Actin. **g** Representative confocal image of the MO side of a 70 μm Progerin-overexpressing assembloid section with TH and Hoechst immunostaining. The white square indicates the zoomed in region showing a representative image of a fragmented TH+ neurite. **h** Plot showing the TH fragmentation index as quantified by our neuronal skeleton quantification approach with MATLAB. Welch’s *t*-test was performed with *n* = 8 for each condition, where each point represents the average of 3–5 sections per assembloid per batch, for 4 batches. In all plots batch correction was applied by normalising each value to the mean of the values for each batch. *p < 0.05, **p < 0.01, ***p < 0.001. For (**c**–**f**) plots, Welch’s *t*-test was performed in each plot with *n* = 7 for each condition, where each point represents 3–4 pooled assembloid per batch, for 7 batches. Outliers were calculated in GraphPad Prism 9.0.0 using the ROUT method Q 1%. Error bars represent mean ± SD. For plot **h**, data were plotted in R 4.2.2 and outlier removal was performed based on the Inter-Quartile Range (IQR) proximity rule.

Next, we investigated whether the Progerin-overexpressing aged assembloids, at D60 of culture, lead to PD-relevant neurodegeneration phenotypes. First, we measured the catecholamine levels in the StrO tissue in assembloids from the control and aged conditions (Fig. 7a). Catecholamine levels were significantly lower in the striatum of the aged assembloid model. These data, along with the RNA sequencing analysis showing the clustering of all the control samples (Fig. 6a), demonstrates a similar phenotype between the three control conditions. Therefore, in the following experiments we compared assembloids generated from the Progerin line without doxycycline treatment (Progerin_Untreated) with assembloids from the same line with doxycycline supplementation (Progerin_DOX). KEGG and GO enrichment analysis from the transcriptomic data revealed significant dysregulation of synaptic and DA transmission related pathways (Fig. 7b), with the majority of the responsible genes being downregulated in the Progerin-overexpressing assembloids (Supplementary Fig. 16a–e). To confirm these results at the protein level, we observed significant downregulation of the postsynaptic protein Gephyrin and the presynaptic proteins VAMP2 and Synaptotagmin1 (SYN) in Progerin-overexpressing assembloids (Fig. 7c–e).

Loss of TH-positive DANs, which leads to striatal DA depletion is the key characteristic of PD^4^. The lower catecholamine levels detected in the striatal compartment of aged assembloids, are a first indication that this pathology hallmark is recapitulated in the aging model. To further substantiate this finding, we assessed the levels of TH protein. Consistent with the electrochemical measurements of dopamine, the TH protein levels are also reduced upon induced aging (Fig. 7f). Finally, we investigated the TH neuronal skeleton integrity using our automated image analysis method^34^. This calculation suggests a possible increase in the fragmentation of TH-positive neurites, which is an early sign of degeneration (Fig. 7g, h). Altogether, these data suggest an early loss of DANs’ function in induced aged assembloids that mimics early stages of PD pathology.

## Discussion

To date, most studies in PD research with advanced cellular 3D models are based on the use of midbrain organoids^35^. Although these organoids have proven to be an excellent tool for studying the DANs’ vulnerability in different PD-related conditions, they are not able to recapitulate the dysregulated connectivity in the nigrostriatal pathway, which is crucially affected in PD^36^. For the reconstruction of the nigrostriatal pathway connectivity, here we show the generation of a midbrain-striatum assembloid model that retains the identity of the two regions with the expression of midbrain- and striatum-specific markers, respectively.

Single nuclei RNA sequencing analysis further revealed the identity specificity of MOs and StrOs, while the assembloid model demonstrated the preservation of cellular populations from both organoids, with the additional identification of A9 and A10 DANs clusters that are not clearly detected in our MOs dataset. A9 DANs were revealed by the high expression of *KCNJ6* that encodes GIRK2 protein^37^, while expression of *OTX2*, a crucial TF for the specification of the A10 DANs^38^, was found only in the A10 cluster. In parallel, the MSN population in the assembloid model is specified by the co-expression of *ARPP21* and *PPP1R1B*, a strong indication of an MSN identity^39,40^, while in the StrOs dataset only *ARPP21* expression was found. This illustrates that the assembloid model, probably through functional communication between both regions, favours the further specification of the midbrain DANs as well as the MSNs of the striatum.

Enrichment analysis of the DEGs between the assembloids and individual organoids revealed the upregulation of genes related to developmental neurogenesis, axonal guidance, and neuronal maturity. In line with the importance of brain’s interregional communication in neuronal maturity and functionality^41^, it is possible that neurogenesis and neuronal maturity are promoted by the interactions formed between midbrain and striatal neurons in the assembloid model. These observations align with another study on cortico-thalamic assembloids, where neuronal maturity was observed at the assembloid level compared to individual organoids^42^. Moreover, this assumption is further supported by the reduced expression of cellular and oxidative stress-related genes in midbrain-striatum assembloids, as elevated stress levels in cerebral organoids have been linked with impaired cellular maturity^28^.

Nigrostriatal pathway connectivity and functionality are also confirmed in assembloids, with the formation of active synapses between midbrain and striatal neurons and the release of catecholamines from the midbrain to striatum. This is particularly important, as the catecholamine DA release in the dorsal striatum from the SNpc DANs’ axons is the major functionality of the nigrostriatal pathway^43^. The communication between the midbrain and striatum organoids was further assessed by microelectrode array, showing that assembloids can resemble the nigrostriatal connectivity, with the striatum organoids receiving electrochemical input from the midbrain.

A limitation of iPSC-derived organoid models in the research of age-related neurodegenerative diseases is their lack of aging-specific phenotypes^44^. Since aging is the major risk factor in PD, we aimed to model this aspect in midbrain-striatum assembloids. To achieve this, we leveraged the overexpression of Progerin. A similar approach has been previously described, where transient Progerin overexpression in iPSC-derived DANs resulted in aging and neurodegeneration phenotypes^45^. In our mosaic Progerin-expressing model, we found that Progerin-overexpressing cells acquire increased levels of DNA double strand breaks marked with the double positive H2AX-53BP1 nucleus foci^46^. Moreover, Progerin-overexpressing cells and neurons had significantly higher levels of p21^CIP1^, p53 and p16^INK4A^. The upregulation of these three proteins has been shown to be associated with cellular senescence and aging^47^. Similar aging hallmarks were observed in Progerin-overexpressing cortical organoids.

An aging-related transcriptomic profile was observed in Progerin-overexpressing assembloids, with the identification of aging-related genes with a common expression pattern between assembloids and human brain post-mortem data^32,33^. Many of the noteworthy upregulated and downregulated genes are associated with neurodegeneration and aging brain according to the literature (Supplementary Table 6), highlighting the interconnected relationship between aging and neurodegeneration. Particularly, *IER3* downregulation is also observed in the Progerin-overexpressing cortical organoids, indicating a possible crucial role in brain aging induction.

Synaptic dysfunction in the aging brain is one of the major promoters of neurodegeneration^48^. Dysregulation of synaptic and DA neurotransmission pathways, as revealed in the transcriptomic data of Progerin-overexpressing assembloids, was further validated by the downregulation of the post-synaptic protein Gephyrin, an important scaffolding protein that regulates the organisation of post-synapses in striatal GABAergic neurons^49^, and the pre-synaptic proteins VAMP2 and Synaptotagmin1. VAMP2 regulates the fusion of synaptic vesicles and neurotransmitter release through the SNARE complex^50^ and Synaptotagmin1 is an essential Ca^2+^ sensor for the fast release of DA^51^. In parallel, electrochemical measurements showed significantly lower catecholamine levels in the striatum of the Progerin-overexpressing assembloids, indicating dysregulation of DA release in the aged-induced model, a PD-relevant phenotype^52^. These results reveal a defective synaptic and DA release system in Progerin-overexpressing assembloids.

Importantly, aging is a complex phenomenon, including multiple processes. When we here describe “aging-induced” or “aged-induced”, this description is only limited to the specific features that are investigated here. It is neither including nor excluding that other, ageing associated processes are also affected.

Moreover, low TH protein levels in Progerin-overexpressing assembloids could be linked to dysregulated DA synthesis and the presence of vulnerable DANs. TH is the key enzyme in DA synthesis^53^. Lower TH protein levels have been previously observed in surviving DANs of PD patients, indicating their vulnerability to the disease. Moreover, loss of TH activity followed by TH protein decline has been linked to DA deficiency in PD^54^. Additionally, the presence of possibly more fragmented TH neurites in progerin-overexpressing assembloids, demonstrates a degeneration phenotype prior to neuronal death^55^. The axonal fragmented phenotype has been previously described in iPSC-derived PD neuronal models^56^. These results support that our age-induced assembloids display an early PD-relevant neurodegeneration phenotype, with dysregulation in the DA synthesis and release systems indicative of axonal degeneration preceding the eventual loss of DANs’ soma, in retrograde fashion as typically observed in PD^57^.

The here presented age-induced midbrain-striatum assembloid model offers new possibilities for PD research. By incorporating aging characteristics in cellular models derived from PD patient cell lines, it is possible to recapitulate more accurately the disease state of patients and unveil cellular dysfunctionalities that remain unnoticed in the current developmental models. This approach will provide further insights into the model’s robustness and its potential applications in personalised medicine.

## Methods

### iPSCs and NESCs

The iPSCs that were used in this study are described in Supplementary Table 1. The iPSCs were cultured in 6-well plates (Thermo Fisher Scientific, 140675) coated with Geltrex (Life Technologies, A1413302). For the first 24 h the cells were cultured in Essential 8 Basal medium (Thermo Fisher Scientific, A1517001) supplemented with 1% Penicillin/Streptomycin (Invitrogen, 15140122) and 10 µM ROCK Inhibitor (Ri) (Y-27632, Millipore, SCM075). After the 24 h, the cells were cultured in Essential 8 Basal Medium, with daily media changes. Confluent iPSCs (~70–90%) were split using UltraPure™ 0.5 M EDTA, pH 8.0 (Thermo Fisher Scientific, 15575020). Immunofluorescence staining was performed to confirm the pluripotent identity of the iPSCs (Supplementary Fig. 1a–d). NESCs were generated from iPSCs using a previously described protocol^58^. Briefly, NESCs were derived from iPSCs through embryoid body formation and neuroepithelial expansion, facilitated by the small molecules CHIR99021 (CHIR, 3 μM) and purmorphamine (PMA, 0.5 μM). The NESCs were maintained in N2B27 medium on 6-well plates (Thermo Scientific) pre-coated with Geltrex. The N2B27 medium consisted of a 1:1 mixture of DMEM-F12 (Invitrogen) and Neurobasal (Invitrogen), supplemented with 1:200 N2 (Invitrogen), 1:100 B-27 without vitamin A (Invitrogen), 1% Glutamax (Thermo Fisher), and 1% penicillin/streptomycin (Invitrogen). For maintenance, the medium was freshly supplemented with 3 μM CHIR (Axon Medchem), 0.75 μM PMA (Enzo Life Science), and 150 μM ascorbic acid (Sigma). Media changes occurred every other day, and cells were routinely passaged at 80–90% confluence using Accutase (Sigma). Cells were cultured at 37 °C with 5% CO_2_. The NESCs’ neural stem cell identity was confirmed with immunofluorescence staining (Supplementary Fig. 1e–g).

### Striatum organoids

Striatum organoids (StrOs) were generated using an adapted protocol (C4) from^15^ (C3) after comparing it with two other conditions (RA and SR) in two time points of culture (day (D)35 and D50). iPSCs at ~70% confluency were used for the StrOs generation. Before the procedure of spheroids formation, the cells were treated overnight with 1% DMSO (Sigma-Aldrich, D2650) in Essential 8 basal medium. For the spheroids formation (D -1), the iPSCs were first incubated into Accutase at 37 °C for 5 min. The accutase was stopped using 5× DMEM-F12 (Thermo Fisher Scientific, 21331-046). The cells were then resuspended in Essential 8 medium containing 20 μM Ri and counted using the Countless cell counting chambers slides (Invitrogen, C10313). For condition C4, 10,000 cells were added per well in the BIOFLOAT™ 96-well plate U-bottom (faCellitate, F202003), centrifuged in 100 × *g* for 3 min and then incubated in the normal culture conditions of 37 °C with 5% CO_2_. The spheroids were left intact for two days to form properly and at D1 the medium was exchanged with Essential 6 medium (Thermo Fisher Scientific, A1516401) supplemented with 10 μM Ri, 2.5 μM Dorsomorphin (Sigma-Aldrich, P5499) and 10 μM SB-431542 (Abcam, ab120163). The spheroids were cultured in the same medium until D5, with a reduction of the Ri concentration to 10 μΜ on D2, 5 μΜ at D3 until completely removed at D4. To start the differentiation process of the spheroids into StrOs, on D6 the medium was exchanged with a medium containing Neurobasal-A (Thermo Fisher Scientific, 10888022), 2% B-27 without Vitamin A (Thermo Fisher Scientific, 12587010), 1% Penicillin/Streptomycin (Invitrogen, 15140122), 1% GlutaMAX (Thermo Fisher Scientific, 35050061) and supplemented with 2.5 μM IWP-2 (Selleckchem, S7085) and 50 ng/ml Activin A (Thermo Fisher Scientific, PHC9561). From D9 to 17, the media was additionally supplemented with 100 nM SR11237 (Tocris, 3411). Until D17 the medium was exchanged daily. From D17 to 35, the media was changed to promote the neuronal differentiation and it was supplemented with 20 ng/ml BDNF (PeproTech, 450-02), 20 ng/ml NT-3 (Alomone labs, N-260), 200 μM AA (Sigma-Aldrich, A4544) and 100 μM cAMP (Biosynth, ND07996), with medium exchanges every 3 to 4 days. For condition C3, the same protocol as described in Miura and colleagues^15^ was followed, without the addition of cis-4,7,10,13,16,1 9-docosahexaenoic acid (DHA) at D22 of differentiation.

For the generation of StrOs from the RA and SR conditions, iPSCs at ~70% confluency were collected using accutase as described above, and 9000 cells were plated in each well of the BIOFLOAT™ 96-well plate U-bottom (D -2). The media used for plating had 80% DMEM F12 (Thermo Fisher Scientific, 21331046), 20% KOSR (Thermo Fisher Scientific, 10828028), 3% FBS (Invitrogen, 16140071), 1% GlutaMAX, 1% NEAA (Thermo Fisher Scientific, 11140-050) and 0.7% 2-Mercaptoethanol 50 mM (Thermo Fisher Scientific, 31350-010). This media was supplemented with 10 μM Ri and 40 ng/ml FGF-basic (PeproTech, 100-18B). After two days (D0 of culture) the media was exchanged with the Neural induction medium (NIM) containing 76.8% DMEM F12, 20% KOSR, 1% NEAA, 1% GlutaMAX, 1% Penicillin/Streptomycin and 0.2% 2-Mercaptoethanol 50 mM. From D0 to 2 the NIM was supplemented with 5 μΜ DM, 10 μM SB and 10 μM Ri, while on D2 Ri was removed. From D2 to D16 the medium was exchanged daily. On D4 and 5 the medium was additionally supplemented with 5 μΜ IWP-2. On D6, NIM was exchanged to the neural differentiation medium (NM) which contained 96% Neurobasal A medium, 2% B-27 without Vitamin A, 1% GlutaMAX and 1% Penicillin/Streptomycin. From D6 to 8, NM was supplemented with 20 ng/ml FGF-basic, 20 ng/ml EGF (PeproTech, AF-100-15) and 5 μΜ IWP-2. From D8 to 16, NM was supplemented with 20 ng/ml FGF-basic, 20 ng/ml EGF, 5 μΜ IWP-2, 50 nM SAG (Merck Millipore, 566660), 50 ng/ml Activin A and 1 mM for condition RA or 100 nM SR11237 for condition SR. On D17 the media was exchanged with a non-supplemented NM. From D18 to D35 or D50, NM was supplemented with 20 ng/ml BDNF and 20 ng/ml NT3, with media changes every 3 to 4 days.

### Midbrain organoids

MOs were generated using NESCs as the starting population of cells. The protocol that was used is a slightly altered version of the one described in Monzel and colleagues^7^. At D0, 9000 NESCs were added per well in the BIOFLOAT™ 96-well plate U-bottom and cultured in maintenance medium for 2 days. At D3 the medium was exchanged with the differentiation medium containing 1 µM purmorphamine and at D8 with the final differentiation medium without purmorphamine. The organoids were kept in static conditions and in the 96-well plates U-bottom, until used for the assembloids generation (described below).

### Assembloids

Assembloids of midbrain and striatum organoids were generated using MOs at D20 and striatum organoids at D35 of culture. Due to the different media composition of the organoids, an optimisation of the co-culture medium was needed. Four different media were tested. The Neural Medium (NM) was comprised of Neurobasal-A, 2% B-27 without Vitamin A, 1% Penicillin/Streptomycin and 1% GlutaMAX, while the Neural Medium Plus (NMpl) was supplemented with B-27 plus (Thermo Fisher Scientific, A3582801) instead of the B-27 without Vitamin A. In the third condition, the Neural Medium++ (NM++) was tested, which was the NM medium supplemented with 20 ng/ml BDNF (PeproTech, 450-02), 10 ng/ml GDNF (PeproTech, 450-10), 20 ng/ml NT-3 (Alomone labs, N-260), 200 μM AA (Sigma-Aldrich, A4544) and 100 μM cAMP (Biosynth, ND07996). The optimal assembloid co-culture condition for the assembloid model was consisted of the N2B27 medium (described by Monzel and colleagues^7^), which consists of DMEM F12 (Invitrogen)/Neurobasal (Invitrogen) 50:50 with 0.5% N2 supplement (Thermo Fisher Scientific, 17502001), 1% B-27 without Vitamin A, 1% GlutaMAX and 1% Penicillin/Streptomycin. The media was further supplemented with 20 ng/ml BDNF (PeproTech, 450-02), 10 ng/ml GDNF (PeproTech, 450-10), 20 ng/ml NT-3 (Alomone labs, N-260), 200 μM AA (Sigma-Aldrich, A4544) and 100 μM cAMP (Biosynth, ND07996). At D0 of the assembloids generation, each StrO was transferred in each well of the 96-well plate U-bottom that contained the MOs and the medium was exchanged to the assembloid co-culture medium. After 4 days, the two organoids were merged into an assembloid, and they were transferred in 24 well ultra-low attachment plates (Celltreat, 229524). Some of the assembloids were embedded in 30 µl Geltrex (Invitrogen, A1413302), as described before^7^. The assembloids were cultured at 37 °C, 5% CO_2_ under static conditions.

For the Progerin overexpression induction, assembloids that were generated from the Progerin-overexpressing cell line were treated with 4 ng/μl doxycycline (Sigma-Aldrich, D9891). Generation and validation of iPSC line expressing Progerin under control of the Tet-ON system is described elsewhere.

### ATP and LDH assay

Intracellular ATP in assembloids was measured using luminescence based CellTiter-Glo® 3D Cell Viability Assay (Promega, G9681). Three organoids per cell line and per condition were transferred each in one well of the imaging plate (PerkinElmer, 6055300). Fifty microliters of CellTiter-Glo® reagent were added to each well and the plate was incubated for 30 min on a shaker at room temperature (RT). Luminescence was measured using Cytation5 M cell imaging reader (RRID:SCR_019732). The experiment was repeated for three independent derivations (batches) at D30 assembloids. The mean signal of three assembloids of each cell line and condition was calculated and normalised to the mean size (area) of the assembloids. Brightfield images of random assembloids of the three batches taken with the ZEISS Axio Vert.A1 + Axiocam ICM1 microscope and the area of each assembloid was calculated with the ZEN (blue edition) software (RRID:SCR_013672). The mean area of all assembloids measured per line and conditions from each batch was used for the normalisation.

For determining the cytotoxicity in the assembloids, the LDH-Glo™ Cytotoxicity Assay (Promega, J2381) was used. In the day of assembloids collections (D30 of culture) media from three assembloids per line and condition from three batches was collected and snap frozen in liquid nitrogen. For the LDH assay, the snap frozen media was thawed on ice. Fifty microliters of media from each sample and 50 μl of the enzyme and substrate mix was pipetted in a well of the imaging plate (PerkinElmer, 6055300). The plate was briefly mixed and incubated for 1 h at RT avoiding exposure to light. Luminescence was measured using Cytation5 M cell imaging reader. Similar to the ATP assay, the mean signal of the assembloids was normalised to the mean of the assembloids’ area.

### Western blotting – RIPA buffer

For Western blotting, four non-embedded assembloids or six to eight organoids were lysed using RIPA buffer (Abcam, ab156034) supplemented with cOmplete^TM^ Protease Inhibitor Cocktail (Roche, 11697498001) and Phosphatase Inhibitor Cocktail Set V (Merck Millipore, 524629). The samples were pipetted 10–20 times up and down until dissolved and were incubated on ice for 20 min. For DNA disruption, lysates were sonicated for 10 cycles (30 s on / 30 s off) using the Bioruptor Pico (Diagenode), followed by centrifugation at 4 °C for 20 min at 14,000 × *g*. The protein concentration was measured using the Pierce™ BCA Protein Assay Kit (Thermo Fisher Scientific, 23225). Samples were adjusted to the same concentration by appropriate dilution with RIPA buffer and boiled at 95 °C for 5 min in denaturing loading buffer. 2.5–10 μg of protein was loaded per sample for every Western blot. Protein separation was achieved using SDS polyacrylamide gel electrophoresis (Bolt™ 4–12% Bis-Tris Plus Gel, Thermo Fisher Scientific) and transferred onto a PVDF membrane using iBlot™ 2 Gel Transfer Device (Thermo Fisher Scientific). After transfer the membrane was dried for 10 min at 37 °C and subsequently activated with 100% Methanol for 30 s. The membranes were washed twice with PBS containing 0.02% Tween and they were blocked for 1 h at RT in 5% skimmed milk powder dissolved in PBS. After blocking, the membranes were washed quickly with PBS containing 0.02% Tween and were incubated overnight at 4 °C with the primary antibodies prepared in 5% BSA and 0.02% Tween in PBS (Supplementary Table 2). The next day, membranes were washed three times for 5 min with PBS containing 0.02% Tween and incubated with DyLight™ secondary antibodies at a dilution of 1:10,000 (anti-rabbit IgG (H + L) 800, Cell Signaling, 5151P or anti-mouse IgG (H + L) 680, Cell Signaling, 5470P) for 1 h. Membranes were revealed in the Odyssey® Fc 2800 Imaging System and exposure time was from 30 s to 4 min, depending on the primary antibody used. Western blots were analysed using ImageJ (RRID:SCR_003070) software.

### Western blotting – nuclear and cytoplasmic fractionation

The protocol described by Abcam (https://www.abcam.com/protocols/nuclear-extraction-protocol-nuclear-fractionation-protocol) was used for the nuclear-cytoplasmic fractionation. At the last step of the protocol both nuclear and cytoplasmic samples were sonicated for 10 cycles (30 s on / 30 s off) using the Bioruptor Pico (Diagenode). For the preparation of the nuclear extraction and fractionation buffers the reagents used were HEPES (Sigma-Aldrich, H3375), KCl (AppliChem, 8059), MgCl_2_ (Sigma-Aldrich, M8266), EDTA (Sigma-Aldrich, E9884), EGTA (Sigma-Aldrich, E3889), DTT (Thermo Fisher Scientific, R0861), cOmplete^TM^ Protease Inhibitor Cocktail and Phosphatase Inhibitor Cocktail Set V. The following Western blotting procedure is described in the previous section.

### Flow cytometry

Three embedded assembloids per condition were used for GFP+, live cells measurement in BD LSRFortessa flow cytometer (RRID:SCR_019601). Geltrex embedded assembloids were first incubated at 37 °C for 40–50 min on shaker in 500 μl of papain solution containing 20 ml DMEM-F12, 36 mg Papain (Sigma-Aldrich, P4762), 8 mg EDTA (Sigma-Aldrich, E6758) and 8 mg L-Cystein (Sigma-Aldrich, C6852). To start the dissociation process, papain solution was replaced with 500 μl accutase and the assembloids were pipetted with the 1000 pipette, followed by a 10 min incubation shaking. After that, pipetting with the 200 μl pipette and incubation cycles were continued until the complete dissociation of the assembloids. For accutase and papain inhibition, 500 μl papain inhibitor solution containing 5 mg/ml BSA (Carl Roth, 8076.4) and 5 mg/ml Trypsin inhibitor (Sigma-Aldrich/Roche, 10109878001) in PBS was added. After transferring the total volume in 2 ml Eppendorf tube, the dissociated assembloids were centrifuged at 500 × *g* for 5 min. Supernatant was discarded and the pellet was washed once with PBS. The pellet was resuspended in 300 μl DMEM (Thermo Fisher Scientific, A14430-01) containing 1:1000 concentration live-dead stain Zombie NIR (Biolegend, 423106), followed by incubation at 37 °C for up to 30 min. Cells were then centrifuged at 500 × *g* for 3 min and pellet was washed twice with PBS and centrifuged again with the same setting. After the final wash and centrifugation, the pellet was resuspended in DMEM. The samples were then run in Becton Dickinson LSRFortessa, with 10,000 events acquisition of GFP+, live-cells. Each sample was run in two technical replicates. The data were analysed using the FlowJo software (v.10.7.2, RRID:SCR_008520).

### Rabies virus based retrograde monosynaptic tracing

#### Lentiviral vector and rabies virus vector productions

The construct pBOB-synP-HTB [gift from Edward Callaway & Liqun Luo (Addgene plasmid # 30195 ; http://n2t.net/addgene:30195 ; RRID:Addgene_30195)]^59^ was used for the production of the replication-deficient LV-GP-TVA-GFP lentiviral vector. High-titer preparations of lentiviral particles were produced, as previously described^60^. The titer of the preparation used was 3 × 10^8^ IU/mL.

For the production of RBV-ΔG-EnvA-RFP rabies viral particles, we followed stages III-VI of the previously established protocol for the amplification, pseudotyping, and concentration of the virus^61^. Titer was 4 × 10^7^ IU/mL.

#### Lentiviral vector and rabies virus vector transduction of assembloids

To assess the connectivity through active synapses between the midbrain and striatum neurons in the assembloid model, striatum organoids at D35 of culture were transduced by adding concentrated LV-GP-TVA-GFP viral particles at 1:500 dilution, in the culture medium. After 7 days the medium containing the lentiviral vector was discarded and the organoids were washed twice with fresh medium. The LV-transduced StrOs were then merged with MOs and the assembloids were infected by the addition of the concentrated RBV-ΔG-EnvA-RFP rabies viral particles at 1:500 dilution in the culture medium. Control experiments, with single infection of assembloids with only one of the vectors at a time, were performed to confirm the specificity of the signal. After 7 days the media was changed and the assembloids were cultured for up to D30. Seventy micrometres-thick sections were either imaged directly for the observation of the RFP and GFP signal from the viral infections or were immunostained using a tyrosine hydroxylase (TH) antibody to assess the colocalization between TH and RFP.

#### β-galactosidase

Seventy micrometres sections from assembloids were used in the β-galactosidase staining with the Senescence Detection Kit (Abcam, ab65351). One section from two or three assembloids per condition from four batches were used. Images were taken using the colour camera setting with 4× objectives in the Olympus IX83 microscope (RRID:SCR_020344). β-galactosidase positive areas were quantified using ImageJ. Positive areas in each section were summed and normalised to the total area of the section in the image. The normalised value was multiplied by 100 for calculating the % of positive β-galactosidase areas in the image.

### Immunofluorescence staining

#### iPSCs and NESCs

The procedure that was used for the immunofluorescence staining characterisation of the iPSCs is detailed described by Gomez-Giro and colleagues^62^. iPSCs and NESCs were cultured on Geltrex-coated 96-well imaging plates (PerkinElmer, 6055300) until they reached approximately 70% confluency. Next, cells were fixed for 15 min at RT with 4% Paraformaldehyde (PFA), washed 3× for 5 min with PBS and permeabilized with 0.3% Triton X-100 in PBS for 15 min at RT. After permeabilization, cells were washed 3× for 5 min with PBS and then blocked with 10% fetal bovine serum (FBS) in PBS for 1 h at RT. Primary antibodies (Supplementary Table 3) were diluted in 3% FBS in PBS and the cells were incubated overnight at 4 °C. Then cells were washed 3× for 5 min with PBS and incubated for 1 h at RT with secondary antibodies (Supplementary Table 3) and Hoechst 33342 (Invitrogen, 62249). After 3 washes for 5 min with PBS, cells were kept in 0.1% Sodium Azide (NaAz) in PBS until imaging with confocal microscopy.

#### Organoid and assembloid sections

Assembloids and organoids were fixed with 4% PFA overnight at 4 °C, and then washed with PBS three times for 15 min at RT. At least three organoids/assembloids per line and time point were embedded in 3% low-melting point agarose (Biozym, 840100). 70 μm sections were obtained using the vibratome (Leica VT1000s, RRID:SCR_016495). The sections were permeabilized for 30 min in 0.5% Triton X-100 and blocked for 2 h with blocking buffer containing 2.5% normal goat serum, 2.5% BSA, 0.01% Triton X-100 and 0.1% sodium azide in PBS at RT. Sections were incubated with the primary antibodies (Supplementary Table 3) diluted in blocking buffer for 48–72 h at 4 °C. The sections were washed with 0.01% Triton X-100 for 5 min three times and then incubated with the secondary antibodies (Supplementary Table 3) and Hoechst at 1:1000 dilution for 2 h at RT. The sections were then washed again with 0.01% Triton X-100 for 5 min three times at RT. After the last wash, the sections were kept in MilliQ water and mounted on slides using Fluoromount-G mounting medium as described by Nickels and colleagues^19^.

#### Microscopy

For high-content image analysis, one 70 μm section from three organoids/assembloids of each condition from at least three batches was acquired using the Yokogawa CV8000 high-content screening microscope (RRID:SCR_023270) with a 20X/0.75 numerical aperture (NA) objective.

For qualitative analysis, images were acquired using a confocal laser scanning microscope (Zeiss LSM 710, RRID:SCR_018063) with the 20X/0.8 NA, 40X/1.3 NA or 63X/1.4 NA objective.

### Light sheet fluorescence expansion microscopy

#### Sample preparation

Following Rodriguez-Gatica and colleagues^63^ expansion protocol for organoids, whole-assembloids were prepared using a methylacrylic acid-NHS linker, washed, and incubated in a monomer solution. Gelling involved 4-hydroxy-TEMPO, TEMED, and ammonium persulfate, with samples placed on ice to prevent premature polymerisation. After gelling, samples underwent digestion, were washed, and stored in PBS, expanding to 1.5 times their original size. Autofluorescent protein preservation facilitated specific immunolabeling (e.g., DANs) and post-digestion nuclear staining with Hoechst 33342 at a concentration of 2.5 μg/ml.

#### Light sheet microscopy

Post-expansion, whole-assembloids were imaged using light sheet microscopy for meso- and microscopic scale analysis. The Blaze microscope (LaVision-Miltenyi Biotec) was used for whole-assembloid imaging, and a custom-built setup was employed for higher-resolution imaging of selected ROIs. Samples were affixed to coverslips with poly-L-lysine and placed in an imaging chamber filled with PBS solution. For mesoscopic imaging with the Blaze microscope, a single illumination arm with a light-sheet thickness of 6 µm and a LaVision-Miltenyi 4X/0.35 NA, WD 15 mm WI objective lens captured images in a mosaic pattern due to sample size. A 12× (LaVision-Miltenyi 12X/0.53 NA WD 8.5 mm WI) objective was used as an intermediate step for unclear projections. Microscopic imaging employed a high-resolution Nikon CFI75 25x/1.1 NA WI objective, achieving resolutions suitable for subcellular structural characterisation. Complete assembloid imaging at this resolution is often impractical due to data volume (about 500 GB per 1 mm³ per channel), as noted by Rodriguez-Gatica and colleagues^63^. Therefore, specific ROIs identified in the mesoscale data were selected for detailed microscopic analysis.

#### Data processing

3D image stacks were processed using MATLAB scripts and spatially deconvolved with Huygens software (Professional version 22.04, Scientific Volume Imaging, The Netherlands). Stitching multiple datasets for 3D representations was conducted in FIJI^64^ using a two-step approach to manage large data sizes. Visualisation was achieved with Imaris (Version 10.0.1, Bitplane Inc.). All processing was performed on a HIVE workstation equipped with dual Intel Xeon Gold 6252 CPUs and an Nvidia RTX A4000 GPU.

#### Image analysis

Images obtained from the Yokogawa microscope were processed and analyzed in MATLAB (2021a, Mathworks, RRID:SCR_001622) using a previously described image analysis pipeline^34,65^.

#### Electrochemical detection of catecholamines in neuronal tissues

Commercially available Nafion-coated carbon fiber microelectrodes (World Precision Instruments, CF10–50) were employed to measure the presence of catecholaminergic neurotransmitters (e.g., dopamine) within the assembloids. Amperometric measurements were performed according to the manufacturer’s instructions using a potentiostat (Bio-Logic, VMP3) equipped with a low current module (Bio-Logic) and a custom-made platform (Supplementary Fig. 11d). Electrodes were activated by applying a potential of 1.2 V and simultaneous exposure to a 150 mM NaCl solution with a pH of 9.5. All measurements were conducted at a potential of 0.65 V. Prior to the measurements, assembloids were washed three times using PBS. Assembloids were subsequently placed onto the custom-made platform, covered in 100 µl of PBS before electrodes were carefully inserted into the tissue using a laboratory jack. Measurements on StrOs alone and StrOs within D30 assembloids were conducted by introducing the electrode 1 and 2 times into the centre of the neuronal tissue. For the StrOs cultured in pre-used MOs media, the media was in contact with the MOs for 24 h. Then it was transferred into the wells containing StrOs. StrOs were cultured in the pre-used MO media for 48 h prior to the measurements. Due to the occasional occurrence of necrotic cores within assembloids electrochemical measurements on D60 assembloids were performed by introducing the electrodes at the border of the StrO. Measurements were conducted at five different locations within the StrO of the assembloid. To exclude any cross-contaminations, electrodes were carefully washed, and background signals were recorded in between the measurement of each assembloid. Measurement results were averaged and unless stated otherwise data were background subtracted.

#### Quantitative PCR

The RNeasy Mini Kit (Qiagen, 74106) was used for the total RNA extraction from striatum organoids. The RNA concentration was measured using the Nanodrop 2000c Spectrophotometer (Thermo Fisher Scientific, RRID:SCR_020309). The High-Capacity RNA-to-cDNA^TM^ Kit (Thermo Fisher Scientific, 4387406) was used for the cDNA synthesis. For the quantitative PCR reaction, the Maxima SYBR Green qPCR Master Mix (Thermo Fisher Scientific, K0221) was used with the primers listed in Supplementary Table 4. The Aria Mx Real-Time PCR system (Agilent) was used and the data were extracted from the AriaMx PC software. For each qPCR reaction, *n* = 3–5, where n is one sample of 6–8 pooled organoids, from 3–5 batches.

#### RNA sequencing

RNA was extracted from the assembloids using the RNeasy Mini Kit. Four assembloids were used per condition and from three batches. The RNA samples were shipped with dry ice to Novogene in UK for the RNA sequencing experiment and bioinformatic analysis.

In the process of obtaining clean reads, reads containing adapters, higher than 10% undetermined bases and low quality (Qscore of over 50% bases of the read is <=5) were removed. Poly-T oligo-attached magnetic beads were used to purify the messenger RNA from the total RNA. After fragmentation, the first strand cDNA was synthesised using random hexamer primers, followed by the second strand cDNA synthesis using dUTP for directional library. To quality control the library, Qubit and real time PCR were used for quantification, while for the size distribution detection bioanalyzer was used. The quantified libraries were combined and sequenced on Illumina platforms, taking into consideration the optimal library concentration and desired data volume. The clustering of the index coded samples was performed according to the manufacturer’s instructions. Following the generation of clusters, the library preparations underwent sequencing using an Illumina platform, resulting in the generation of paired-end reads.

#### RNA sequencing data analysis

Raw data (raw reads) were processed in fastq format utilizing the fastp software (RRID:SCR_016962). This step involved removing reads that contained adapters, reads with poly-N sequences, and low-quality reads from the raw data, resulting in obtaining clean data (clean reads). Additionally, metrics such as Q20, Q30 and GC content were calculated for the clean data. The reference genome (hg38) and gene model annotation files were directly downloaded from the genome website. To enable alignment of the paired-end clean reads, an index of the reference genome was constructed using Hisat2 v2.0.5 (RRID:SCR_015530). Subsequently, Hisat2 v2.0.5 was employed as the mapping tool of choice. To determine the number of reads mapped to each gene, featureCounts v1.5.0-p3 (RRID:SCR_012919) was employed. Subsequently, the Fragments Per Kilobase of transcript sequence per Millions (FPKM) base pairs sequenced for each gene was calculated based on the gene’s length and the count of reads mapped to it. Differential expression analysis was conducted on two conditions/groups using the DESeq2 R package (version 1.20.0, RRID: SCR_015687). The resulting *P*-values were adjusted using the Benjamini and Hochberg’s method to control the false discovery rate. Genes with an adjusted P-value of less than or equal to 0.05, as determined by DESeq2, were identified as differentially expressed. The read counts for each sequenced library were adjusted using the edgeR package (version 3.22.5, RRID:SCR_012802) by applying a scaling normalisation factor. The differential expression analysis of the two conditions was carried out using the edgeR. The *P*-values were adjusted using the Benjamini and Hochberg’s method. A corrected *P*-value threshold of 0.05 and an absolute fold change of 2 were set to determine significantly differential expression. The clusterProfiler (RRID:SCR_016884) R package was used for Gene Ontology (GO) (RRID:SCR_002811) enrichment analysis on the DEGs, with gene length bias being corrected. GO terms with a corrected *P*-value < 0.05 were deemed significantly enriched by the DEGs. For the analysis of KEGG (RRID:SCR_012773) pathways, the clusterProfiler R package was employed. The statistical enrichment of DEGs in KEGG pathways was assessed.

### Single nuclei RNA sequencing

#### Samples processing

Ten MOs, 10 StrOs and 4 assembloids per batch form 2 batches, generated from the same line (201, Supplementary Table 1) were used. The culture time point was D30 for assembloids, D50 for MOs and D65 for StrOs. MOs and StrOs that were cultured separately after D20 and D35, were cultured in the optimised co-culture medium. Samples from the two batches were pulled together for nuclei extraction and sequencing by Singleron.

#### Data analysis

Reads were mapped to Homo_sapiens_ensembl_92 genome, and 10× matrices were generated for each sample. To perform the downstream analysis, we used R studio (23.06.1 + 504 version, RRID:SCR_000432) and R 4.2.2 version (RRID:SCR_001905). Using the Seurat package (version 4.3.0, RRID:SCR_016341), Seurat objects were created for each sample and quality control filtering was performed. For all datasets, cells with >5% mitochondrial genes were filtered out. Additional filtering for cell debris and doublets was performed for each dataset. In MOs data cells with <100 and >1000 genes, in StrOs cells with <100 and >1800 genes and in assembloids cells with <100 and >4000 genes were filtered out. Similar to another study, after filtering, ribosomal and mitochondrial genes were removed from all datasets, as they are considered contamination in the single nuclei RNA sequencing experiments^66^. After filtering, using the standard Seurat workflow, we analyzed each dataset separately, for identifying clusters specific to each model. LogNormalisation was performed in each dataset, followed by the identification of the 2000 most variable genes (FindVariableFeatures function). Next, data were scaled with the ScaleData function and linear dimensionality reduction was performed with the RunPCA function. Ten PCs were used for the MO dataset clustering and 15 PCs for the StrO and assembloid datasets. Clusters were identified using the FindNeighbors and FindClusters functions, at 0.5 resolution in all datasets. Determination of cell type identity in each cluster was performed with the evaluation of specific cellular markers expression using the DotPlot visualization method. For identifying differentially expressed genes (DEGs) between assembloids and MOs or StrOs, integration analysis was performed^29^. After integrating the data, DEG lists using the FindMarkers function were computed, defining the assembloid dataset as ident.1 and the MO or StrO dataset as ident.2. The DEG lists from both comparisons were imported in the Metacore (Clarivate, 2023) online software, were enrichment analysis with FDR threshold >0.25 and adjusted *P*.value < 0.05 was performed. Enriched pathways from the “Process Networks” category were extracted. Genes related to the enrichment of the “Development_Neurogenesis_Axonal guidance” pathway were exported, and their expression pattern (up or downregulation) was evaluated. DEGs were also used to assess the expression pattern of genes related to neuronal maturity.

### Microelectrode array

#### Basal activity measurement in assembloids

Non-embedded assembloids were used for the electrophysiological analysis using the Axion Microelectrode array (MEA) system. Forty eight-well MEA plates (Axion, M768-tMEA-48B-5) were first coated with 0.1 mg/ml poly-D-lysine (Sigma-Aldrich, P7886) and incubated at 37 °C, 5% CO_2_ overnight, followed by 1 h incubation with 1 mg/ml laminin (Sigma-Aldrich, L2020). Laminin coating was removed, and the plates were washed twice with sterile PBS (Thermo Fisher Scientific, 14190250). Each assembloid was placed in the center of the well on the electrodes and after the media was carefully aspirated, it was left for 2 and 3 min to dry. Then, 15 μl of Geltrex was added on top of each assembloid and was left in the incubator for 5 min to polymerise. Five hundred microliters of fresh culture medium was added in each well and the plate was kept in the incubator (37 °C, 5% CO_2_) under static conditions. Electrophysiological data were acquired with the Axion Maestro Multiwell 768-channel MEA System (Axion Biosystems) and the Axis software (Axon Biosystems,RRID:SCR_016308). For the analysis of the data the spike lists were exported from the Axis software and the data were processed with MATALB (2021a, Mathworks,RRID:SCR_001622). Plots of the MATLAB exported data were generated using R 4.2.2 vesrion.

#### Connectivity measurement in assembloids

The assembloid electrical activity was measured by 8 × 8 microelectrode arrays (MEAs) positioned at the bottom of a 12 multiwell. The MEA signals were measured at a sample rate of 12.5 kHz (M768-GLx 12-well plate, Axion Biosystems). A microfluidic system laying on top of the MEA was designed to physically separate the MO and the StrO, allowing their connection only through microtunnels (10 µm width) where neurites can grow along both directions (Fig. 4a).

MEA recordings were pre-processed using the AxIS Navigator 3.9.1 software (Axion Biosystems). The spike events were extracted for each electrode using the Peak Detection Adaptive Threshold method, setting an amplitude threshold of 6 standard deviations (Fig. 4b). The timestamps of the extracted spikes were exported to MATLAB (2019b, Mathworks) for further analysis (https://github.com/dlpigozzi/CoMEA).

The degree of connectivity between different MEA regions was assessed by quantifying the correlation between the recorded spike trains. The STTC^67^ was calculated for each pair of electrodes using the open code repository MEA-NAP^68^. The time interval Δ*t* for the STTC computation was set at 10 ms to privilege the detection of mono-synaptic connections (Fig. 4c).

The cross-correlogram provides an estimate of the time delay and signal directionality between electrode pairs. A 0.5 ms time binning was utilized to plot the probability histogram of the time delay (Fig. 4d), where the histogram maximum corresponded to the (most probable) time *t* required by the signal to move between the two electrodes, e.g., from A to B. The propagation speed *V*_AB_ was computed as the ratio of the distance *d*_AB_ (in meters) between A and B and the selected time delay *t*_AB_ (Fig. 4d). Only maxima exceeding the histogram mean value by 5 standard deviations were considered as significant. An STTC cut-off of 0.3 was utilized as an additional criterion to accept the existence of a connection between the two electrodes. The signal propagation between A and B was graphically represented as a vector connecting the two electrodes with orientation dependent on the sign (positive or negative) of the time delay. The average signal directionality for a given electrode was computed as the vectorial sum of the vectors connecting it to the electrodes having both significant cross-correlation maximum and STCC (Fig. 4e).

The mean directionality of a given assembloid was computed as the vectorial sum of its electrode vectors. During the assembloid development, if the vertical component (y) of the resulting vector reached a value ≥2 (i.e., the distance between 2 electrodes), the connection between the MO and the StrO was considered as established (Fig. 4f).

For the 92% of the assembloids establishing connection, we calculated the mean vertical (y) and lateral (x) components of their directionality vectors. The vertical component can result positive or negative, depending on the overall direction of the signal from the MO to the StrO or from the StrO to the MO, respectively (Fig. 4g). Instead, the lateral component was calculated considering its absolute value (|x|) without distinction between left and right side of the MEA (Fig. 4h).

#### Statistics and reproducibility

Data were analysed using GraphPad Prism 9.0.0 or R studio (23.06.1 + 504 version) with R 4.2.2 version. Normality test was performed using the Shapiro test. If not stated otherwise, outlier removal was performed using the ROUT method Q 1% in GraphPad or the Inter-Quartile Range proximity rule in R and data were batch normalised to the mean value of each batch. For not normally distributed data, two-sided Wilcoxon test or Kruskal–Wallis with Dunn’s multiple comparison test and Benjamini–Hochberg correction was implemented. For normally distributed data, Welch’s *t*-test or one-way ANOVA with Tukey’s multiple comparison test was performed. Significant *P* value is represented with asterisks in the order *p* < 0.05 *, *p* < 0.01 **, *p* < 0.001 ***, *p* < 0.0001 ****. Error bars represent mean ± SD.

### Reporting summary

Further information on research design is available in the Nature Portfolio Reporting Summary linked to this article.

## Supplementary information

Supplementary Information

Description of Additional Supplementary File

Supplementary Data 1

Supplementary Data 2

Supplementary Data 3

Supplementary Data 4

Reporting Summary

## Data Availability

Raw and processed data that support the findings in this study, as well as scripts used for the analysis of the data are publicly available at this link: 10.17881/4va5-e156. The original Western blot images from Figs. 6 and 7, Supplementary Figs. 3 and 12 are included in Supplementary Figs. 17 and 18. Bulk RNA and single nuclei RNA sequencing data are available on Gene Expression Omnibus (GEO) under the accession codes GSE236458 and GSE241632 respectively.
