# Supplementary material for: Modeling early phenotypes of Parkinson’s disease by age-induced midbrain-striatum assembloids
Source: Commun Biol. 2024 Nov 23;7:1561. doi: 10.1038/s42003-024-07273-4 (PMC11585662; doi:10.1038/s42003-024-07273-4)
Supplement: Supplementary file 1 — Supplementary Information [file 42003_2024_7273_MOESM1_ESM.pdf]

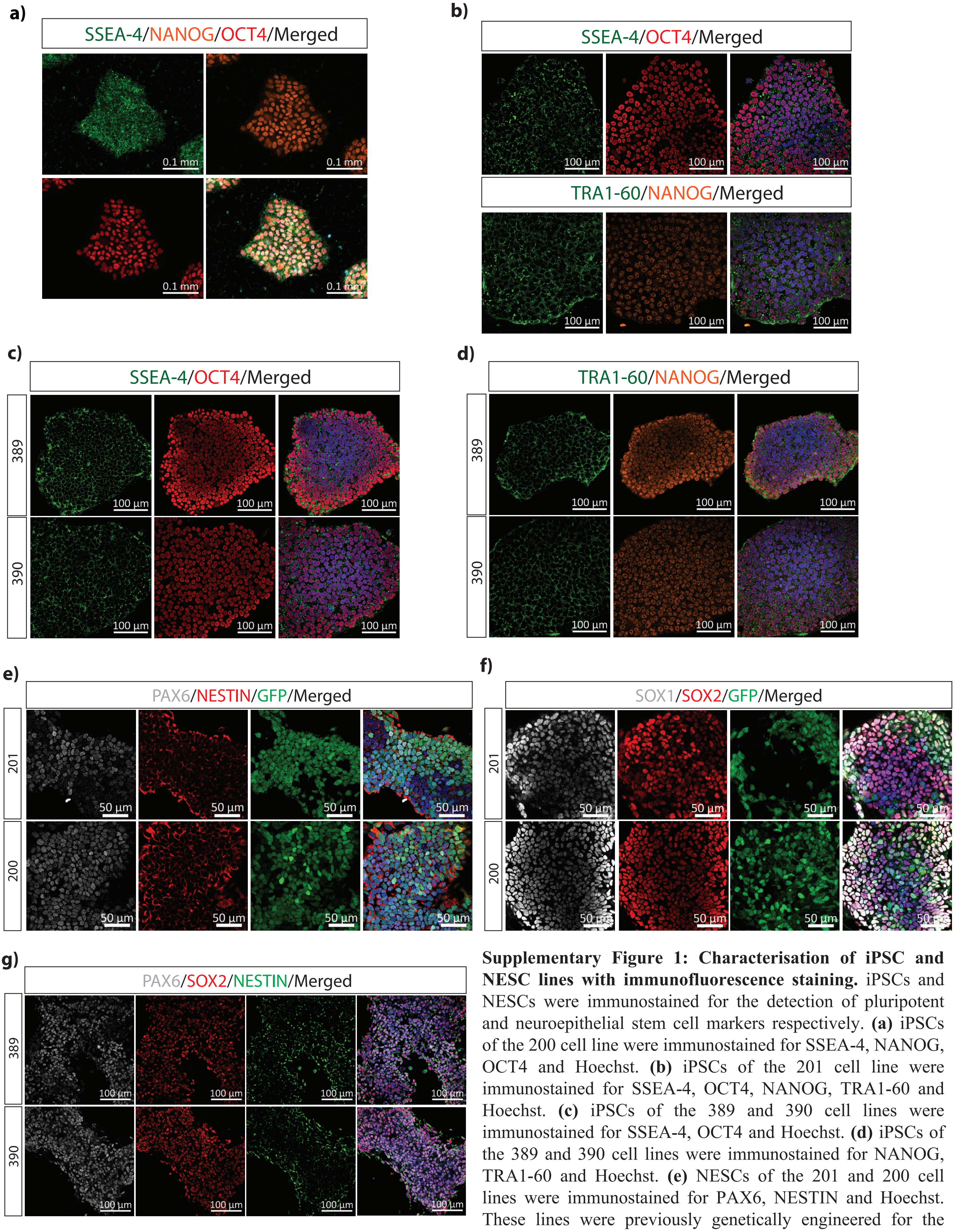

Supplementary Figure 1: Characterisation of iPSC and NESC lines with immunofluorescence staining. iPSCs and NESCs were immunostained for the detection of pluripotent and neuroepithelial stem cell markers respectively. **(a)** iPSCs of the 200 cell line were immunostained for SSEA-4, NANOG, OCT4 and Hoechst. **(b)** iPSCs of the 201 cell line were immunostained for SSEA-4, OCT4, NANOG, TRA1-60 and Hoechst. **(c)** iPSCs of the 389 and 390 cell lines were immunostained for SSEA-4, OCT4 and Hoechst. **(d)** iPSCs of the 389 and 390 cell lines were immunostained for NANOG, TRA1-60 and Hoechst. **(e)** NESCs of the 201 and 200 cell lines were immunostained for PAX6, NESTIN and Hoechst. These lines were previously genetically engineered for the intrinsic expression of GFP. **(f)** NESCs of the 201 and 200 cell lines were immunostained for SOX1, SOX2 and Hoechst. These lines were previously genetically engineered for the intrinsic expression of GFP. **(g)** NESCs of the 389 and 390 cell lines were immunostained for PAX6, SOX2, NESTIN and Hoechst.

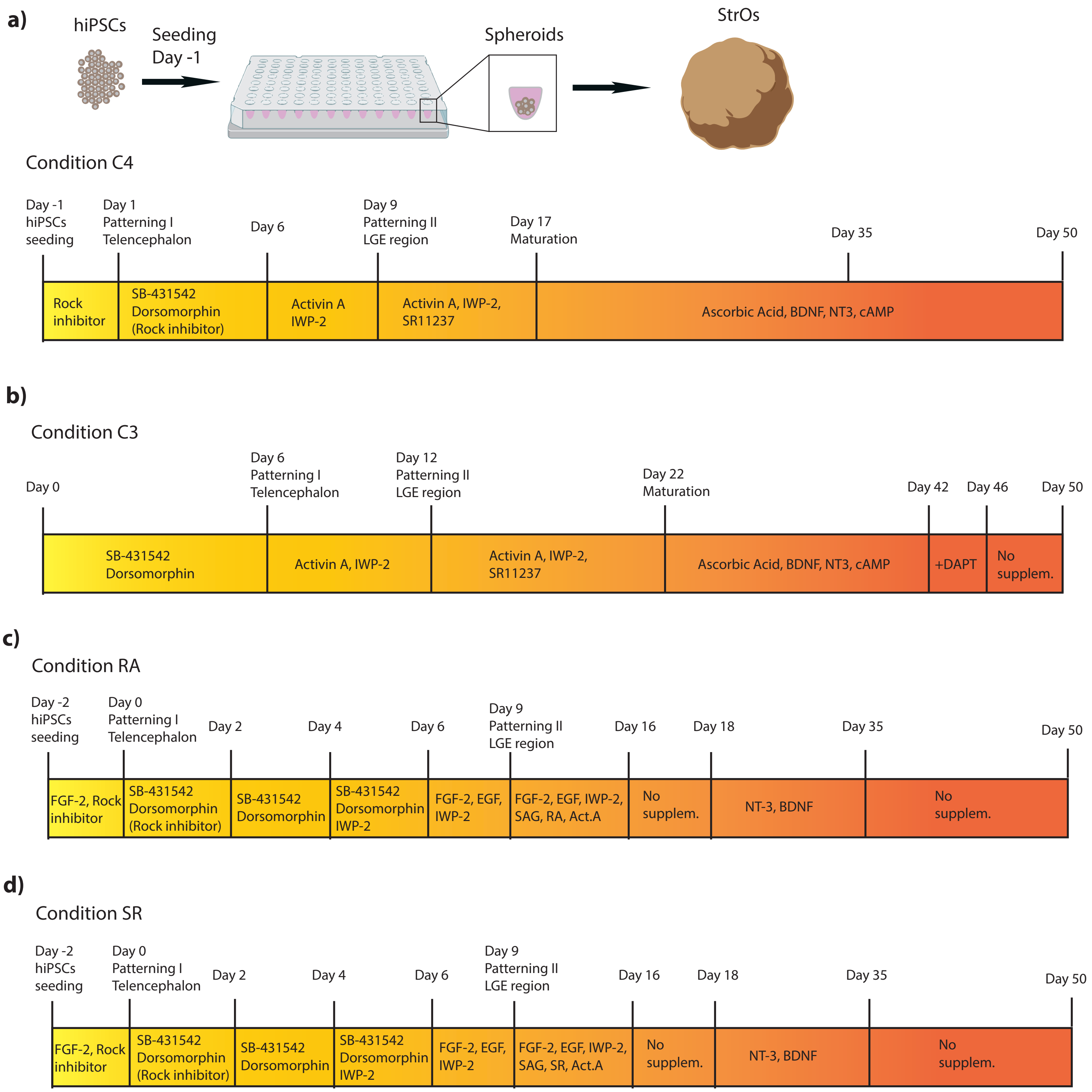

Supplementary Figure 2: Different conditions used for the generation of striatum organoids. (a-d) Schematic representation of the different steps in the differentiation process of StrOs generation in the condition C4 **(a),** in the condition C3 **(b)**, in the condition RA **(c)** and in the condition SR **(d)**.

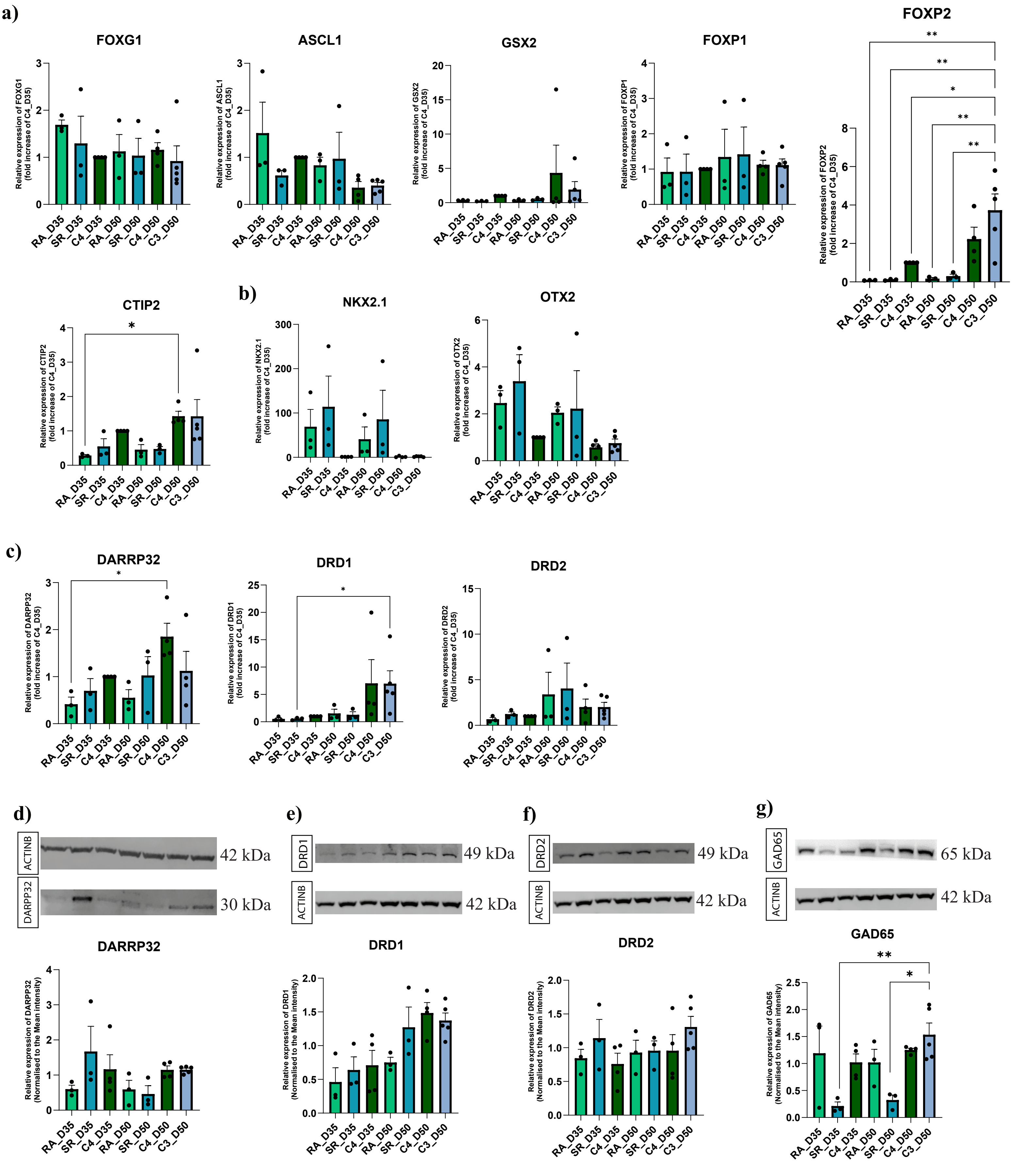
a)
FOXG1
Relative expression of FOXG1 (fold increase of C4_D35)
ASCL1
Relative expression of ASCL1 (fold increase of C4_D35)
GSX2
Relative expression of GSX2 (fold increase of C4_D35)
FOXP1
Relative expression of FOXP1 (fold increase of C4_D35)
FOXP2
Relative expression of FOXP2 (fold increase of C4_D35)
CTIP2
Relative expression of CTIP2 (fold increase of C4_D35)
b)
NKX2.1
Relative expression of NKX2.1 (fold increase of C4_D35)
OTX2
Relative expression of OTX2 (fold increase of C4_D35)
c)
DARRP32
Relative expression of DARPP32 (fold increase of C4_D35)
DRD1
Relative expression of DRD1 (fold increase of C4_D35)
DRD2
Relative expression of DRD2 (fold increase of C4_D35)
RA_D35 SR_D35 C4_D35 RA_D50 SR_D50 C4_D50 C3_D50
d)
ACTINB 42 kDa
DARPP32 30 kDa
e)
DRD1 49 kDa
ACTINB 42 kDa
f)
DRD2 49 kDa
ACTINB 42 kDa
g)
GAD65 65 kDa
ACTINB 42 kDa
DARRP32
Relative expression of DARPP32 (Normalised to the Mean intensity)
DRD1
Relative expression of DRD1 (Normalised to the Mean intensity)
DRD2
Relative expression of DRD2 (Normalised to the Mean intensity)
GAD65
Relative expression of GAD65 (Normalised to the Mean intensity)

Supplementary Figure 3: Striatum organoids characterisation. (a) qPCR plots showing the relative expression of progenitor markers specific to the LGE and striatum brain regions. For FOXG1, ASCL1, GSX2 and CTIP2, Kruskal-Wallis test with Benjamini-Hochberg correction and Dunn's multiple comparison test was performed. For FOXP1 and FOXP2, one-way ANOVA, with Tukey's multiple comparison test was performed. **(b)** qPCR plots showing the relative expression of progenitor markers specific to the MGE region of the brain. For NKX2.1, Kruskal-Wallis test with Benjamini-Hochberg correction and Dunn's multiple comparison test was performed. For OTX2, one-way ANOVA, with Tukey's multiple comparison test was performed. **(c)** qPCR plots showing the relative expression of striatum specific markers. For DARPP32, one-way ANOVA, with Tukey's multiple comparison test was performed. For DRD1 and DRD2, Kruskal-Wallis test with Benjamini-Hochberg correction and Dunn's multiple comparison test was performed. **(d)** Western blot showing the protein levels of DARPP32 in the different conditions of StrOs. One-way ANOVA, with Tukey's multiple comparison test was performed. **(e)** Western blot showing the protein levels of DRD1 in the different conditions of StrOs. One-way ANOVA, with Tukey's multiple comparison test was performed. **(f)** Western blot showing the protein levels of DRD2 in the different conditions of StrOs. One-way ANOVA, with Tukey's multiple comparison test was performed. **(g)** Western blot showing the protein levels of GAD65 in the different conditions of StrOs. One-way ANOVA, with Tukey's multiple comparison test was performed. For all plots, $n = 3\text{-}5$, where each point is one sample of pooled 6-8 pooled organoids, from 3-5 batches. *$p<0.05$, **$p<0.01$, ***$p<0.001$. qPCR data were normalised to ACTINB expression and fold change to the C4_D35 condition was calculated. For the western blots, data were normalised to β-Actin levels and batch correction was applied by normalising each value to the mean of the values for each batch. Error bars represent mean ± SD. In all plots, outliers were calculated in GraphPad Prism using the ROUT method Q 1%. Data were plotted in GraphPad Prism 9.0.0.

a)

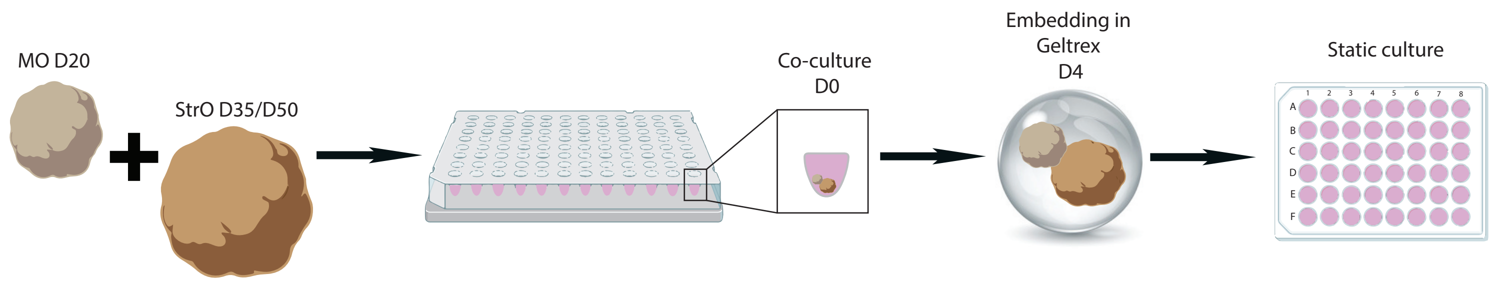

b)

	Media Composition	Small Molecules
Neural Medium	Neurobasal A, B27, Glutamax, P/S	-
Neural Medium Plus	Neurobasal A, **B27 plus**, Glutamax, P/S	-
Neural Medium ++	Neurobasal A, B27, Glutamax, P/S	AA, db cAMP, BDNF, GDNF,NT3
N2B27 ++	Neurobasal, DMEM/F12, B27, N2, Glutamax, P/S	AA, db cAMP, BDNF, GDNF,NT3

c)

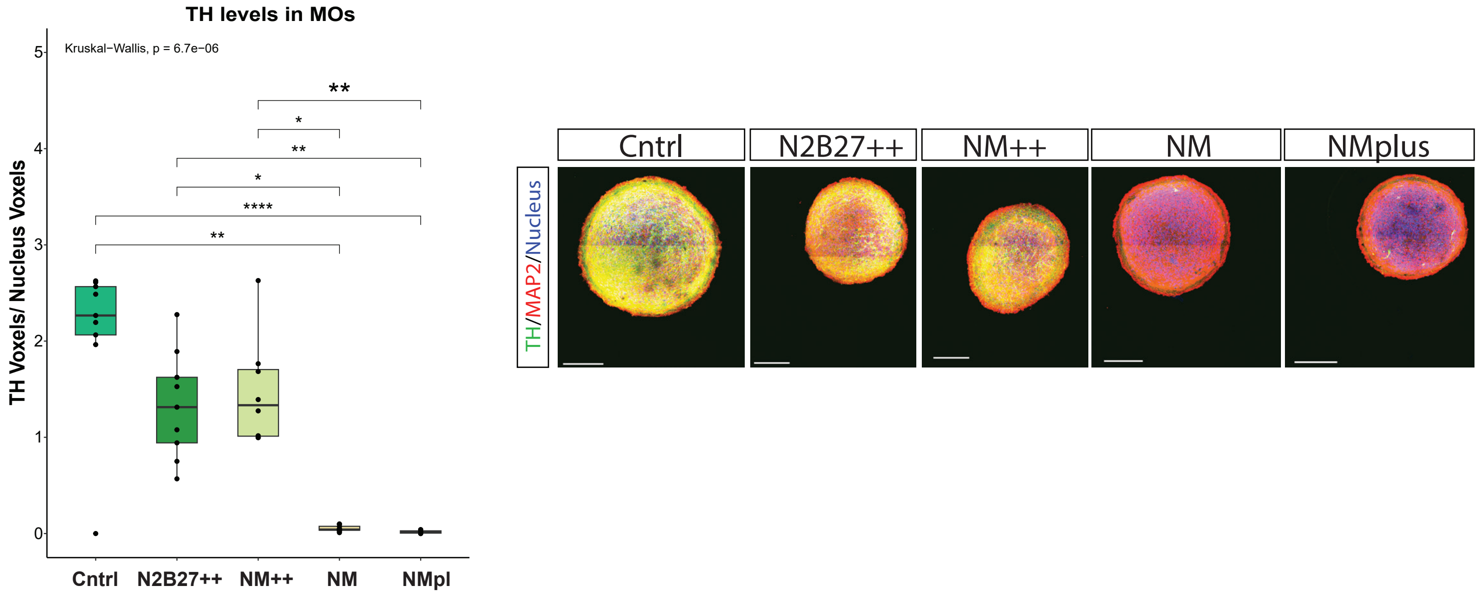

Supplementary Figure 4: Co-culture media optimisation for the assembloid model. (a) Schematic representation of the protocol used for the generation of assembloids. **(b)** Table showing the different media that were tested for the optimisation of the co-culture condition. **(c)** Immunostaining of MO 70 µm sections for the expression of TH, cultured for 20 days in the different co-culture conditions. Images were acquired with the Yokogawa high content imaging microscope. Kruskal-Wallis test with Benjamini-Hochberg correction and Dunn's multiple comparison test was performed. N = 6, where each point represents the average of 2 sections per organoids per batch, for 3 batches. Batch correction was applied by normalising each value to the mean of the values for each batch. *p<0.05, **p<0.01, ***p<0.001, ****p<0.001. Outlier removal was performed based on the Inter-Quartile Range (IQR) proximity rule. Data were plotted in R 4.2.2.

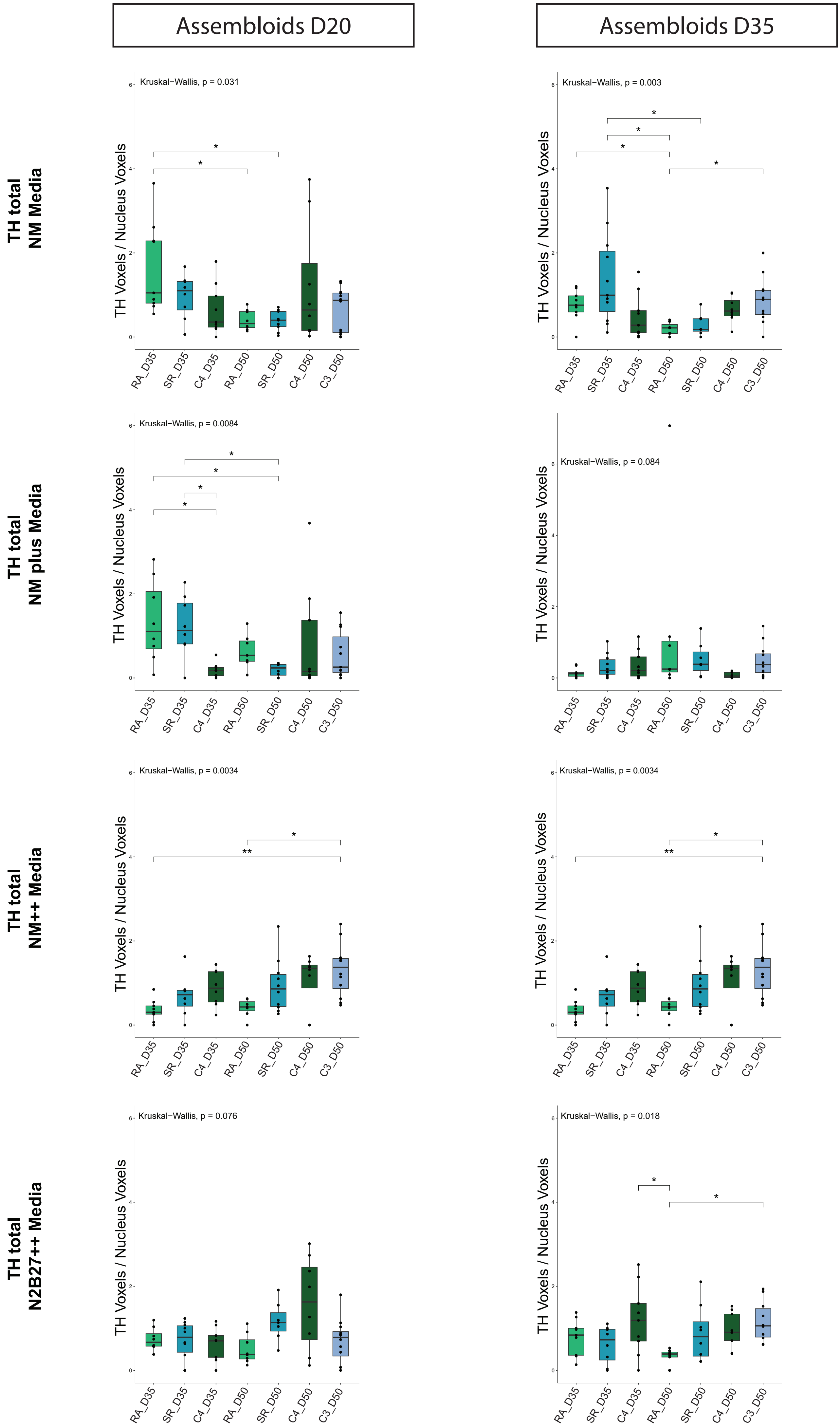

Assembloids D20
Assembloids D35
TH total
NM Media
TH total
NM plus Media
TH total
NM++ Media
TH total
N2B27++ Media
Kruskal–Wallis, p = 0.031
Kruskal–Wallis, p = 0.003
Kruskal–Wallis, p = 0.0084
Kruskal–Wallis, p = 0.084
Kruskal–Wallis, p = 0.0034
Kruskal–Wallis, p = 0.0034
Kruskal–Wallis, p = 0.076
Kruskal–Wallis, p = 0.018
TH Voxels / Nucleus Voxels
RA_D35
SR_D35
C4_D35
RA_D50
SR_D50
C4_D50
C3_D50

Supplementary Figure 5: Immunostaining for TH in assembloids cultured in the different co-culture conditions. 70 µm assembloid sections cultured for 20 and 35 days in the different co-culture and StrO conditions, were immunostained with TH and Hoechst for the quantification of the TH positive cells. Images were acquired with the Yokogawa high content imaging microscope. Kruskal-Wallis test with Benjamini-Hochberg correction and Dunn's multiple comparison test was performed. N = 6-9, where each point represents one section, per assembloid, per batch, for 3 batches. In all plots batch correction was applied by normalising each value to the mean of the values for each batch. *$p<0.05$, **$p<0.01$, ***$p<0.001$. Outlier removal was performed based on the Inter-Quartile Range (IQR) proximity rule. Data were plotted in R 4.2.2.

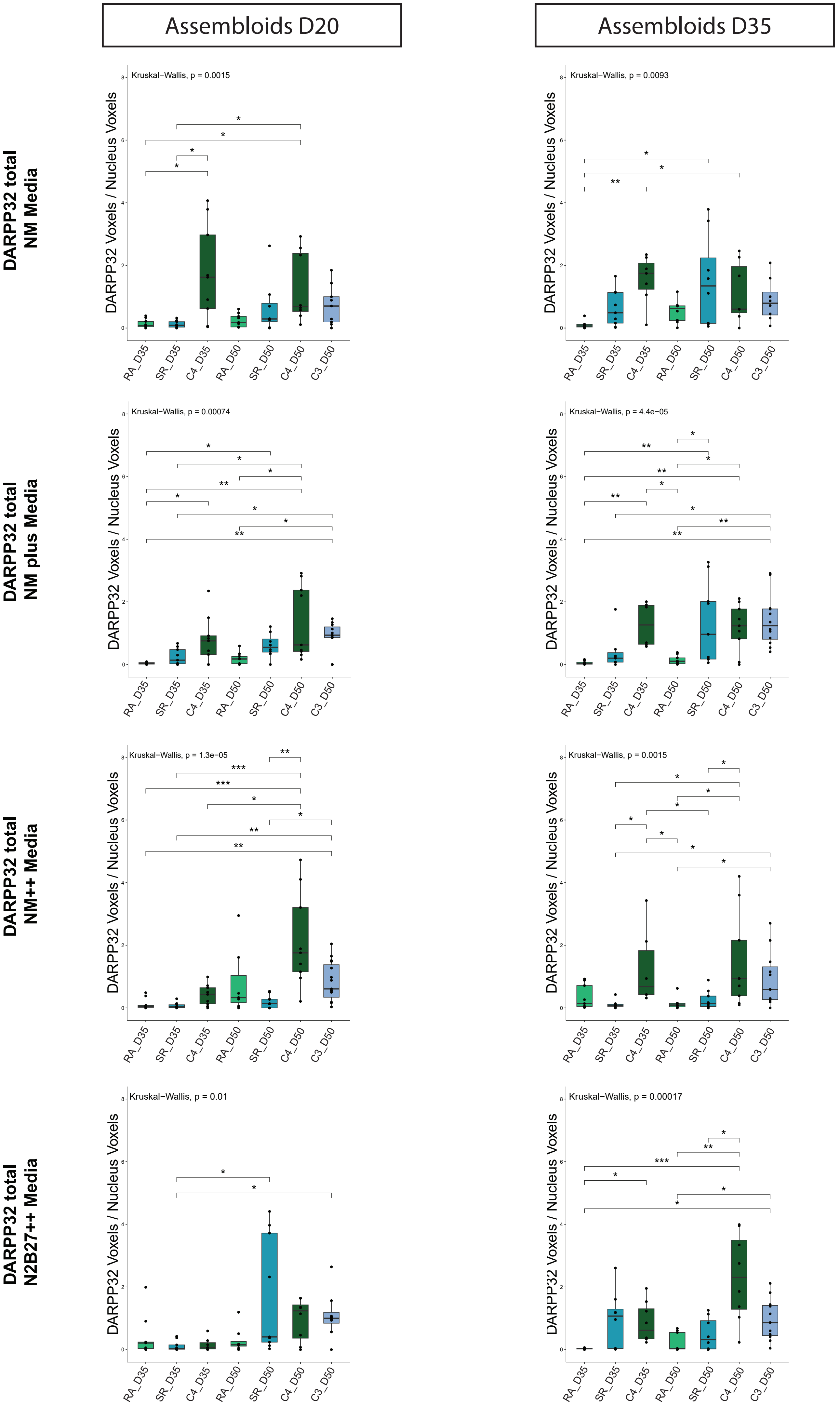
Assembloids D20
Assembloids D35
DARPP32 total NM Media
DARPP32 total NM plus Media
DARPP32 total NM++ Media
DARPP32 total N2B27++ Media
DARPP32 Voxels / Nucleus Voxels
Kruskal−Wallis, p = 0.0015
Kruskal−Wallis, p = 0.0093
Kruskal−Wallis, p = 0.00074
Kruskal−Wallis, p = 4.4e−05
Kruskal−Wallis, p = 1.3e−05
Kruskal−Wallis, p = 0.0015
Kruskal−Wallis, p = 0.01
Kruskal−Wallis, p = 0.00017
RA_D35
SR_D35
C4_D35
RA_D50
SR_D50
C4_D50
C3_D50

Supplementary Figure 6: Immunostaining for DARPP32 in assembloids cultured in the different co-culture conditions. 70 µm assembloid sections cultured for 20 and 35 days in the different co-culture and StrO conditions, were immunostained with DARPP32 and Hoechst for the quantification of the DARPP32 positive cells. Images were acquired with the Yokogawa high content imaging microscope. Kruskal-Wallis test with Benjamini-Hochberg correction and Dunn's multiple comparison test was performed. N = 6-9, where each point represents one section, per assembloid, per batch, for 3 batches. In all plots batch correction was applied by normalising each value to the mean of the values for each batch. *$p<0.05$, **$p<0.01$, ***$p<0.001$. Outlier removal was performed based on the Inter-Quartile Range (IQR) proximity rule. Data were plotted in R 4.2.2.

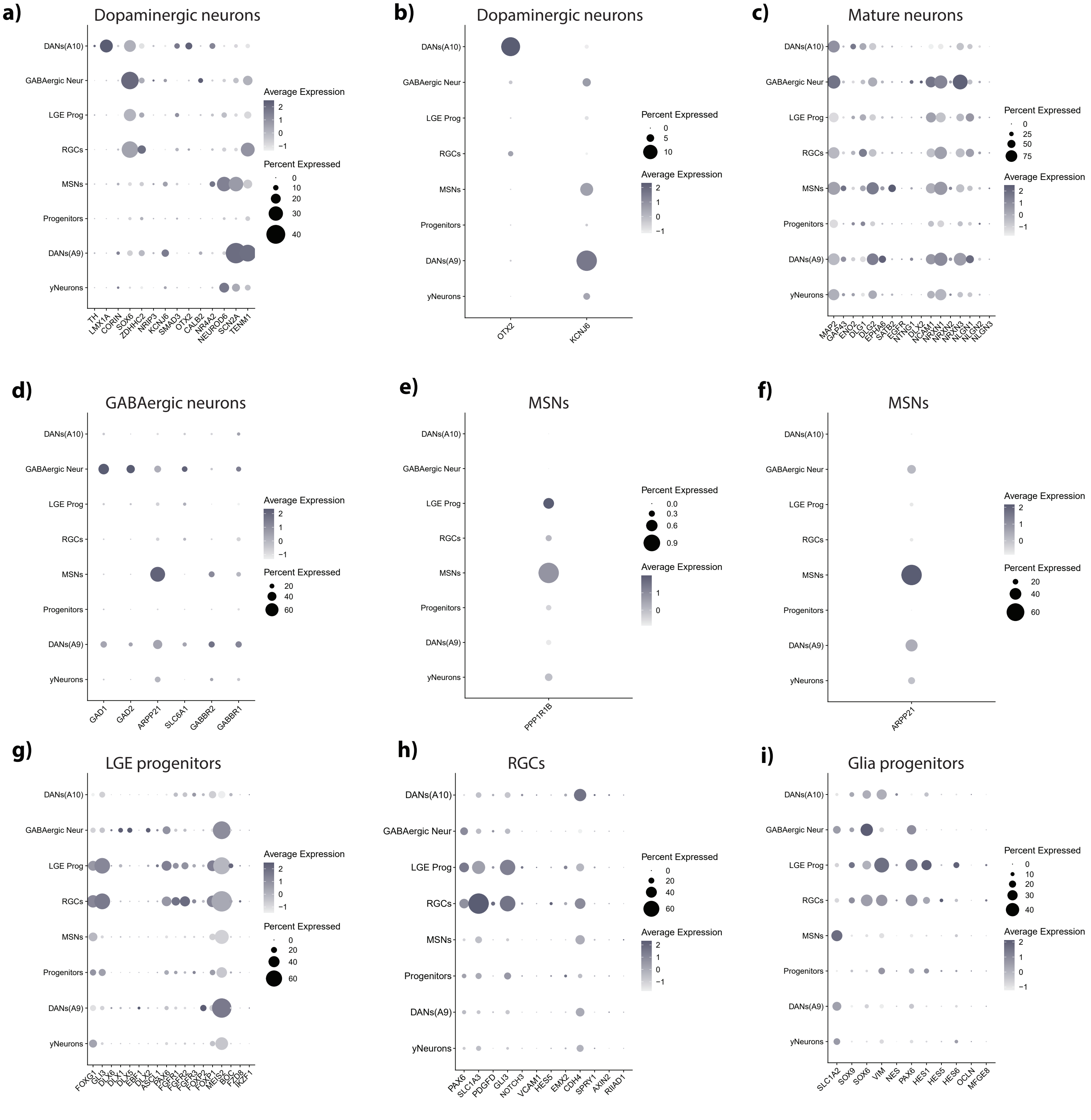

Supplementary Figure 7: Markers expression in the different clusters of assembloids, identified by single nuclei RNA sequencing analysis. (a-i) Dot plots showing the expression of markers specific to dopaminergic neurons (DANs) **(a-b)**, mature neurons **(c)**, GABAergic neurons **(d),** medium spiny neurons (MSNs) **(e-f)**, LGE progenitors **(g),** radial glia cells (RGCs) **(h)** and glia progenitors **(i)**.

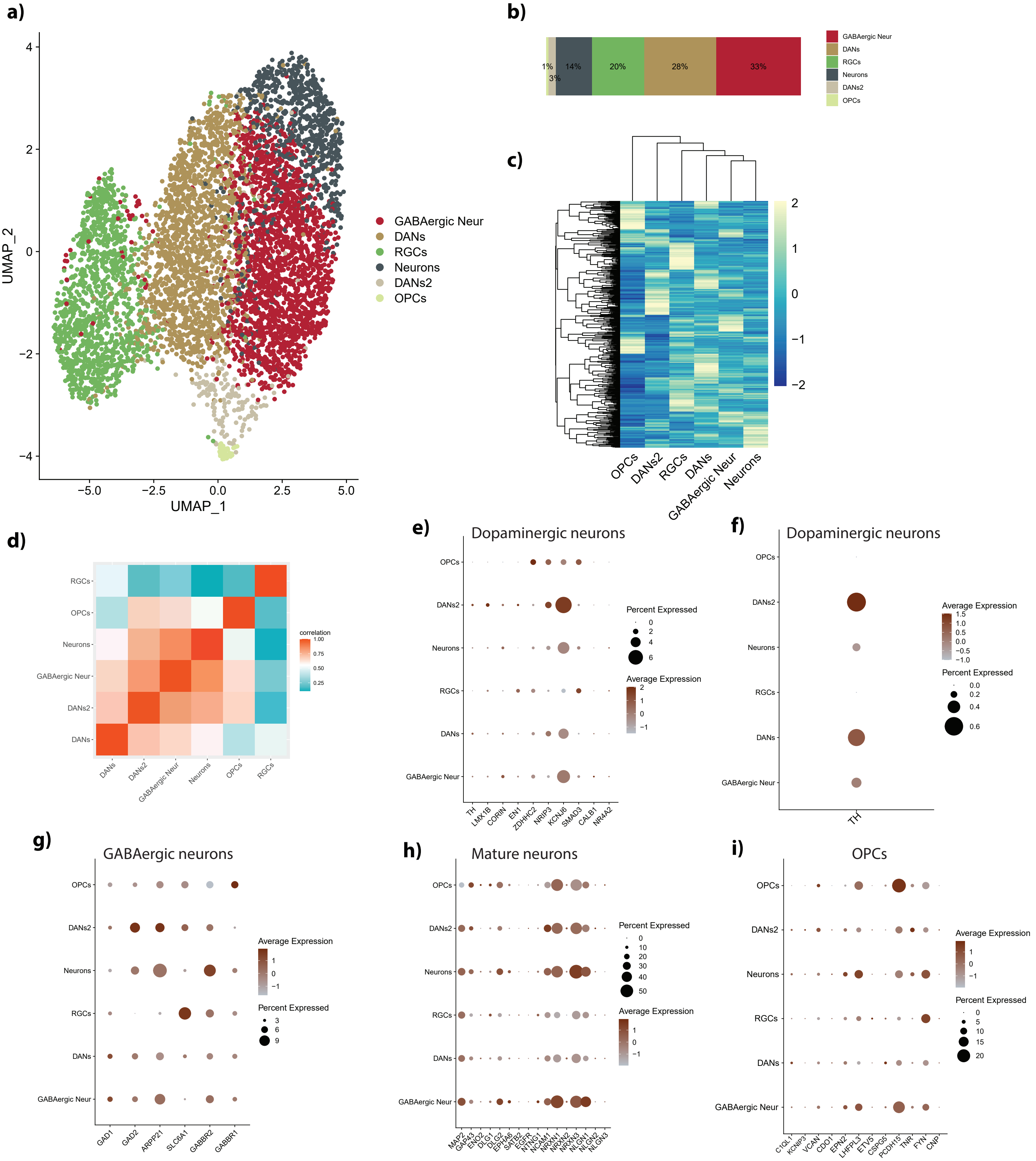

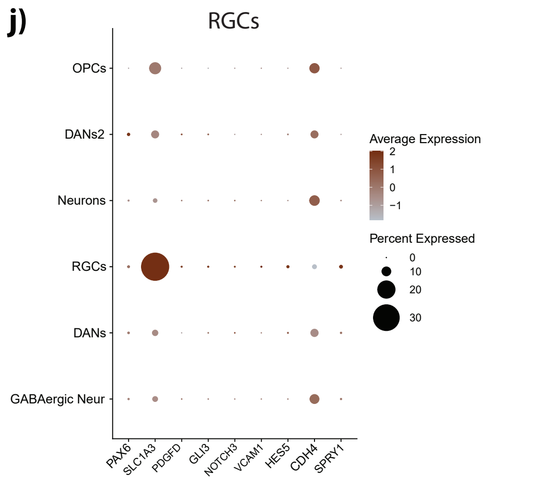

Supplementary Figure 8: Single nuclei RNA sequencing analysis of midbrain organoids. **(a)** UMAP embedding of the different cellular clusters in MOs. **(b)** Percentage of the cellular composition in MOs. **(c)** Unsupervised hierarchical clustering of cell clusters, using the average expression with Z-score normalisation of the top 500 most variable genes. **(d)** Spearman's correlation between the different cell types in MOs. **(e-j)** Dot plots showing the expression of markers specific to dopaminergic neurons **(e-f)**, GABAergic neurons **(g)**, mature neurons **(h)**, oligodendrocyte precursor cells (OPCs) **(i)** and radial glia cells (RGCs) **(j)**.

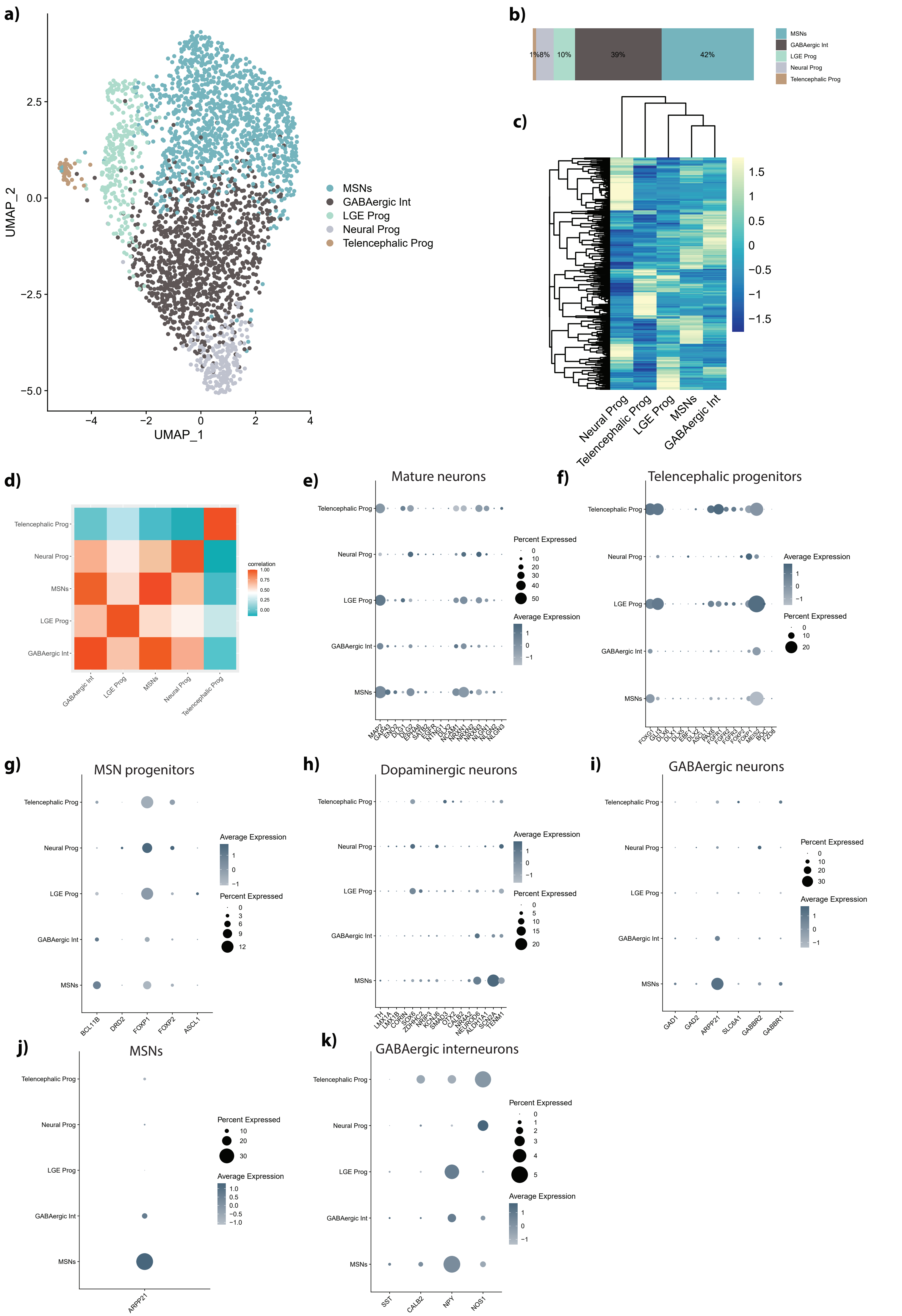

a)
UMAP_2
UMAP_1
MSNs
GABAergic Int
LGE Prog
Neural Prog
Telencephalic Prog
b)
1%8%
10%
39%
42%
c)
Neural Prog
Telencephalic Prog
LGE Prog
MSNs
GABAergic Int
d)
correlation
e)
Mature neurons
Percent Expressed
Average Expression
MAP2
GAP43
ENO2
DLG1
DLG2
EPHA6
SATB2
EGFR
NTNG1
NCAM1
NRXN1
NRXN2
NRXN3
NLGN1
NLGN2
NLGN3
f)
Telencephalic progenitors
FOXG1
GLI3
DLX6
DLX1
DLX5
EBF1
DLX2
ASCL1
PAX6
FGFR1
FGFR2
FGFR3
FOXP2
FOXP1
MEIS2
BOC
FZD8
g)
MSN progenitors
BCL11B
DRD2
FOXP1
FOXP2
ASCL1
h)
Dopaminergic neurons
TH
LMX1A
LMX1B
CORIN
SOX6
ZDHHC2
NRIP3
KCNJ6
SMAD3
OTX2
CALB2
NR4A2
ALDH1A1
SCN2A
TENM1
i)
GABAergic neurons
GAD1
GAD2
ARPP21
SLC6A1
GABBR2
GABBR1
j)
MSNs
ARPP21
k)
GABAergic interneurons
SST
CALB2
NPY
NOS1

Supplementary Figure 9: Single nuclei RNA sequencing analysis of striatum organoids. **(a)** UMAP embedding of the different cellular clusters in StrOs. **(b)** Percentage of the cellular composition in StrOs. **(c)** Unsupervised hierarchical clustering of cell clusters, using the average expression with Z-score normalisation of the top 500 most variable genes. **(d)** Spearman's correlation between the different cell types in StrOs. **(e-k)** Dot plots showing the expression of markers specific to mature neurons **(e)**, Telencephalic progenitors **(f)**, MSNs progenitors **(g)**, dopaminergic neurons **(h)**, GABAergic neurons and MSNs **(i-j)** and GABAergic interneurons **(k)**.

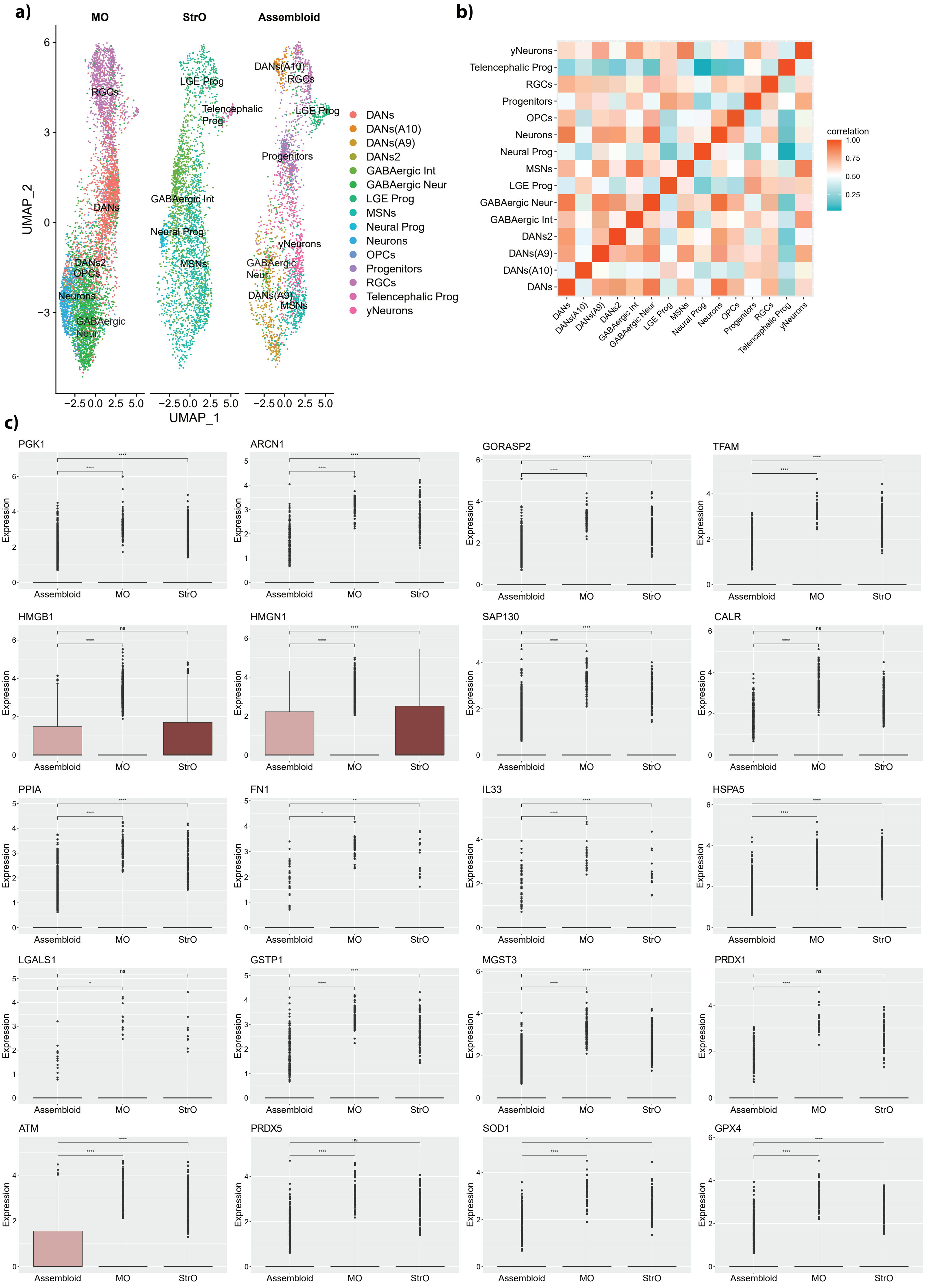

a)
MO
StrO
Assembloid
UMAP_2
UMAP_1
RGCs
DANs
DANs2
OPCs
Neurons
GABAergic Neur
LGE Prog
Telencephalic Prog
GABAergic Int
Neural Prog
MSNs
DANs(A10)
Progenitors
yNeurons
GABAergic Neur
DANs(A9)
DANs
DANs(A10)
DANs(A9)
DANs2
GABAergic Int
GABAergic Neur
LGE Prog
MSNs
Neural Prog
Neurons
OPCs
Progenitors
RGCs
Telencephalic Prog
yNeurons
b)
correlation
1.00
0.75
0.50
0.25
c)
PGK1
ARCN1
GORASP2
TFAM
HMGB1
HMGN1
SAP130
CALR
PPIA
FN1
IL33
HSPA5
LGALS1
GSTP1
MGST3
PRDX1
ATM
PRDX5
SOD1
GPX4
Expression
Assembloid
MO
StrO

Supplementary Figure 10: Single nuclei RNA sequencing integration analysis. (a) UMAP embedding of the integrated object, showing the predefined cellular identities in each model. **(b)** Spearman's correlation between the different cell types. **(c)** Expression of genes related to cellular and oxidative stress response in assembloids compared to MOs and StrOs. Dots represent single cells. Two-sided Wilcoxon test was performed between assembloids-MOs and assembloids-StrOs. *p<0.05, **p<0.01, ***p<0.001, ****p<0.0001. Data were plotted in R 4.2.2.

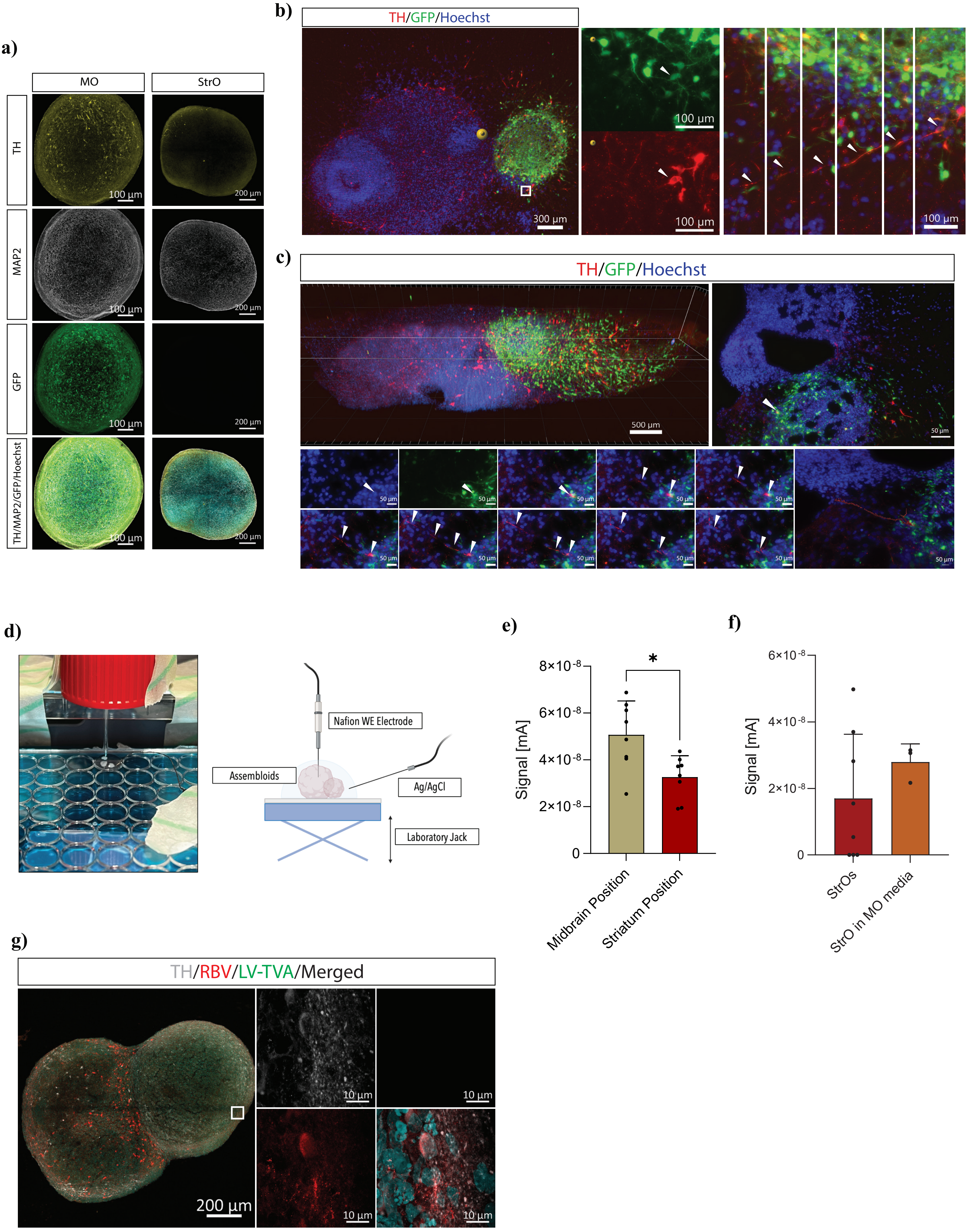
a)
MO
StrO
TH
MAP2
GFP
TH/MAP2/GFP/Hoechst
100 µm
200 µm
b)
TH/GFP/Hoechst
300 µm
100 µm
c)
TH/GFP/Hoechst
500 µm
50 µm
d)
Nafion WE Electrode
Assembloids
Ag/AgCl
Laboratory Jack
e)
Signal [mA]
8×10-8
6×10-8
4×10-8
2×10-8
0
*
Midbrain Position
Striatum Position
f)
Signal [mA]
6×10-8
4×10-8
2×10-8
0
StrOs
StrO in MO media
g)
TH/RBV/LV-TVA/Merged
200 µm
10 µm

Supplementary Figure 11: Nigrostriatal connectivity in the assembloid model. (a) Representative confocal images of MO and StrO 70 µm sections immunostained for TH, MAP2 and Hoechst. **(b)** Representative light sheet microscopic image (12X objective) of a whole assembloid and a ROI. The assembloid was immunostained with Hoechst and TH. The ROI shows the GFP+/TH+ neuron's soma in the MO-GFP side of the assembloid with TH+ projections towards the striatum side in the different planes (Z.236-280) along the Z stack. White arrowheads show the progression of the TH+ projection in images from the different planes. **(c)** Representative light sheet microscopic image of a whole assembloid (4X objective) and the ROI (25X objective), in light sheet fluorescence microscopy. The assembloid was immunostained with Hoechst and TH. The ROI shows the GFP+/TH+ neuron's soma in the MO-GFP side of the assembloid with TH+ projections towards the striatum side in the different planes along the Z stack (Z.660-755). White arrowheads show the progression of the TH+ projection in images from the different planes. 3D reconstruction of the neuron across the planes shows the complete TH+ neuronal projection. **(d)** Schematic representation of the electrochemical measurement set up in assembloids. **(e)** Barplot showing the electrochemical measurements of catecholamines in tissue approximate to the MO and StrO sides of the assembloid model. Welch's t-test was performed. N = 8, where each point represents the average of the 3 technical electrochemical measurements in the MO and StrO side respectively, in each assembloid, for 2 batches. Background measurement was not subtracted in these measurements. Data were plotted in GraphPad Prism 9.0.0. *$p<0.05$, **$p<0.01$, ***$p<0.001$. Error bars represent mean ± SD. **(f)** Bar plot showing the electrochemical measurements of catecholamines in StrOs cultured in their normal conditions and StrOs cultured in pre-used media in MOs culture. Welch's t-test was performed. N = 3-8, where each point represents the average measurement value with the background subtracted (PBS measurements) of each organoid per batch, for 3 batches. Data were plotted in GraphPad Prism 9.0.0. *$p<0.05$, **$p<0.01$, ***$p<0.001$. Error bars represent mean ± SD. **(g)** Representative confocal images of a 70 µm assembloid section showing the GFP and RFP positive cells from the LV-GP-TVA-GFP and RBV-ΔG-EnvA-RFP infections respectively and immunostained with Hoechst and TH. Close up images indicated by the white square, show the colocalization of Rabies and TH signal in the MO side of the assembloid.

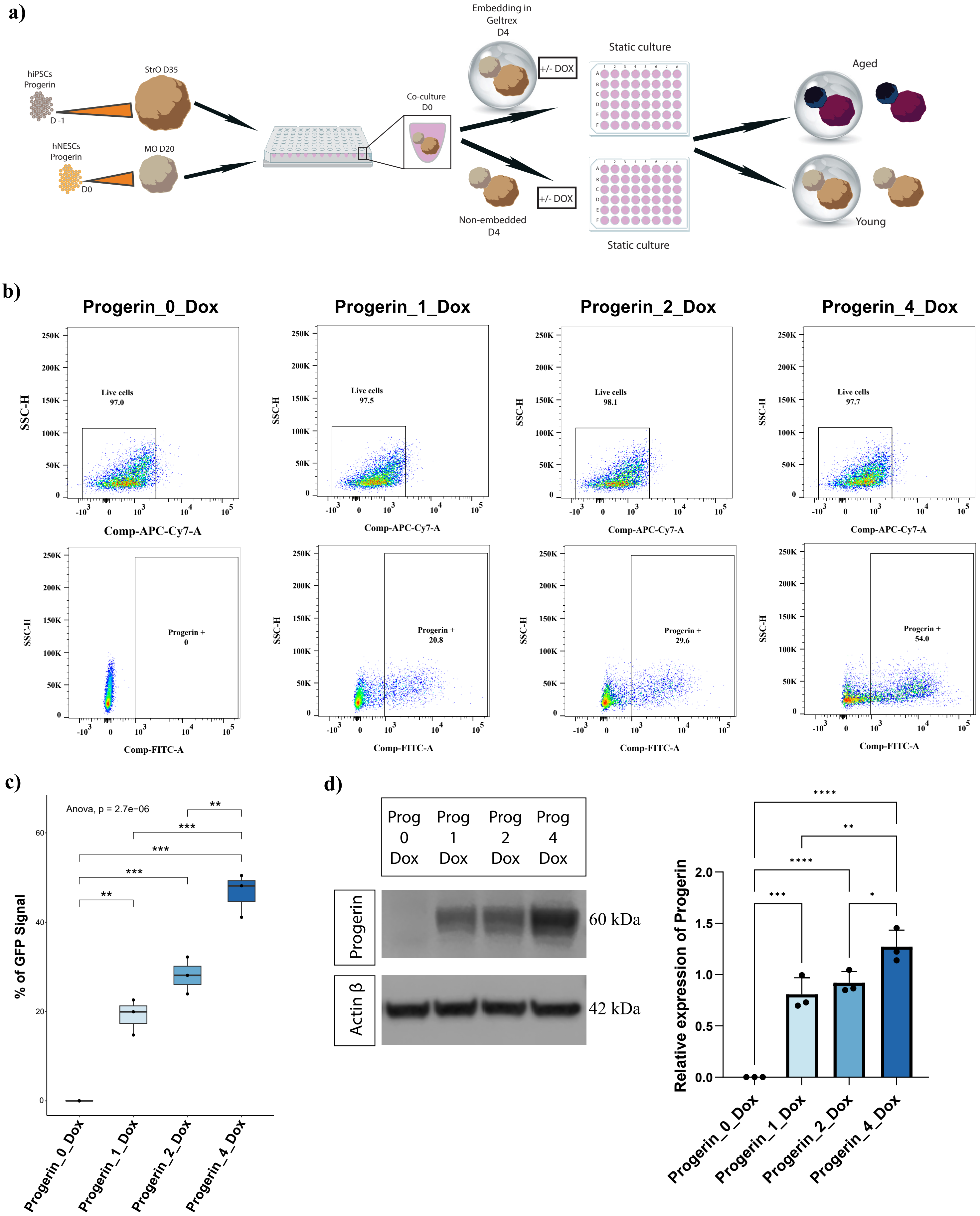
a)
hiPSCs
Progerin
D -1
StrO D35
hNESCs
Progerin
D0
MO D20
Co-culture
D0
Embedding in
Geltrex
D4
+/- DOX
Non-embedded
D4
+/- DOX
Static culture
Static culture
Aged
Young
b)
Progerin_0_Dox
Progerin_1_Dox
Progerin_2_Dox
Progerin_4_Dox
Live cells
97.0
Live cells
97.5
Live cells
98.1
Live cells
97.7
SSC-H
Comp-APC-Cy7-A
Progerin +
0
Progerin +
20.8
Progerin +
29.6
Progerin +
54.0
Comp-FITC-A
c)
Anova, p = 2.7e-06
% of GFP Signal
Progerin_0_Dox
Progerin_1_Dox
Progerin_2_Dox
Progerin_4_Dox
d)
Prog 0 Dox
Prog 1 Dox
Prog 2 Dox
Prog 4 Dox
Progerin
Actin β
60 kDa
42 kDa
Relative expression of Progerin
Progerin_0_Dox
Progerin_1_Dox
Progerin_2_Dox
Progerin_4_Dox

Supplementary Figure 12: Optimisation of the Progerin-overexpression in the assembloid model. (a) Schematic representation of the assembloid model generation using the Progerin cell line for the inducible overexpression of Progerin after doxycycline supplementation. **(b)** FACs pseudocolor plots showing the percentage of Live (Zombie, APC-Cy7) and Progerin (FITC) positive cells. **(c)** FACs data showing the % of the GFP positive signal measured in live cells of dissociated assembloids, treated with different doxycycline concentrations (Progerin_0_Dox = Untreated, Progerin_1_Dox = 1 ng/μl, Progerin_2_Dox = 2 ng/μl, Progerin_4_Dox = 4 ng/μl). One-way ANOVA, with Tukey's multiple comparison test was performed in R 4.2.2. For all conditions n = 3 with each point representing the average of two technical replicates per batch for 3 batches. *$p<0.05$, **$p<0.01$, ***$p<0.001$. **(d)** Western blot for Progerin protein levels in assembloids treated with the different doxycycline concentrations. One-way ANOVA, with Tukey's multiple comparison test was performed in R 4.2.2. Error bars represent mean ± SD. For all conditions n =3 with each point representing 3-4 polled assembloids per batch, for 4 batches. *$p<0.05$, **$p<0.01$, ***$p<0.001$. Batch correction was applied by normalising each value to the mean of the values for each batch. Outliers were calculated in GraphPad Prism using the ROUT method Q 1%.

a)

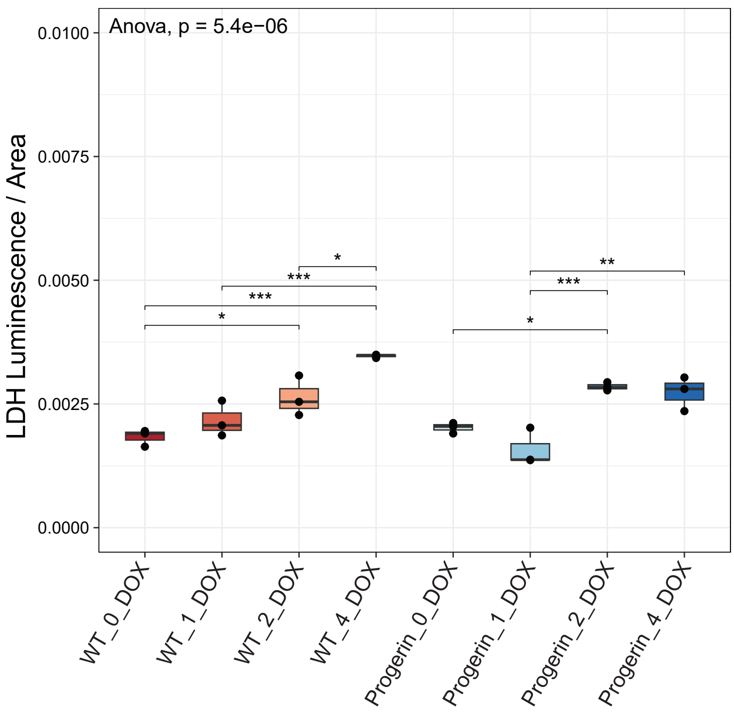

b)

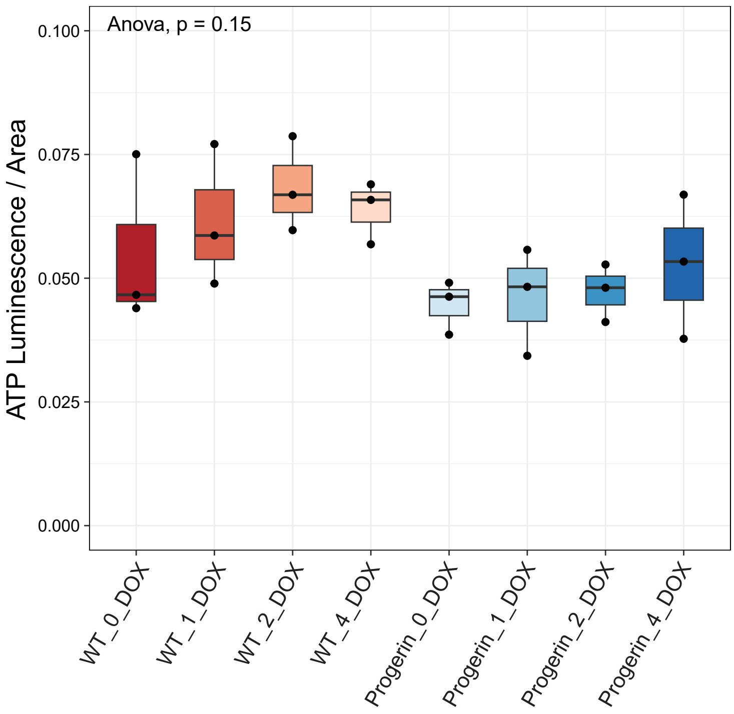

Supplementary Figure 13: Toxicity assessment of doxycycline. (a) Boxplot showing the quantification of the LDH luminescence signal normalised to the average area of assembloids. One-way ANOVA, with Tukey's multiple comparison test was performed. N = 3, where each point represents the average of 3 technical replicates per batch, for 3 batches. Statistics show the pairwise comparisons of interest between the treatments in the same condition (Progerin or WT) and between the conditions with the same treatment. **(b)** Boxplot showing the quantification of the ATP luminescence signal normalised to the average area of assembloids. One-way ANOVA, with Tukey's multiple comparison test was performed. N = 3, where each point represents the average of two technical replicates per batch, for 3 batches. *p<0.05, **p<0.01, ***p<0.001. Data were plotted in R 4.2.2.

a)

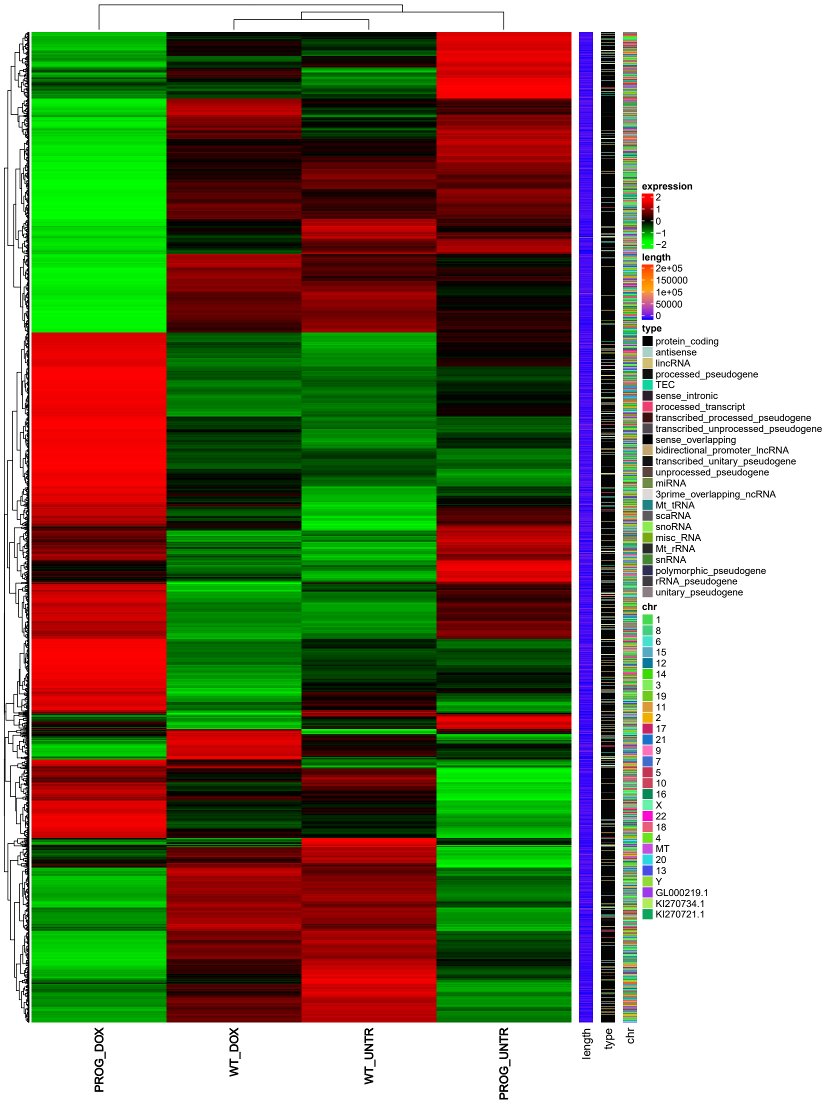

b)

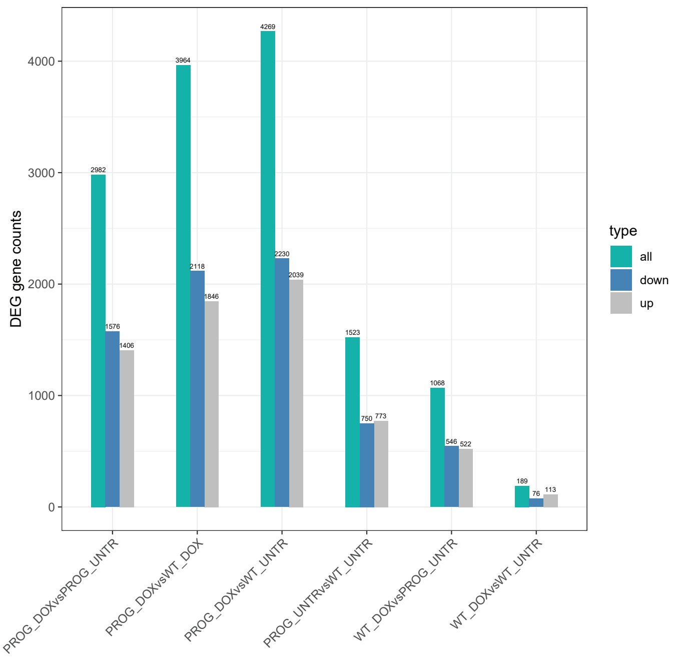

Supplementary Figure 14: Differential expression gene profile in aged and young assembloids. (a) Heatmap showing the hierarchical clustering of samples with similar expression patterns, after Z-score homogenisation of the expression data. **(b)** Histogram showing the number of differential genes (up-regulated and down-regulated) for each comparison.

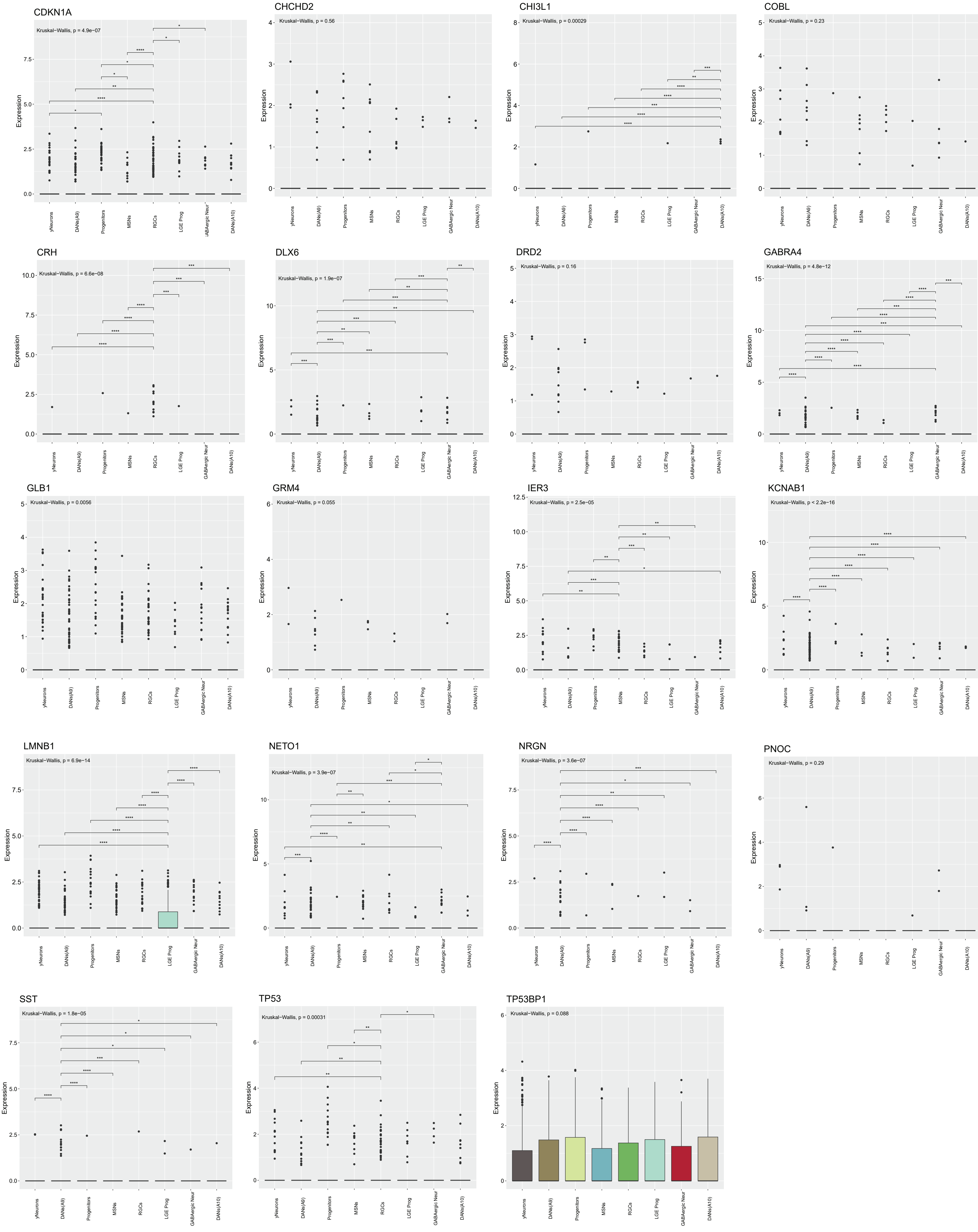

Supplementary Figure 15: Senescence and aging-related genes expression in the different cell populations of D30 assembloids. Kruskal-Wallis test with Benjamini-Hochberg correction and Dunn's multiple comparison test was performed. Dots represent single cells. *p<0.05, **p<0.01, ***p<0.001, ****p<0.0001. Data were plotted in R 4.4.1.

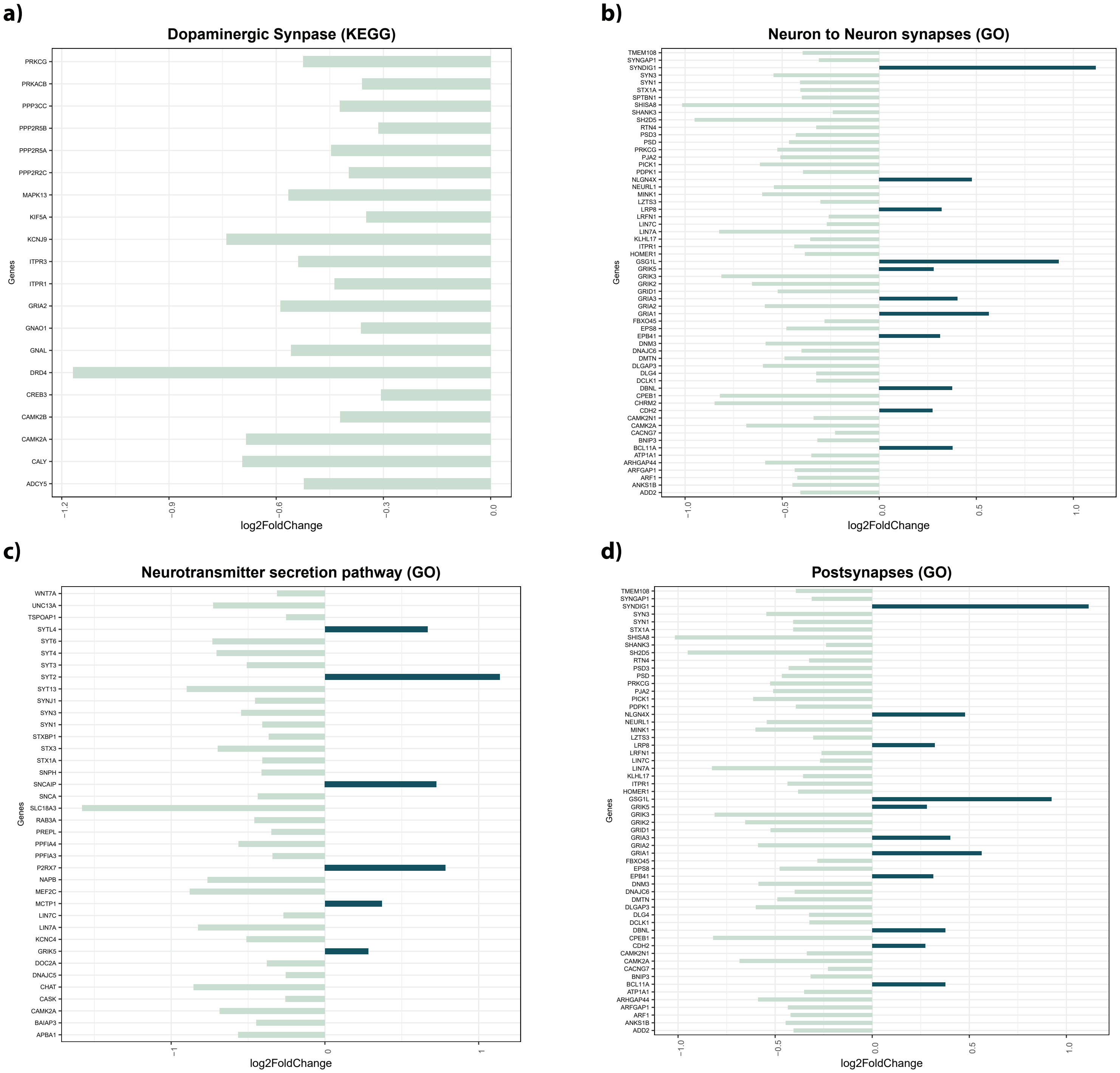

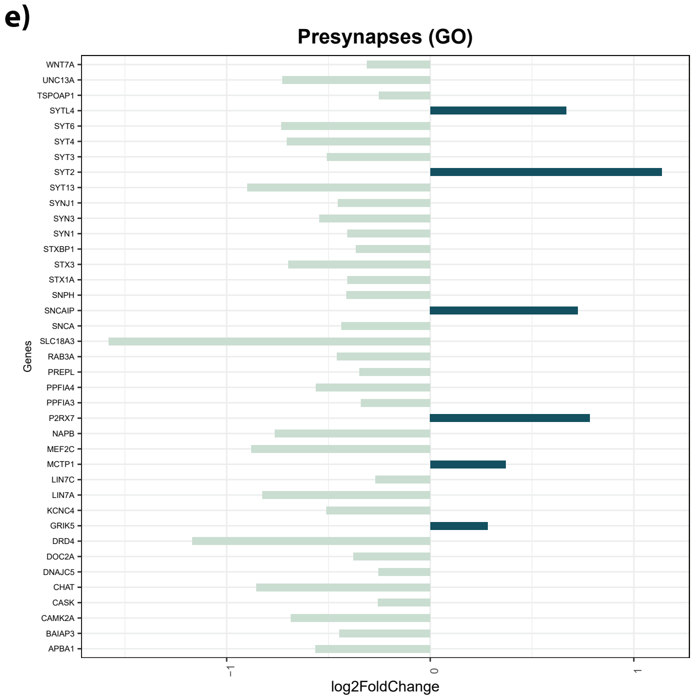

Supplementary Figure 16: Genes expression relevant to the synaptic pathways in GO and KEGG enrichment analysis. Fold change expression of genes in progerin-overexpressing assembloids (PROG_DOX) vs non-progerin-expressing assembloids (PROG_UNTR), responsible for the enrichment of Dopaminergic Synapse KEGG pathway **(a)**, Neuron to neuron synapses GO pathway **(b)**, Neurotransmitter secretion GO pathway **(c)**, Postsynapses GO pathway **(d)** and Presynapses GO pathway **(e)**.

Fig. 6c

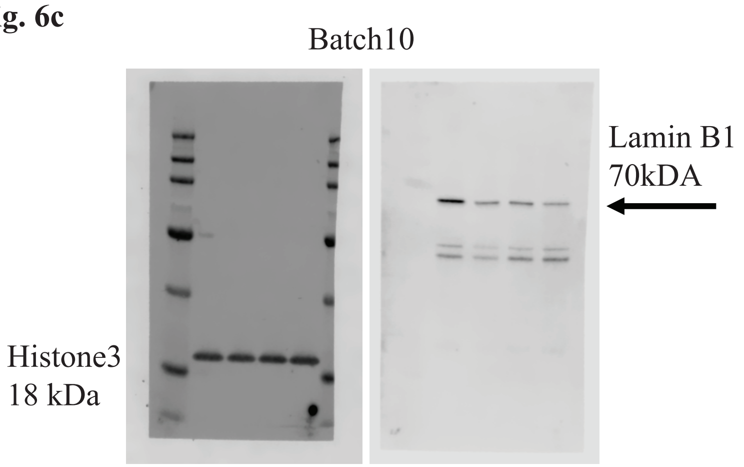

Fig. 7c (Batch10)

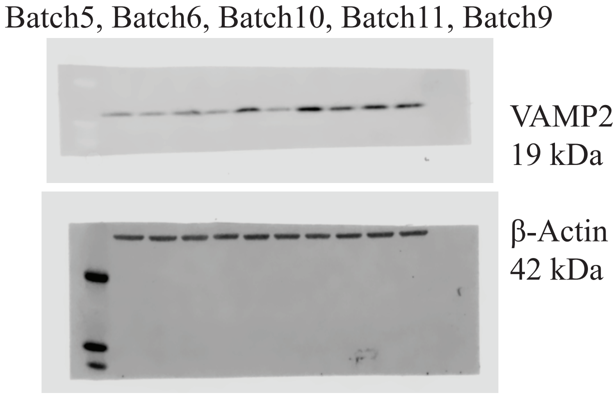

Fig. 7d (Batch10)

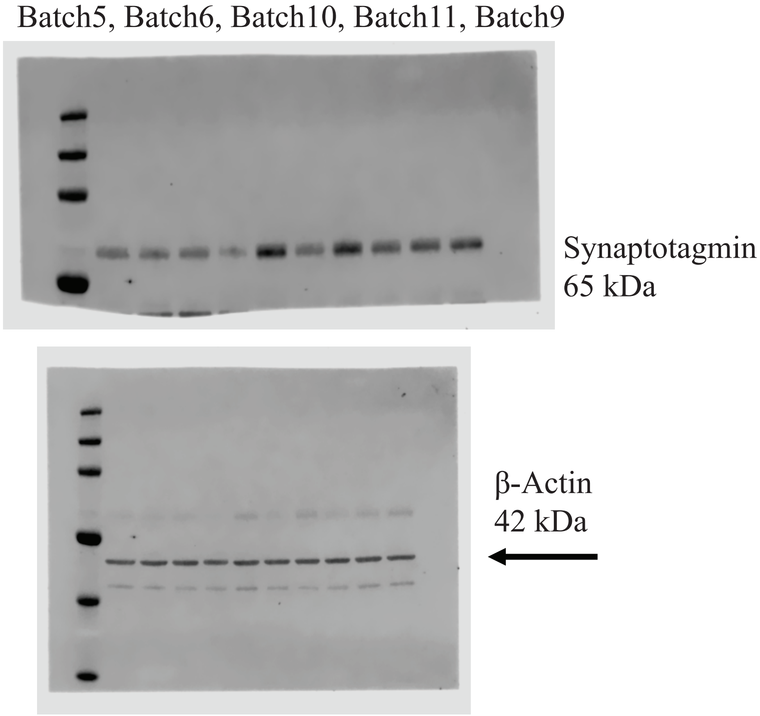

Fig. 7e (Batch10)

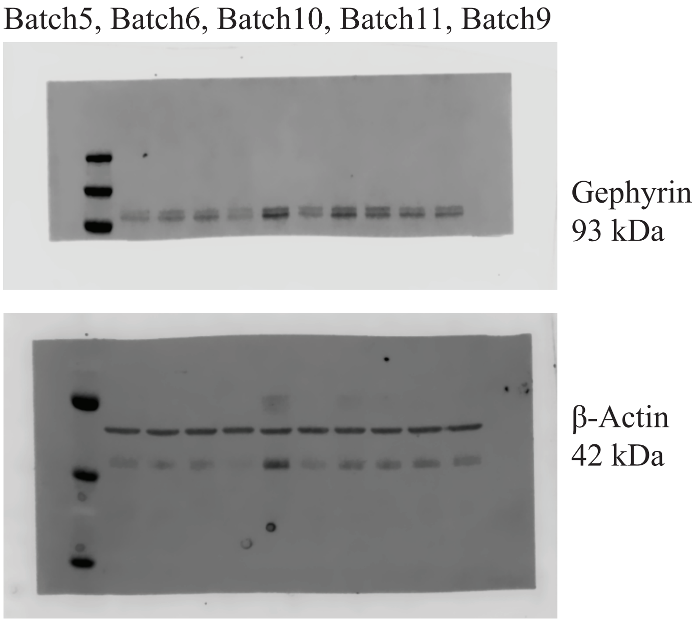

Fig. 7f (Batch11)

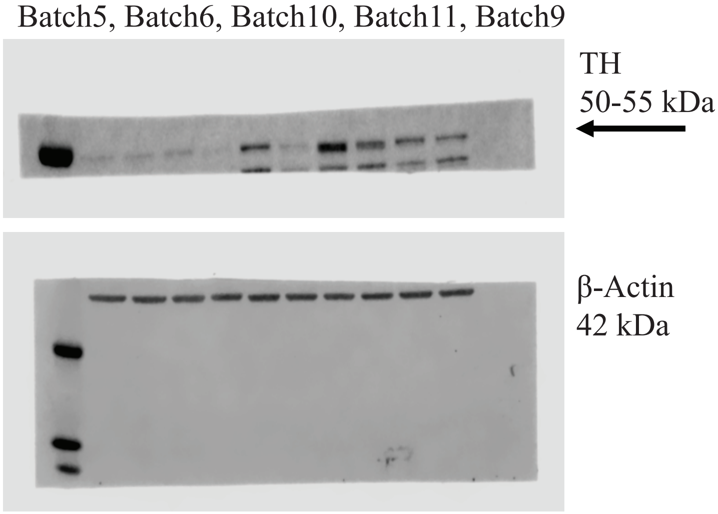

Supplementary Figure 17: Uncropped western blot images shown in Figure 6 and Figure 7.

SFig. 3d (First 7 bands from left to right)

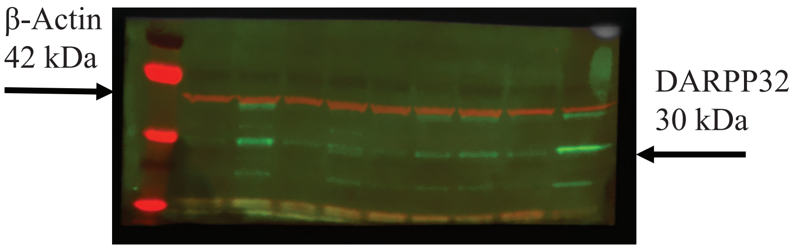

SFig. 3e (First 7 bands from left to right)

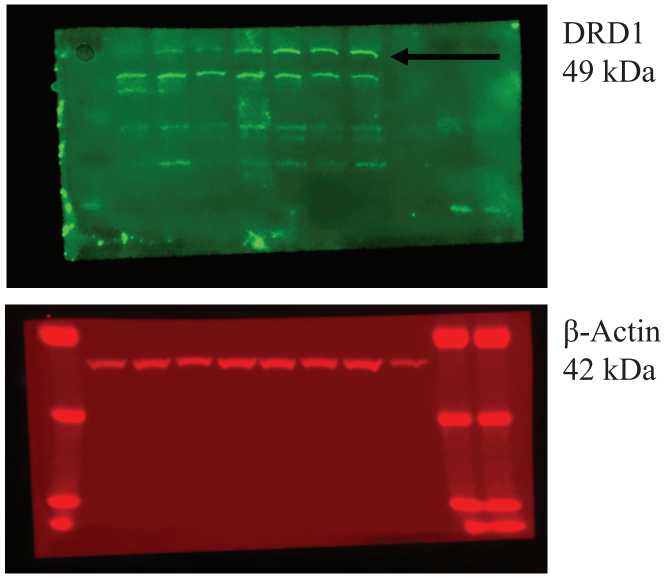

SFig. 3f (First 7 bands from left to right)

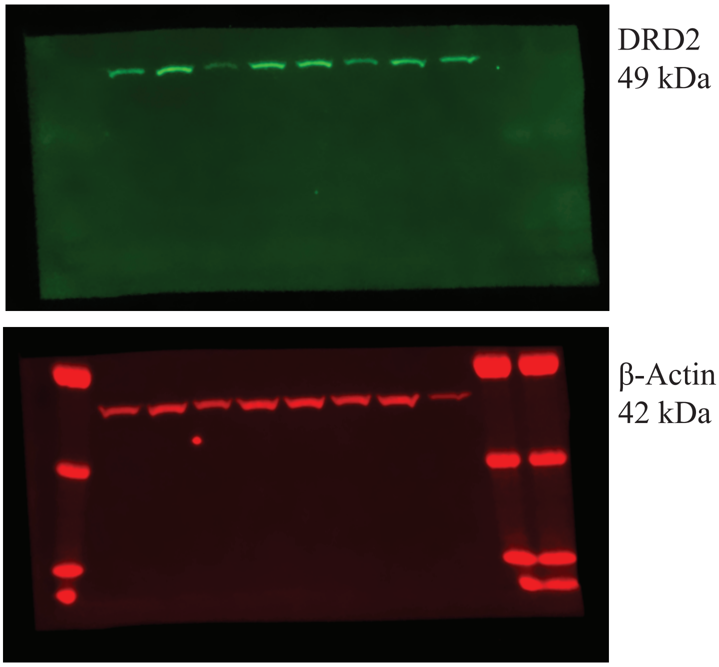

SFig. 3g (First 7 bands from left to right)

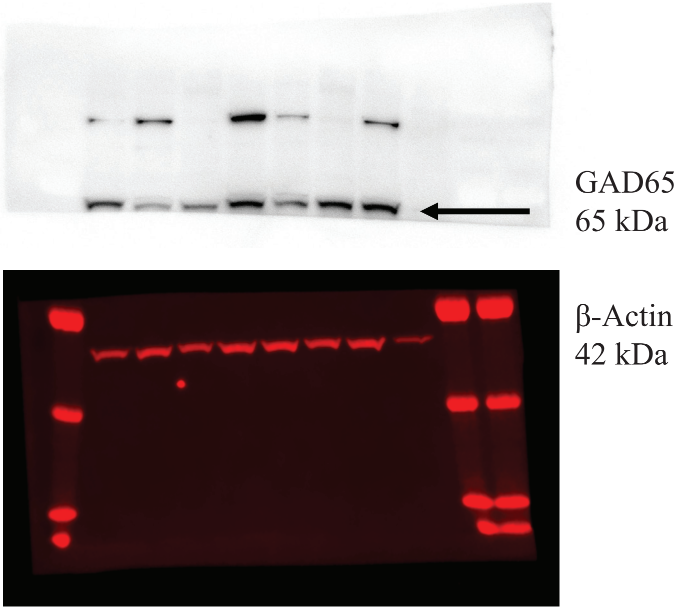

SFig. 12d (Last 4 bands from left to right)

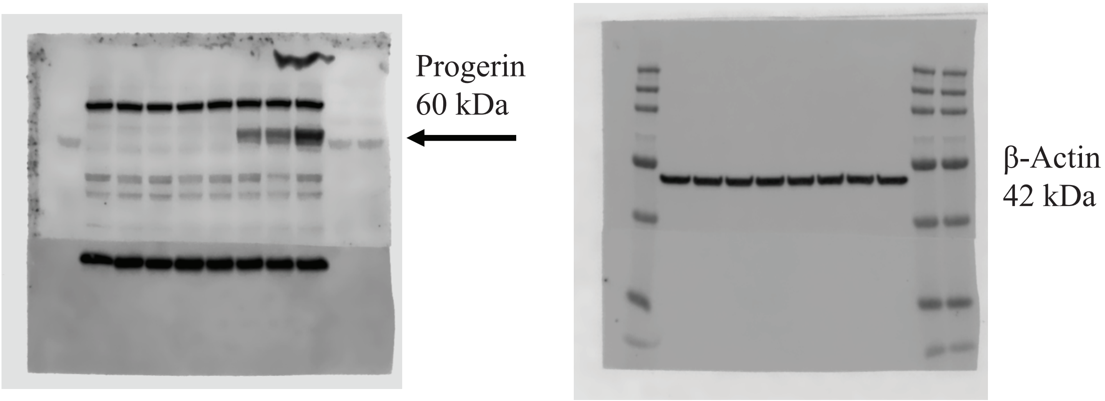

Supplementary Figure 18: Uncropped western blot images shown in Supplementary Figure 3 and Supplementary Figure 12d. The membrane of the blot in SFig. 12d was cut and then reassembled for image acquisition. This process accounts for the color discrepancy observed between the upper and lower portions of the blot.

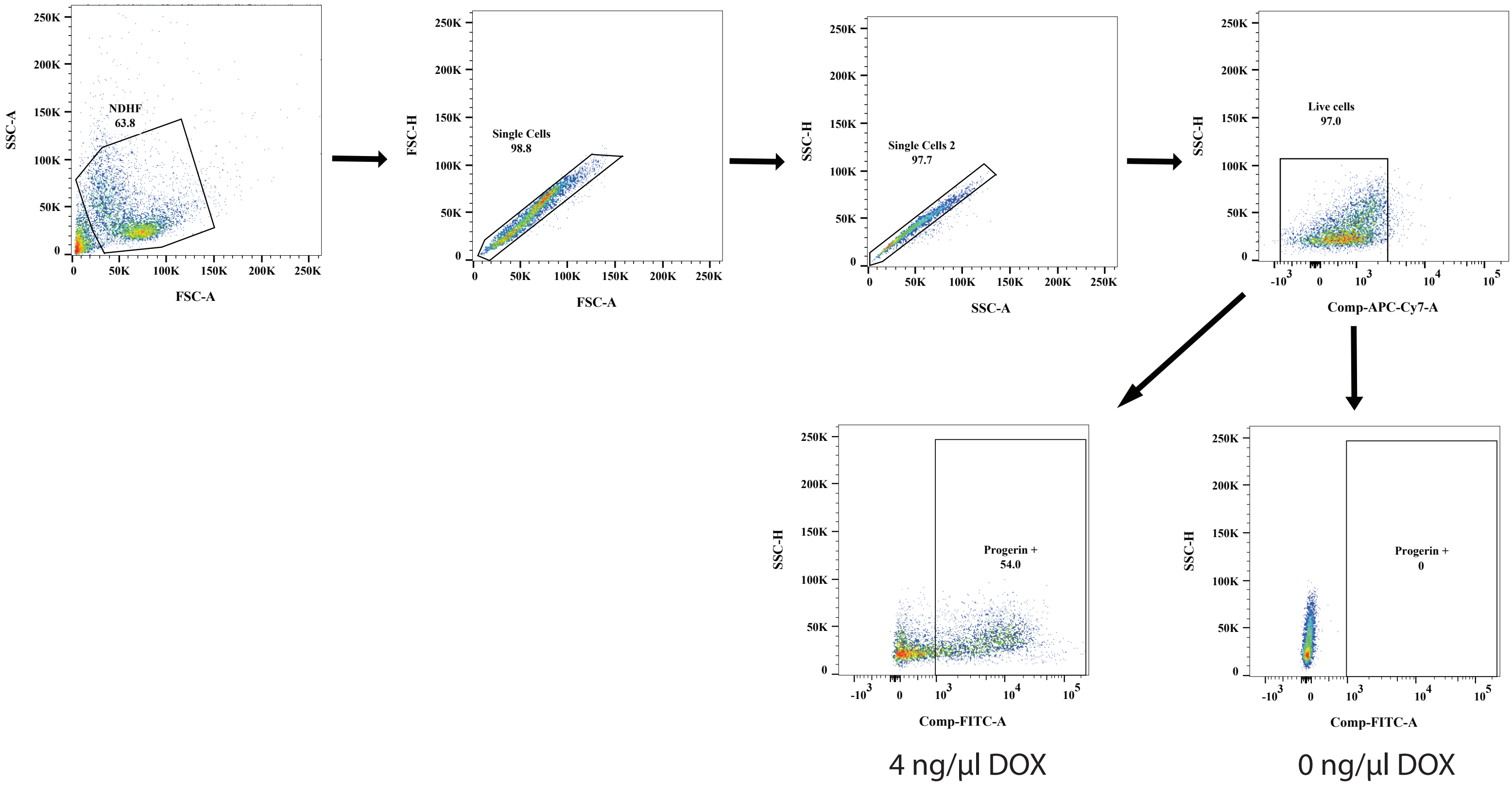

Supplementary Figure 19: Gating strategy for the flow cytometry experiment shown in Supplementary Figure 12. Doxycycline treatment leads to a clear population of Progerin (FITC signal) positive cells.

Supplementary Table 1: Cell lines used in the study.

**Lab identifier**	**Name**	**Sex**	**Age of sampling**	**Source**	**Reference Name**	**Karyotype**
201	WT 1	F	-	GIBCO	A13777	Normal
200	WT 2	F	83	Reinhardt *et al.*, 2013	K7.1	Chr1-mosaic gain q21.2<br>Chr1- gain q25.3
389	WT	M	6	Promocell	NHDF	Chr20q - gain
390	Progerin	M	6	Innsbruck, Prof. Frank Edenhover	NHDF progerin	Chr20q - gain

Supplementary Table 2: Primary and secondary antibodies used in Western blots.

Antibody	Source	Ref.no.	RRID	Species	Dilution
β-Actin	Cell Signaling	3700S	*AB_2242334*	Mouse	1:20000
LAMINB1	Abcam	ab16048	*AB_443298*	Rabbit	1:500
LMNA	Sigma	L1293	*AB_532254*	Rabbit	1:500
DRD1	Abcam	ab216644	*AB_2941932*	Rabbit	1:300
DRD2	Abcam	ab85367	*AB_10674739*	Rabbit	1:300
Histone H3	Millipore	05-1341	*AB_1977240*	Mouse	1:20000
TUJ1	BioLegend	801201	*AB_2313773*	Mouse	1:20000
TAU	Abcam	ab80579	*AB_1603723*	Mouse	1:1000
Synaptotagmin1	Synaptic Systems	105011	*AB_2619761*	Mouse	1:200
VAMP2	Abcam	ab215721	*AB_2923382*	Rabbit	1:1000
TH	Abcam	ab112	*AB_297840*	Rabbit	1:600
DARPP32	Abcam	ab40801	*AB_731843*	Rabbit	1:400
GAD65	R&D systems	AF2247	*AB_2108039*	Goat	1:1000
ECL anti-goat	Santa Cruz	sc-2020	*AB_631728*	Donkey	1:1000
Anti-rabbit H+L 800	Cell Signaling	5151	*AB_10697505*	Goat	1:10000
Anti-mouse H+L 680	Cell Signaling	5470	*AB_10696895*	Goat	1:10000

Supplementary Table 3: Primary and secondary antibodies used in immunofluorescence stainings.

Antibody	Source	Cat.no.	RRID	Species	Dilution
NESTIN	BD Bioscience	611659	*AB_399177*	Mouse	1:600
PAX6	Biolegend	901302	*AB_2749901*	Rabbit	1:300
FOXA2	Santa Cruz	sc-101060	*AB_1124660*	Mouse	1:100
MASH1/ASCL1	BD Bioscience	556604	*AB_396479*	Mouse	1:200
CORIN	R&D systems	MAB2209	*AB_2082224*	Rat	1:200
TRA-1-60	Millipore	MAB4360	*AB_2119183*	Mouse	1:50
SSEA-4	Millipore	MAB4304	*AB_177629*	Mouse	1:50
NANOG	Millipore	AB5731	*AB_2267042*	Rabbit	1:200
OCT4	Abcam	ab19857	*AB_445175*	Rabbit	1:400
CTIP2	Abcam	ab18465	*AB_2064130*	Rat	1:300
TH	Abcam	ab112	*AB_297840*	Rabbit	1:600
MAP2	Abcam	ab92434	*AB_2138147*	Chicken	1:1000
SOX1	R&D systems	AF3369	*AB_2239879*	Goat	1:100
SOX2	Abcam	ab97959	*AB_2341193*	Rabbit	1:200
SOX2	R&D Systems	AF2018	*AB_355110*	Goat	1:200
DARPP32	Abcam	ab40801	*AB_731843*	Rabbit	1:400
P21 Waf1/Cip1	Cell Signaling	2946	*AB_2260325*	Mouse	1:200
P16INK4a	Abcam	ab108349	*AB_10858268*	Rabbit	1:200
P53	Thermo Fisher Scientific	MA5-12557	*AB_10989883*	Mouse	1:200

H2AX	Millipore	05-636-I	*AB_2755003*	Mouse	1:200
53BP1	Novus	NB100-304	*AB_10003037*	Rabbit	1:200
Anti-mouse 488	Invitrogen	A21202	*AB_141607*	Donkey	1:1000
Anti-mouse 568	Invitrogen	A10037	*AB_2534013*	Donkey	1:1000
Anti-goat 657	Invitrogen	A21447	*AB_2535864*	Donkey	1:1000
Anti-rabbit 647	Invitrogen	A31573	*AB_2536183*	Donkey	1:1000
Anti-chicken 568	Invitrogen	A11041	*AB_2534098*	Goat	1:1000
Anti-chicken 647	Invitrogen	A21449	*AB_2535866*	Goat	1:1000
Anti-mouse 488	Invitrogen	A11029	*AB_2534088*	Goat	1:1000
Anti-mouse 568	Invitrogen	A11031	*AB_144696*	Goat	1:1000
Anti-mouse 647	Invitrogen	A21236	*AB_2535805*	Goat	1:1000
Anti-rabbit 568	Invitrogen	A11036	*AB_10563566*	Goat	1:1000
Anti-rabbit 647	Invitrogen	A21244	*AB_2535812*	Goat	1:1000

Supplementary Table 4: Primers used in qPCRs for the striatum organoids characterization.

Gene	Forward Primer	Reverse Primer
DARPP32	CCTGAAGGTCATCAGGCAGT	GGTCTTCCACTTGGTCCTCA
DRD1	AGGGACATGTCTTTGGCTTCAG	GGGAACAGTGTTAGCACCTGTT
DRD2	CTGAGGGCTCCACTAAAGGAG	CATTCTTCTCTGGTTTGGCG
CTIP2	ATCCTCAGCCCCTTTTGTTT	GCCGTTGTTCCTGAATTGTT
ASCL1	GTCCTGTCGCCCACCATCTC	CCCTCCCAACGCCACTGAC
FOXG1	TGTTGACTCAGAACTCGCTGG	CTGCTCTGCGAAGTCATTGAC
FOXP1	GCAGTTACAGCAGCAGCACCTCC	CAGCCTGGCCACTTGCATACACC
FOXP2	AATGTGGGAGCCATACGAAG	GCCTGCCTTATGAGAGTTGC
OTX2	TCAACTTGCCCGAGTCGAGG	CAATGGTCGGGACTGAGGTG
NKX1.2	CGCATCCAATCTCAAGGAAT	TGTGCCCAGAGTGAAGTTTG
GSX2	ATGTCGCGCTCCTTCTATGTC	ATGCCAAGCGGGATGAAGAAA
ACTINB	TCAAGATCATTGCTCCTCCTGAG	ACATCTGCTGGAAGGTGGACA

Supplementary Table 5: Genes related to cellular and oxidative stress.

Genes	Role in Cellular/Oxidative stress	References
PGK1 (Phosphoglycerate Kinase 1)	A protein kinase responsible for governing mitochondrial activity and cell stress-triggered autophagy. Downregulation has been linked to oxidative stress inhibition.	[1,2]
ARCN1 (Archain 1)	Involvement in ER stress response.	[2,3]
GORASP2 (Golgi Reassembly Stacking Protein 2)	Involvement in ER stress response.	[2,4]
TFAM (Transcription Factor A, Mitochondrial)	Stress response induced by mitochondria dysfunction.	[5]
HMGB1 (High Mobility Group Box 1)	Cytokine with a role in initiating neuroinflammation.	[6]
HMGN1 (High Mobility Group Nucleosome Binding Domain 1)	Chromatin architectural protein. Can contribute to neuroinflammation.	[7,8]
SAP130 (Sin3A Associated Protein 130)	It is increased in neuroinflammation.	[9]
CALR (Calreticulin)	ER Ca2+ binding protein. Is activated by environmental stress.	[10,11]
PPIA (Peptidylprolyl Isomerase A)	It has a role as molecular chaperone. Increased levels in ALS.	[12]
FN1 (Fibronectin 1)	Extracellular matrix glycoprotein. Increased expression in cellular stress response.	[13]
IL33 (Interleukin 33)	Cytokine with a role in neuroinflammation.	[14,15]
HSPA5 (Heat Shock Protein Family A (Hsp70) Member 5)	Upregulated under ER stress for the clearing of misfolded proteins.	[16]
LGALS1 (Galectin 1)	Highly expressed during neuroinflammation.	[17,18]
GSTP1 (Glutathione S−Tranferase)	Increased levels have been associated with cancer and neurodegenerative diseases.	[19]
MGST3 (microsomal Glutathione S−Tranferase)	Possible role in neuroinflammation, with the production of LCT4 for the biosynthesis of proinflammatory LTs.	[20]
PRDX1 (Peroxiredoxin 1)	An antioxidant enzyme that protects the brain against oxidative stress. Increased levels have been found in AD patients.	[21,22]
ATM (ATM Serine/Threonine Kinase)	It is activated by oxidative stress and is important for the repair of DNA damage.	[23,24]
PRDX5 (Peroxiredoxin 5)	Antioxidant enzyme, protecting cells against oxidative stress (similar to PRDX1).	[25,26]
SOD1 (Superoxide Dismutase 1)	It is oxidative stress dependent and is upregulated during brain inflammation.	[27,28]
GPX4 (Glutathione Peroxidase 4)	It is overexpressed during ferroptosis and neuroinflammation.	[29]

Supplementary Table 6: Significantly dysregulated genes with common expression pattern in Progerin-expressing assembloids and post-mortem human brain.

Genes	Implication in brain aging and/or neurodegeneration
VIP, PNCO	Their downregulation is associated with inhibitory neurotransmission in GABAergic neurons and they are both significantly downregulated in human aged brains[30].
SST, CRH	Their downregulation has been found in aging brain but also in AD genetic signature[30–32].
PNOC	Its downregulation correlates best with the biological age of the human brain post-mortem data[33].
NRGN	Its downregulation correlates with higher density of amyloid plaques in post mortem brains of AD patients[34].
NETO1	It is essential for the regulation of the connectivity of glutamatergic neurons[35,36] and its downregulation could cause dysfunction in synaptic circuits.
KCNAB1, GABRA4	Both have a positive co-expression with *BDNF* which shows gradual downregulation in aging human prefrontal cortex[37].
IER3	It has been implicated in cellular stress response and it is upregulated in inflammatory conditions[38]
GRM4	It has been found to be reduced in the prelimbic of aged rats[39].
GMPR	Its upregulation correlates best with the biological age of the human brain post-mortem data[33].
DRD1, DRD2	Both receptors are crucial for dopamine signalling in the striatum, and their reduced expression can be linked to motor and cognitive abnormalities[40].
DLX6	It encodes an important transcription factor for the regulation of GABAergic neurons[41] and therefore its downregulation could be associated with aging.
COL21A1	Its upregulation has been correlated with Alzheimer's disease (AD)[42].
COBL	It has be linked with reduced dendrite arborisation[43].
CHI3L1	It has been linked to aging and neurodegeneration[44,45].
CHCHD2	Its downregulation could be associated with the aging brain and development of PD, as studies have shown that mutations in this gene are tied to dysfunctional mitochondria[46,47], while at the same time reduction of *CHCHD2* mRNA levels has been found in erythrocytes of PD patients[48].

Supplementary References

1. Xu, M. *et al.* Evodiamine prevents traumatic brain injury through inhibiting oxidative stress via PGK1/NRF2 pathway. *Biomed. Pharmacother.* **153**, 113435 (2022).

2. Bhaduri, A. *et al.* Cell stress in cortical organoids impairs molecular subtype specification. *Nature* **578**, 142–148 (2020).

3. Izumi, K. *et al.* ARCN1 Mutations Cause a Recognizable Craniofacial Syndrome Due to COPI-Mediated Transport Defects. *Am. J. Hum. Genet.* **99**, 451–459 (2016).

4. Zhang, X. & Wang, Y. The Golgi stacking protein GORASP2/GRASP55 serves as an energy sensor to promote autophagosome maturation under glucose starvation. *Autophagy* **14**, 1649–1651 (2018).

5. Hunt, R. J. *et al.* Mitochondrial stress causes neuronal dysfunction via an ATF4-dependent increase in L-2-hydroxyglutarate. *J. Cell Biol.* **218**, 4007–4016 (2019).

6. Paudel, Y. N., Angelopoulou, E., Piperi, C., Othman, I. & Shaikh, M. F. Hmgb1-mediated neuroinflammatory responses in brain injuries: Potential mechanisms and therapeutic opportunities. *Int. J. Mol. Sci.* **21**, 1–29 (2020).

7. Furusawa, T. & Cherukuri, S. Developmental function of HMGN proteins. *Biochim. Biophys. Acta - Gene Regul. Mech.* **1799**, 69–73 (2010).

8. Farley, S. J., Grishok, A. & Zeldich, E. Shaking up the silence: consequences of HMGN1 antagonizing PRC2 in the Down syndrome brain. *Epigenetics and Chromatin* **15**, 1–27 (2022).

9. Wang, Y. *et al.* Microglial Mincle receptor in the PVN contributes to sympathetic hyperactivity in acute myocardial infarction rat. *J. Cell. Mol. Med.* **23**, 112–125 (2019).

10. Chen, C. J. *et al.* Calreticulin Expression Controls Cellular Redox, Stemness, and Radiosensitivity to Function as a Novel Adjuvant for Radiotherapy in Neuroblastoma. *Oxid. Med. Cell. Longev.* **2023**, (2023).

11. Michalak, M. Calreticulin: Endoplasmic reticulum Ca2+ gatekeeper. *J. Cell. Mol. Med.* 1–19 (2023) doi:10.1111/jcmm.17839.

12. Lauranzano, E. *et al.* Peptidylprolyl isomerase A governs TARDBP function and assembly in heterogeneous nuclear ribonucleoprotein complexes. *Brain* **138**, 974–991 (2015).

13. Dhanani, K. C. H., Samson, W. J. & Edkins, A. L. Fibronectin is a stress responsive gene regulated by HSF1 in response to geldanamycin. *Sci. Rep.* **7**, (2017).

14. Zharichenko, N. & Njoku, D. B. The role of pro-inflammatory and regulatory signaling by il-33 in the brain and liver: A focused systematic review of mouse and human data and risk of bias assessment of the literature. *Int. J. Mol. Sci.* **21**, (2020).

15. Sun, Y. *et al.* Therapeutic Opportunities of Interleukin-33 in the Central Nervous System. *Front. Immunol.* **12**, 1–10 (2021).

16. Nowakowska, M. *et al.* Profiling the Expression of Endoplasmic Reticulum Stress Associated Heat Shock Proteins in Animal Epilepsy Models. *Neuroscience* **429**, 156–172 (2020).

17. Wang, J. *et al.* Galectin-1-secreting neural stem cells elicit long-term neuroprotection against ischemic brain injury. *Sci. Rep.* **5**, (2015).

18. Aalinkeel, R. & Mahajan, S. D. Neuroprotective role of galectin-1 in central nervous system pathophysiology. *Neural Regen. Res.* **11**, 896–897 (2016).

19. Allocati, N., Masulli, M., Di Ilio, C. & Federici, L. Glutathione transferases: Substrates, inihibitors and pro-drugs in cancer and neurodegenerative diseases. *Oncogenesis* **7**, (2018).

20. Fetissov, S. O. *et al.* Expression of microsomal glutathione S-transferase type 3 mRNA in the rat nervous system. *Neuroscience* **115**, 891–897 (2002).

21. Kim, S. *et al.* The antioxidant enzyme Peroxiredoxin-1 controls stroke-associated microglia against acute ischemic stroke. *Redox Biol.* **54**, 102347 (2022).

22. Szeliga, M. Peroxiredoxins in neurodegenerative diseases. *Antioxidants* **9**, 1–19 (2020).

23. Kozlov, S. V. *et al.* Reactive oxygen species (ROS)-activated ATM-dependent phosphorylation of cytoplasmic substrates identified by large-scale phosphoproteomics screen. *Mol. Cell. Proteomics* **15**, 1032–1047 (2016).

24. Berger, N. D., Stanley, F. K. T., Moore, S. & Goodarzi, A. A. ATM-dependent pathways of chromatin remodelling and oxidative DNA damage responses. *Philos. Trans. R. Soc. B Biol. Sci.* **372**, (2017).

25. Tavleeva, M. M. *et al.* Effects of Antioxidant Gene Overexpression on Stress Resistance and Malignization In Vitro and In Vivo: A Review. *Antioxidants* **11**, 1–25 (2022).

26. Yuan, J. *et al.* Overexpression of antioxidant enzyme peroxiredoxin 5 protects human tendon cells against apoptosis and loss of cellular function during oxidative stress. *Biochim. Biophys. Acta - Mol. Cell Res.* **1693**, 37–45 (2004).

27. Dimayuga, F. O. *et al.* SOD1 overexpression alters ROS production and reduces neurotoxic inflammatory signaling in microglial cells. *J. Neuroimmunol.* **182**, 89–99 (2007).

28. Dell'Orco, M. *et al.* HuD regulates SOD1 expression during oxidative stress in differentiated neuroblastoma cells and sporadic ALS motor cortex. *Neurobiol. Dis.* **148**, 105211 (2021).

29. Fang, J. *et al.* Overexpression of GPX4 attenuates cognitive dysfunction through inhibiting hippocampus ferroptosis and neuroinflammation after traumatic brain injury. *Free Radic. Biol. Med.* **204**, 68–81 (2023).

30. Loerch, P. M. *et al.* Evolution of the aging brain transcriptome and synaptic regulation. *PLoS One* **3**, (2008).

31. Peng, S. *et al.* Transcriptomic Changes Highly Similar to Alzheimer's Disease Are Observed in a Subpopulation of Individuals During Normal Brain Aging. *Front. Aging Neurosci.* **13**, 1–16 (2021).

32. Berchtold, N. C. *et al.* Synaptic genes are extensively downregulated across multiple brain regions in normal human aging and Alzheimer ' s disease. *Neurobiol. Aging* **34**, 1653–1661 (2013).

33. González-Velasco, O., Papy-García, D., Le Douaron, G., Sánchez-Santos, J. M. & De Las Rivas, J. Transcriptomic landscape, gene signatures and regulatory profile of aging in the human brain. *Biochim. Biophys. Acta - Gene Regul. Mech.* **1863**, 194491 (2020).

34. Sun, X. *et al.* Association of neurogranin gene expression with Alzheimer ' s disease pathology in the perirhinal cortex. 1–9 (2021) doi:10.1002/trc2.12162.

35. Orav, E. *et al.* NETO1 guides development of glutamatergic connectivity in the hippocampus by

regulating axonal kainate receptors. *eNeuro* **4**, (2017).

36. Straub, C. *et al.* Distinct functions of kainate receptors in the brain are determined by the auxiliary subunit Neto1. *Nat. Neurosci.* **14**, 866–873 (2011).

37. Oh, H., Lewis, D. A. & Sibille, E. The Role of BDNF in Age-Dependent Changes of Excitatory and Inhibitory Synaptic Markers in the Human Prefrontal Cortex. **2**, 3080–3091 (2016).

38. Arlt, A. & Schäfer, H. Role of the immediate early response 3 (IER3) gene in cellular stress response, inflammation and tumorigenesis. *Eur. J. Cell Biol.* **90**, 545–552 (2011).

39. Hernandez, C. M. *et al.* Age-Related Declines in Prefrontal Cortical Expression of Metabotropic Glutamate Receptors that Support Working Memory. **5**, (2018).

40. Hemby, S. E., Trojanowski, J. Q. & Ginsberg, S. D. Neuron-specific age-related decreases in dopamine receptor subtype mRNAs. *J. Comp. Neurol.* **456**, 176–183 (2003).

41. Lombares, C. De *et al.* Dlx5 and Dlx6 expression in GABAergic neurons controls behavior , metabolism , healthy aging and lifespan. **11**, 6638–6656 (2019).

42. Kong, W. *et al.* Molecular Neurodegeneration Independent component analysis of Alzheimer's DNA microarray gene expression data. (2009) doi:10.1186/1750-1326-4-5.

43. Ahuja, R. *et al.* Cordon-Bleu Is an Actin Nucleation Factor and Controls Neuronal Morphology. *Cell* **131**, 337–350 (2007).

44. Moreno-Rodriguez, M., Perez, S. E., Nadeem, M., Malek-Ahmadi, M. & Mufson, E. J. Frontal cortex chitinase and pentraxin neuroinflammatory alterations during the progression of Alzheimer's disease. *J. Neuroinflammation* **17**, 1–15 (2020).

45. Sanfilippo, C. *et al.* Sex difference in CHI3L1 expression levels in human brain aging and in Alzheimer's disease. *Brain Res.* **1720**, 146305 (2019).

46. Kee, T. R. *et al.* Mitochondrial CHCHD2 : Disease-Associated Mutations , Physiological Functions , and Current Animal Models. **13**, 1–15 (2021).

47. Meng, H. *et al.* Loss of Parkinson’s disease-associated protein CHCHD2 affects mitochondrial crista structure and destabilizes cytochrome c. *Nat. Commun.* **8**, 1–18 (2017).

48. Liu, X. *et al.* Reduced erythrocytic CHCHD2 mRNA is associated with brain pathology of Parkinson ' s disease. *Acta Neuropathol. Commun.* 1–16 (2021) doi:10.1186/s40478-021-01133-6.
