# Supplementary material for: Modeling early phenotypes of Parkinson’s disease by age-induced midbrain-striatum assembloids
Source: Commun Biol. 2024 Nov 23;7:1561. doi: 10.1038/s42003-024-07273-4 (PMC11585662; doi:10.1038/s42003-024-07273-4)
Supplement: Supplementary file 2 — Description of Additional Supplementary File [file 42003_2024_7273_MOESM2_ESM.pdf]

Description Of Additional Supplementary File

File name: Supplementary Data 1
Description: Common downregulated significantly differentially expressed genes between progerin overexpressing assembloids and aged male brains from Berchtold et al., 2008.

File name: Supplementary Data 2
Description: Common upregulated significantly differentially expressed genes between progerin overexpressing assembloids and aged male brains from Berchtold et al., 2008.

File name: Supplementary Data 3
Description: Common downregulated significantly differentially expressed genes between progerin overexpressing assembloids and aged brain data from González-Velasco et al., 2020.

File name: Supplementary Data 4
Description: Common upregulated significantly differentially expressed genes between progerin overexpressing assembloids and aged brain data from González-Velasco et al., 2020.
