# Supplementary material for: Modeling early phenotypes of Parkinson’s disease by age-induced midbrain-striatum assembloids
Source: Commun Biol. 2024 Nov 23;7:1561. doi: 10.1038/s42003-024-07273-4 (PMC11585662; doi:10.1038/s42003-024-07273-4)
Supplement: Supplementary file 7 — Reporting Summary [file 42003_2024_7273_MOESM7_ESM.pdf]

nature portfolio

Corresponding author(s): Jens Christian Schwamborn

Last updated by author(s): Oct 20, 2024

Reporting Summary

Nature Portfolio wishes to improve the reproducibility of the work that we publish. This form provides structure for consistency and transparency in reporting. For further information on Nature Portfolio policies, see our Editorial Policies and the Editorial Policy Checklist.

Statistics

For all statistical analyses, confirm that the following items are present in the figure legend, table legend, main text, or Methods section.

n/a	Confirmed	
☐	☒	The exact sample size (*n*) for each experimental group/condition, given as a discrete number and unit of measurement
☐	☒	A statement on whether measurements were taken from distinct samples or whether the same sample was measured repeatedly
☐	☒	The statistical test(s) used AND whether they are one- or two-sided<br>*Only common tests should be described solely by name; describe more complex techniques in the Methods section.*
☒	☐	A description of all covariates tested
☐	☒	A description of any assumptions or corrections, such as tests of normality and adjustment for multiple comparisons
☒	☐	A full description of the statistical parameters including central tendency (e.g. means) or other basic estimates (e.g. regression coefficient) AND variation (e.g. standard deviation) or associated estimates of uncertainty (e.g. confidence intervals)
☒	☐	For null hypothesis testing, the test statistic (e.g. *F*, *t*, *r*) with confidence intervals, effect sizes, degrees of freedom and *P* value noted<br>*Give P values as exact values whenever suitable.*
☒	☐	For Bayesian analysis, information on the choice of priors and Markov chain Monte Carlo settings
☒	☐	For hierarchical and complex designs, identification of the appropriate level for tests and full reporting of outcomes
☒	☐	Estimates of effect sizes (e.g. Cohen's *d*, Pearson's *r*), indicating how they were calculated

Our web collection on statistics for biologists contains articles on many of the points above.

Software and code

Policy information about availability of computer code

Data collection	N/A
Data analysis	Custom code using MATLAB (2021a, Mathworks, RRID:SCR_001622) for image analysis, data analysis with GraphPad Prism 9.0.0 and R studio (23.06.1+504 version) with R 4.2.2 version.

For manuscripts utilizing custom algorithms or software that are central to the research but not yet described in published literature, software must be made available to editors and reviewers. We strongly encourage code deposition in a community repository (e.g. GitHub). See the Nature Portfolio guidelines for submitting code & software for further information.

Data

Policy information about availability of data

All manuscripts must include a data availability statement. This statement should provide the following information, where applicable:

- Accession codes, unique identifiers, or web links for publicly available datasets
- A description of any restrictions on data availability
- For clinical datasets or third party data, please ensure that the statement adheres to our policy

Raw and processed data that support the findings in this study, as well as scripts used for the analysis of the data are publicly available at this link: https://doi.org/10.17881/4va5-e156

Bulk RNA and single nuclei RNA sequencing data are available on Gene Expression Omnibus (GEO) under the accession codes GSE236458 and GSE241632 respectively.

Research involving human participants, their data, or biological material

Policy information about studies with human participants or human data. See also policy information about sex, gender (identity/presentation), and sexual orientation and race, ethnicity and racism.

Reporting on sex and gender	In this study we used human induced pluripotent stem cell and derived neuroepithelial stem cells from 2 female and 1 male healty individuals. We also used the isogenic induced pluripotent stem cell line with the introduced progerin transgene under the tet-on system from the male healthy individual.
Reporting on race, ethnicity, or other socially relevant groupings	*Please specify the socially constructed or socially relevant categorization variable(s) used in your manuscript and explain why they were used. Please note that such variables should not be used as proxies for other socially constructed/relevant variables (for example, race or ethnicity should not be used as a proxy for socioeconomic status). Provide clear definitions of the relevant terms used, how they were provided (by the participants/respondents, the researchers, or third parties), and the method(s) used to classify people into the different categories (e.g. self-report, census or administrative data, social media data, etc.) Please provide details about how you controlled for confounding variables in your analyses.*
Population characteristics	*Describe the covariate-relevant population characteristics of the human research participants (e.g. age, genotypic information, past and current diagnosis and treatment categories). If you filled out the behavioural & social sciences study design questions and have nothing to add here, write "See above."*
Recruitment	*Describe how participants were recruited. Outline any potential self-selection bias or other biases that may be present and how these are likely to impact results.*
Ethics oversight	*Identify the organization(s) that approved the study protocol.*

Note that full information on the approval of the study protocol must also be provided in the manuscript.

Field-specific reporting

Please select the one below that is the best fit for your research. If you are not sure, read the appropriate sections before making your selection.

☒ Life sciences ☐ Behavioural & social sciences ☐ Ecological, evolutionary & environmental sciences

For a reference copy of the document with all sections, see nature.com/documents/nr-reporting-summary-flat.pdf

Life sciences study design

All studies must disclose on these points even when the disclosure is negative.

Sample size	Sample size was determined by the availability of the cell lines. In our case 2 independent cell lines were used for the characterisation of the line, and one progerin expressing cell line with its isogenic wild type control for the aging assessment in the model.
Data exclusions	Data were excluded based on the outlier used. In the data plotted with GraphPad Prism, the ROUT method Q 1% outlier test was used. In the data plotted with R, outlier removal was performed based on the Inter-Quartile Range (IQR) proximity rule. This information is available in the figure legend of each plot.
Replication	For the single-nuclei RNA sequencing experiment, one experiment was run using polled organoids or assembloids from 2 independent derivations for each cell line. For the bulk RNA sequencing experiment, assembloids from 3 independent derivations were sequenced. For all the other experiments, at least 3 batches (independent derivations of organoids) were used.
Randomization	Specific lines were used for the whole study. The selection the two wild type lines used for the optimisation of the model generation was random and these two lines was used for the whole study. The progerin engineered cell line was compared to its isogenic wild type control line.
Blinding	Blinding was not applicable in our experiments.

Reporting for specific materials, systems and methods

We require information from authors about some types of materials, experimental systems and methods used in many studies. Here, indicate whether each material, system or method listed is relevant to your study. If you are not sure if a list item applies to your research, read the appropriate section before selecting a response.

Materials & experimental systems

n/a	Involved in the study
☐	☒ Antibodies
☐	☒ Eukaryotic cell lines
☒	☐ Palaeontology and archaeology
☒	☐ Animals and other organisms
☒	☐ Clinical data
☒	☐ Dual use research of concern
☒	☐ Plants

Methods

n/a	Involved in the study
☒	☐ ChIP-seq
☐	☒ Flow cytometry
☒	☐ MRI-based neuroimaging

Antibodies

Antibodies used

Detailed description of the Antibodies used can be found in the Supplementary Table 2 and 3.
β-Actin Cell Signaling 3700S AB_2242334 Mouse 1:20000
LAMINB1 Abcam ab16048 AB_443298 Rabbit 1:500
LMNA Sigma L1293 AB_532254 Rabbit 1:500
DRD1 Abcam ab216644 AB_2941932 Rabbit 1:300
DRD2 Abcam ab85367 AB_10674739 Rabbit 1:300
Histone H3 Millipore 05-1341 AB_1977240 Mouse 1:20000
TUJ1 BioLegend 801201 AB_2313773 Mouse 1:20000
TAU Abcam ab80579 AB_1603723 Mouse 1:1000
Synaptotagmin1 Synaptic Systems 105011 AB_2619761 Mouse 1:200
VAMP2 Abcam ab215721 AB_2923382 Rabbit 1:1000
TH Abcam ab112 AB_297840 Rabbit 1:600
DARPP32 Abcam ab40801 AB_731843 Rabbit 1:400
GAD65 R&D systems AF2247 AB_2108039 Goat 1:1000
ECL anti-goat Santa Cruz sc-2020 AB_631728 Donkey 1:1000
Anti-rabbit H+L 800 Cell Signaling 5151 AB_10697505 Goat 1:10000
Anti-mouse H+L 680 Cell Signaling 5470 AB_10696895 Goat 1:10000
NESTIN BD Bioscience 611659 AB_399177 Mouse 1:600
PAX6 Biolegend 901302 AB_2749901 Rabbit 1:300
FOXA2 Santa Cruz sc-101060 AB_1124660 Mouse 1:100
MASH1/ASCL1 BD Bioscience 556604 AB_396479 Mouse 1:200
CORIN R&D systems MAB2209 AB_2082224 Rat 1:200
TRA-1-60 Millipore MAB4360 AB_2119183 Mouse 1:50
SSEA-4 Millipore MAB4304 AB_177629 Mouse 1:50
NANOG Millipore AB5731 AB_2267042 Rabbit 1:200
OCT4 Abcam ab19857 AB_445175 Rabbit 1:400
CTIP2 Abcam ab18465 AB_2064130 Rat 1:300
TH Abcam ab112 AB_297840 Rabbit 1:600
MAP2 Abcam ab92434 AB_2138147 Chicken 1:1000
SOX1 R&D systems AF3369 AB_2239879 Goat 1:100
SOX2 Abcam ab97959 AB_2341193 Rabbit 1:200
SOX2 R&D Systems AF2018 AB_355110 Goat 1:200
DARPP32 Abcam ab40801 AB_731843 Rabbit 1:400
P21 Waf1/Cip1 Cell Signaling 2946 AB_2260325 Mouse 1:200
P16INK4a Abcam ab108349 AB_10858268 Rabbit 1:200
P53 Thermo Fisher Scientific MA5-12557 AB_10989883 Mouse 1:200
H2AX Millipore 05-636-I AB_2755003 Mouse 1:200
53BP1 Novus NB100-304 AB_10003037 Rabbit 1:200
Anti-mouse 488 Invitrogen A21202 AB_141607 Donkey 1:1000
Anti-mouse 568 Invitrogen A10037 AB_2534013 Donkey 1:1000
Anti-goat 657 Invitrogen A21447 AB_2535864 Donkey 1:1000
Anti-rabbit 647 Invitrogen A31573 AB_2536183 Donkey 1:1000
Anti-chicken 568 Invitrogen A11041 AB_2534098 Goat 1:1000
Anti-chicken 647 Invitrogen A21449 AB_2535866 Goat 1:1000
Anti-mouse 488 Invitrogen A11029 AB_2534088 Goat 1:1000
Anti-mouse 568 Invitrogen A11031 AB_144696 Goat 1:1000
Anti-mouse 647 Invitrogen A21236 AB_2535805 Goat 1:1000
Anti-rabbit 568 Invitrogen A11036 AB_10563566 Goat 1:1000
Anti-rabbit 647 Invitrogen A21244 AB_2535812 Goat 1:1000

Validation

All the antibodies used in this study are commercially available and validated. No further validation was performed from our side.

Eukaryotic cell lines

Policy information about cell lines and Sex and Gender in Research

Cell line source(s)

Details on the cell lines used are mentioned in the Supplementary Table 1.

Cell line source(s)	WT 1, Female, from GIBCO A13777<br>WT 2, Female, from Reinhardt et al., 2013<br>WT, male, from Promocell<br>Progerin, male, engineered and provided by the lab of Prof. Frank Edenhover in Innsbruck
Authentication	Cell lines have been karyotyped.
Mycoplasma contamination	Mycoplasma test was performed in all cultures every month. All the tests were negative for Mycoplasma contamination.
Commonly misidentified lines (See ICLAC register)	N/a

Plants

Seed stocks	*Report on the source of all seed stocks or other plant material used. If applicable, state the seed stock centre and catalogue number. If plant specimens were collected from the field, describe the collection location, date and sampling procedures.*
Novel plant genotypes	*Describe the methods by which all novel plant genotypes were produced. This includes those generated by transgenic approaches, gene editing, chemical/radiation-based mutagenesis and hybridization. For transgenic lines, describe the transformation method, the number of independent lines analyzed and the generation upon which experiments were performed. For gene-edited lines, describe the editor used, the endogenous sequence targeted for editing, the targeting guide RNA sequence (if applicable) and how the editor was applied.*
Authentication	*Describe any authentication procedures for each seed stock used or novel genotype generated. Describe any experiments used to assess the effect of a mutation and, where applicable, how potential secondary effects (e.g. second site T-DNA insertions, mosiacism, off-target gene editing) were examined.*

Flow Cytometry

Plots

Confirm that:

☒ The axis labels state the marker and fluorochrome used (e.g. CD4-FITC).

☒ The axis scales are clearly visible. Include numbers along axes only for bottom left plot of group (a 'group' is an analysis of identical markers).

☒ All plots are contour plots with outliers or pseudocolor plots.

☒ A numerical value for number of cells or percentage (with statistics) is provided.

Methodology

Sample preparation	Three embedded assembloids per condition were used for GFP+, live cells measurement in BD LSRFortessa flow cytometer (RRID:SCR_019601). Geltrex embedded assembloids were first incubated at 37oC for 40-50 min on shaker in 500 µl of papain solution containing 20 ml DMEMF12, 36 mg Papain (Sigma-Aldrich, P4762), 8 mg EDTA (Sigma-Aldrich, E6758) and 8 mg LCystein (Sigma-Aldrich, C6852). To start the dissociation process, papain solution was replaced with 500 µl accutase and the assembloids were pipetted with the 1000 pipette, followed by a 10 min incubation shaking. After that, pipetting with the 200 µl pipette and incubation cycles were continued until the complete dissociation of the assembloids. For the accutase and papain inhibition, 500 µl papain inhibitor solution containing 5 mg/ml BSA (Carl Roth, 8076.4) and 5 mg/ml Trypsin inhibitor (Sigma-Aldrich/Roche, 10109878001) in PBS was added. After transferring the total volume in 2 ml Eppendorf tube, the dissociated assembloids were centrifuged at 500xg for 5 min. Supernatant was discarded and the pellet was washed once with PBS. The pellet was resuspended in 300 µl DMEM (Thermo Fisher Scientific, A14430-01) containing 1:1000 concentration live-dead stain Zombie NIR (Biolegend, 423106), followed by incubation at 37oC for up to 30 min. Cells were then centrifuged at 500xg for 3 min and pellet was washed twice with PBS and centrifuged again with the same setting. After the final wash and centrifugation, the pellet was resuspended in DMEM and the samples were run in Becton Dickinson LSRFortessa, with 10000 events acquisition of GFP+, live-cells. Each sample was run in two technical replicates.<br><br>The analysis file from FlowJo is provided, where the gating strategy can be explored.
Instrument	BD<br>LSRFortessa flow cytometer (RRID:SCR_019601)
Software	FlowJo software (v.10.7.2, RRID:SCR_008520)
Cell population abundance	Cells were selected with FSC/SSC gating and GFP positive signal.
Gating strategy	Gating of the first population was performed via SSC-A/FSC-1. Then subsequent gating was performed with FSC-H/FSC-A to select single cells and exclude doublets. The boundary for the GFP positive cells was defined in the histogram with Comp-

FITC-A in the x axis higher or equal to 10^3. Before the experiment, compensation was performed using unstained and stained samples and sample without intrinsic GFP expression.

☒ Tick this box to confirm that a figure exemplifying the gating strategy is provided in the Supplementary Information.
